# Non-invasive spinal cord electrical stimulation for arm and hand function in chronic tetraplegia: a safety and efficacy trial

**DOI:** 10.1038/s41591-024-02940-9

**Published:** 2024-05-20

**Authors:** Chet Moritz, Edelle C. Field-Fote, Candace Tefertiller, Ilse van Nes, Randy Trumbower, Sukhvinder Kalsi-Ryan, Mariel Purcell, Thomas W. J. Janssen, Andrei Krassioukov, Leslie R. Morse, Kristin D. Zhao, James Guest, Ralph J. Marino, Lynda M. Murray, Jill M. Wecht, Markus Rieger, Jared Pradarelli, Amanda Turner, Jessica D’Amico, Jordan W. Squair, Gregoire Courtine

**Affiliations:** 1 Departments of Rehabilitation Medicine, Electrical & Computer Engineering, Physiology & Biophysics and Center for Neurotechnology, University of Washington, Seattle, WA USA; 2 Shepherd Center, Crawford Research Institute and Department of Rehabilitation Medicine, Emory University School of Medicine, Atlanta, GA USA; 3 Department of Physical Therapy, Craig Hospital, Englewood, CO USA; 4 Sint Maartenskliniek, Revalidatiegeneeskunde, Nijmegen, The Netherlands; 5 Department of Physical Medicine and Rehabilitation, Harvard Medical School, Boston, MA USA; 6 Spaulding Rehabilitation Hospital, Charlestown, MA USA; 7 KITE Research Institute|Toronto Rehab, University Health Network, Toronto, Ontario Canada; 8 Scottish Centre for Innovation in Spinal Cord Injury, Queen Elizabeth National Spinal Injuries Unit, Queen Elizabeth University Hospital, Glasgow, UK; 9 Amsterdam Rehabilitation Research Center | Reade, Amsterdam, The Netherlands; 10 Faculty of Behavioral and Movement Sciences, Vrije Universiteit Amsterdam, Amsterdam Movement Sciences, Amsterdam, The Netherlands; 11 ICORD and Division of Physical Medicine and Rehabilitation, University of British Columbia, Vancouver, British Columbia Canada; 12 Department of Rehabilitation Medicine, University of Minnesota School of Medicine, Minneapolis, MN USA; 13 Department of Physical Medicine and Rehabilitation, Rehabilitation Medicine Research Center, Mayo Clinic, Rochester, MN USA; 14 Department of Neurological Surgery, Miller School of Medicine, University of Miami, Miami, FL USA; 15 Miami Project to Cure Paralysis, Miami, FL USA; 16 Thomas Jefferson University Hospital, Philadelphia, PA USA; 17 Departments of Rehabilitation and Human Performance and Medicine, James J. Peters VA Medical Center, Icahn School of Medicine at Mount Sinai, New York, NY USA; 18 Department of Research and Development, James J. Peters VA Medical Center, Icahn School of Medicine at Mount Sinai, New York, NY USA; 19 ONWARD Medical, Lausanne, Switzerland; 20 Glenrose Rehabilitation Hospital, Alberta Health Services, Edmonton, Alberta Canada; 21 Department of Medicine, University of Alberta, Edmonton, Alberta Canada; 22 NeuroX Institute and Brain Mind Institute, School of Life Sciences, Swiss Federal Institute of Technology (EPFL), Geneva, Switzerland; 23 Department of Clinical Neuroscience, Lausanne University Hospital (CHUV) and University of Lausanne (UNIL), Lausanne, Switzerland; 24 Defitech Center for Interventional Neurotherapies (NeuroRestore), EPFL/CHUV/UNIL, Lausanne, Switzerland; 25 NeuroRestore, NeuroX Institute, School of Life Sciences, Swiss Federal Institute of Technology (EPFL), Lausanne, Switzerland

**Keywords:** Spinal cord diseases, Spinal cord, Regeneration and repair in the nervous system

## Abstract

Cervical spinal cord injury (SCI) leads to permanent impairment of arm and hand functions. Here we conducted a prospective, single-arm, multicenter, open-label, non-significant risk trial that evaluated the safety and efficacy of ARC^EX^ Therapy to improve arm and hand functions in people with chronic SCI. ARC^EX^ Therapy involves the delivery of externally applied electrical stimulation over the cervical spinal cord during structured rehabilitation. The primary endpoints were safety and efficacy as measured by whether the majority of participants exhibited significant improvement in both strength and functional performance in response to ARC^EX^ Therapy compared to the end of an equivalent period of rehabilitation alone. Sixty participants completed the protocol. No serious adverse events related to ARC^EX^ Therapy were reported, and the primary effectiveness endpoint was met. Seventy-two percent of participants demonstrated improvements greater than the minimally important difference criteria for both strength and functional domains. Secondary endpoint analysis revealed significant improvements in fingertip pinch force, hand prehension and strength, upper extremity motor and sensory abilities and self-reported increases in quality of life. These results demonstrate the safety and efficacy of ARC^EX^ Therapy to improve hand and arm functions in people living with cervical SCI. ClinicalTrials.gov identifier: NCT04697472.

## Abstract (web summary)

Externally applied electrical stimulation over the cervical spinal cord improves arm and hand functions in people with chronic tetraplegia due to spinal cord injury.

## Main

Spinal cord injury (SCI) disrupts the bidirectional communication between the regions of the brain and spinal cord that produce and regulate essential neurological functions^1–6^. When the SCI occurs in the cervical segments, the consequence is often irreversible impairment of arm and hand functions.

Preclinical studies demonstrated that electrical stimulation of the spinal cord restores impaired neurological functions when the stimulation is applied over the spinal segments that contain the neurons involved in the control of these functions^7–17^. Case studies leveraged this principle in humans with SCI, reporting immediate improvements in a range of neurological functions in response to electrical stimulation of the spinal cord, including standing and walking^8,18–24^, muscle spasms^25,26^, hemodynamic regulation^12,19,27^, lower urinary tract control^28–30^ and the function of the arms and hands^31–36^. Moreover, the long-term application of electrical stimulation to the spinal cord during rehabilitation led to neurological improvements that persisted in the absence of stimulation^8,24,33,34,37^. Similar improvements have been observed in people with stroke^38^. Evidence suggests that these neurological improvements are due to the growth of residual white matter tracts onto specific neuronal populations that are engaged by afferent pathways recruited by electrical stimulation and that reorganize in response to rehabilitation^7,8,10,39–41^.

**Fig. 1:**
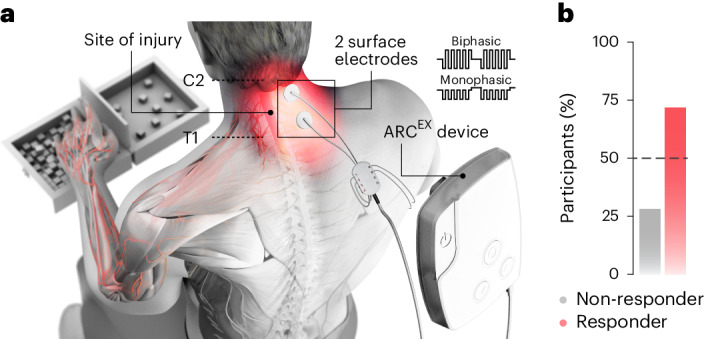
Overview and efficacy of ARC^EX^ Therapy. **a**, ARC^EX^ Therapy consists of delivering externally applied electrical stimulation to the cervical spinal cord during structured rehabilitation. The stimulating electrodes are located above and below the injury. **b**, The primary effectiveness endpoint tested the hypothesis that the majority of the participants would demonstrate significant improvements in selected strength and functional performance domains from the end of the rehabilitation-alone period to the end of the ARC^EX^ Therapy period.

Stimulation of the spinal cord can be achieved using non-invasive methodology whereby electrical current is delivered to the spinal cord through surface electrodes, so as to modulate neuronal subpopulations within the targeted spinal segments through the recruitment of afferent fibers where they enter in the spinal cord^32,34,42^. The ARC^EX^ device has been engineered for the delivery of such stimulation and is under investigation for the improvement of arm and hand functions after chronic cervical SCI (Fig. 1a). We conducted a pivotal trial (Up-LIFT) to assess the safety of ARC^EX^ Therapy to modulate the activity of the cervical spinal cord and the effectiveness of ARC^EX^ Therapy to improve arm and hand functions compared to rehabilitation alone. Here we report the results of this prospective, single-arm, multicenter, open-label, non-significant risk trial (Fig. 1b).

## Results

### Patient disposition

**Fig. 2:**
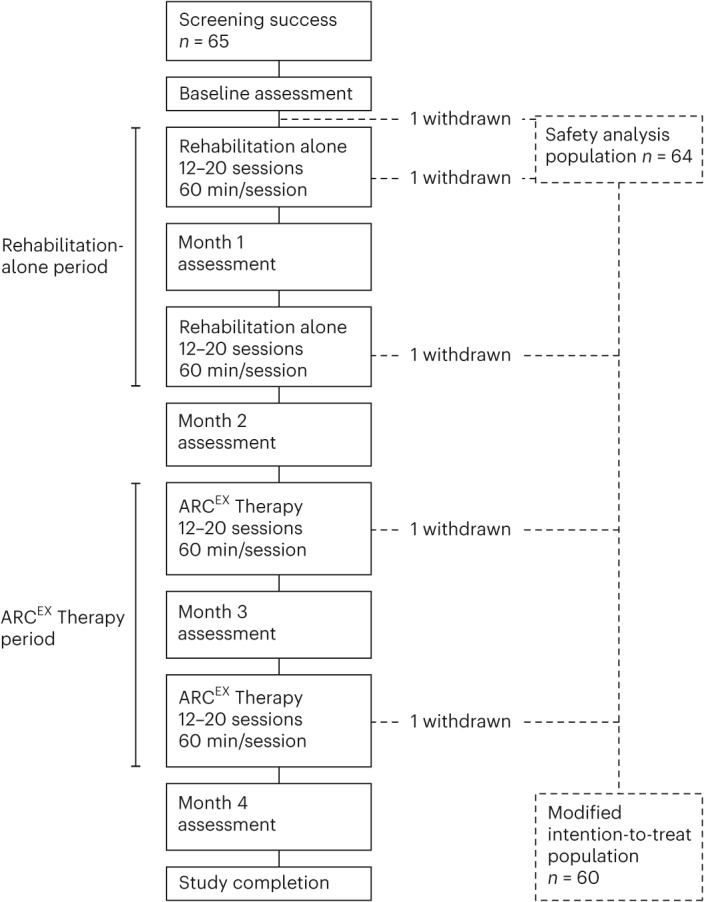
Screening and treatment exposure during the Up-LIFT trial. The Up-LIFT trial was a prospective, single-arm, multicenter, non-significant risk trial designed to evaluate the safety and efficacy of ARC^EX^ Therapy to improve arm and hand functions in people with chronic cervical SCI. Participants were screened and enrolled after a baseline assessment. They then underwent a period of rehabilitation alone followed by ARC^EX^ Therapy for the same period of time. Participants were considered to have completed the trial after finishing all sessions of rehabilitation alone, ARC^EX^ Therapy and all study assessments.

**Extended Data Fig. 1:**
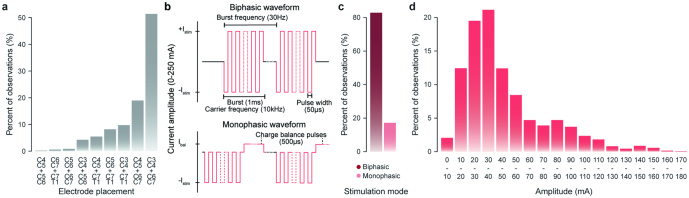
Location and stimulation parameters across study participants and sessions. **a**, Location of the two cathodes with respect to spinal segments. **b**, Profile of electrical stimulation waveforms. **c**, Percent of sessions with monophasic versus biphasic stimulation models. **d**, Range of amplitudes delivered during ARC^EX^ Therapy.

From 14 January to 24 December 2021, a total of 65 participants underwent screening and were enrolled in the Up-LIFT trial (Fig. 2). By the end of June 2022, 60 participants had completed the entire protocol and assessments. One participant withdrew from the study before any study procedures; two withdrew during the rehabilitation-alone period for personal reasons unrelated to the study; and two withdrew during the ARC^EX^ Therapy period, one due to protocol non-adherence and one for personal reasons. The clinical database was locked for analysis in July 2022. The 60 participants who completed the protocol had each undergone at least 24 sessions during each of the rehabilitation-alone (mean, 25 sessions) and ARC^EX^ Therapy (mean, 25 sessions) periods. During ARC^EX^ Therapy sessions, stimulation was delivered at 30 Hz with a 10-kHz carrier frequency overlay, which consisted of 10 pulses with a 10-kHz frequency and 100-µs pulse width (Fig. 1a and Extended Data Fig. 1). The 60 participants were included in the final primary effectiveness endpoint analysis. The demographic and baseline clinical characteristics of the 60 participants are reported in Table 1. Demographic representation within the clinical trial population was in line with the general population of people living with cervical SCI^43^. The Up-LIFT trial was fully enrolled within 1 year, and follow-up assessments were completed according to the trial design, despite constraints on clinic and hospital services related to the coronavirus disease 2019 (COVID-19) pandemic.

**Table 1:** Characteristics of the participants at baseline^a^

Characteristic	Safety population	Modified intention-to-treat population
	(*n* = 64)	(*n* = 60)
Age (years)	46.5 ± 15.5	47.2 ± 15.5
Age at time of injury (years)	40.6 ± 16.1	41.1 ± 16.2
Sex, *n* (%)		
Female	11 (17.2)	10 (16.7)
Male	53 (82.8)	50 (83.3)
Race, *n* (%)^b^		
Asian	3 (4.7)	3 (5.0)
Asian–White	1 (1.6)	1 (1.7)
Black or African American	1 (1.6)	1 (1.7)
Other	2 (3.1)	1 (1.7)
White	57 (89.1)	54 (90.0)
Ethnicity, *n* (%)		
Hispanic or Latino	4 (6.3)	3 (5.0)
Non-Hispanic or Latino	60 (93.8)	57 (95.0)
AIS, *n* (%)		
B	10 (15.6)	9 (15.0)
C	30 (46.9)	28 (46.7)
D	24 (37.5)	23 (38.3)
Cause of injury, *n* (%)		
Fall	16 (25.0)	15 (25.0)
Other	3 (4.7)	2 (3.3)
Recreation	14 (21.9)	14 (23.3)
Sport	17 (26.6)	17 (28.3)
Vehicular	14 (21.9)	12 (20.0)
Time since injury (years)	5.9 ± 7.3	6.1 ± 7.5
Chronicity, *n* (%)		
1–5 years	44 (68.8)	41 (68.3)
5–10 years	12 (18.8)	11 (18.3)
>10 years	8 (12.5)	8 (13.3)
Neurological level, *n* (%)		
C2	9 (14.1)	9 (15.0)
C3	4 (6.3)	3 (5.0)
C4	18 (28.1)	16 (26.7)
C5	13 (20.3)	13 (21.7)
C6	13 (20.3)	13 (21.7)
C7	6 (9.4)	6 (10.0)
C8	1 (1.6)	0

^a^ Plus–minus values are means ± s.d. The modified intention-to-treat population included all participants who underwent at least 24 sessions (minimum 12 sessions per month) during the rehabilitation-alone period and at least 24 sessions during the ARC^EX^ Therapy period.

^b^ Race was reported by the participants.

### Primary outcomes

**Extended Data Fig. 2:**
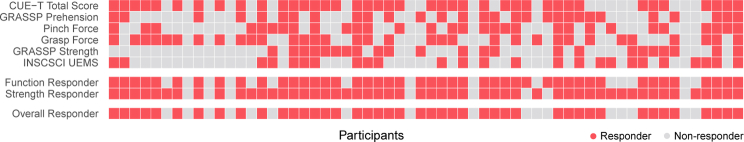
Responder status for each outcome of the strength and functional domains. Participants were considered responders for each outcome (top panel) if they met the minimally important difference (MID), which was calculated as the change in score between the beginning of the ARC^EX^ Therapy period and the end of the ARC^EX^ Therapy period. The beginning of the ARC^EX^ Therapy period coincides with the end of the rehabilitation alone period. Outcomes related to the functional domain included the Capabilities of Upper Extremity Test^68^ (CUE-T; MID = 4-point improvement) and the GRASSP Prehension Performance score^64^ (MID = 2-point improvement). Outcomes related to the strength domain included the Pinch force (MID = greater than or equal to 2.4N improvement), Grasp force (MID = greater than or equal to 6N improvement), the GRASSP Strength score^64^ (GRASSP-Strength; MID = 4-point improvement), and the International Standards for Neurological Classification of Spinal Cord Injury Upper Extremity Motor Score^67^ (ISNCSCI-UEMS; MID = 2-point improvement). To be classified as a ‘Function Responder’ or ‘Strength Responder’ participants must have met the MID criteria for at least one outcome in each domain. To be considered an ‘Overall Responder’, participants must have been classified as both a ‘Function Responder’ and a ‘Strength Responder’. Color indicates responder status for each row.

**Extended Data Fig. 3:**
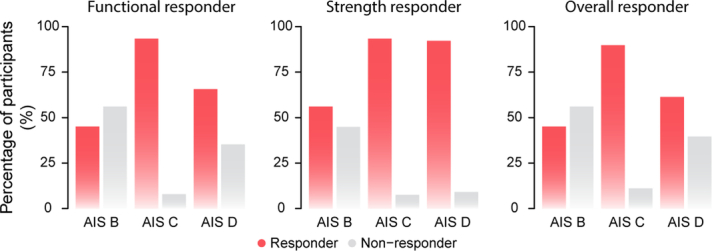
Influence of injury severity. The percentage of participants classified as responders versus non-responders are classified based on their American Spinal Injury Association Impairment Scale (AIS) at enrollment.

**Extended Data Fig. 4:**
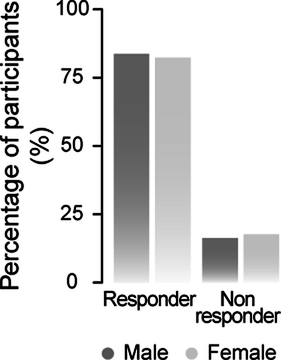
Influence of sex. The percentage of participants classified as responders versus non-responders are classified by sex.

Of the 60 participants included in the primary effectiveness endpoint analysis, 43 (72%) met or exceeded the minimally important difference (MID) criteria for at least one outcome of the strength domain (International Standards for Neurological Classification of Spinal Cord Injury-Upper Extremity Measurement Scale (ISNCSCI-UEMS), Graded Redefined Assessment of Strength, Sensibility and Prehension (GRASSP)-Strength, grasp force or pinch force) and at least one outcome of the functional performance domain (Capabilities of Upper Extremity Test (CUE-T) score or GRASSP-Prehension Performance), while 54 participants (90%) met the MID criteria for at least one strength or functional outcome (Fig. 1b, Table 2 and Extended Data Figs. 2–4).

**Table 2:** Effectiveness endpoints (modified intention-to-treat population)^a^

Endpoints	Value	*P* value
**Primary endpoint**^b^		
Strength responder, *n* (%)	52 (86.7)	
Function responder, *n* (%)	45 (75.0)	
Responder, *n* (%)	43 (71.7)	< 0.001
**Secondary responder analysis endpoint**^c^		
ARC^EX^ non-responder, *n* (%)		
Rehabilitation responder	4 (6.7)	
Rehabilitation non-responder	6 (10.0)	
ARC^EX^ responder, n (%)		
Rehabilitation responder	34 (56.7)	
Rehabilitation non-responder	16 (26.7)	0.012
	**Change from end of rehabilitation alone to end of ARC**^**EX**^ **Therapy**	***P*** **value**
**Secondary hierarchical endpoint**^d^		
Pinch force	4.8 ± 16.1	0.002*
GRASSP-Prehension Performance	1.6 ± 2.9	<0.001
GRASSP-Strength	2.8 ± 5.4	<0.001*
UEMS	2.2 ± 3.2	<0.001*
TSS	9.6 ± 15.1	<0.001
EQ-5D-5L	1.7 ± 14.1	0.028*
SCIM III total score	0.5 ± 4.2	0.101*
WHOQOL-Physical Domain	1.6 ± 8.0	−
WHOQOL-Psychological Domain	−0.4 ± 9.4	−
WHOQOL-Social Relationships	1.5 ± 10.3	−
WHOQOL-Environment	−0.4 ± 7.5	−

^a^ Plus–minus values are means ± s.d. The modified intention-to-treat population included all participants who underwent at least 24 sessions (minimum 12 sessions per month) during the rehabilitation-alone period and at least 24 sessions during the ARC^EX^ Therapy period.

^b^ The primary effectiveness endpoint tested the hypothesis that more than 50% of participants would meet responder criteria for both strength and function. This hypothesis was evaluated using a one-sided exact binomial test.

^c^ Superiority of ARC^EX^ Therapy compared to rehabilitation alone was tested by comparing the proportion of participants who converted into responders from enrollment to the end of the rehabilitation-alone period with the proportion of participants who converted from enrollment to the end of the ARC^EX^ Therapy period. This secondary endpoint was tested using McNemar’s test.

^d^ Changes in individual outcomes were assessed from enrollment to the end of ARC^EX^ Therapy compared to changes from enrollment to the end of rehabilitation alone using a one-sided paired *t*-test or a paired Wilcoxon rank-sum test if data were not normal. The order of the secondary endpoints according to the hierarchical analysis plan is also provided in the Supplementary Information. The hierarchy failed at the seventh endpoint (Spinal Cord Independence Measure, Version III (SCIM III) total score). Dashes indicate *P* values that are not provided due to failure of the hierarchical testing of results. * indicates a parametric test.

### Secondary outcomes

Secondary effectiveness endpoints included the superiority of responder rates after ARC^EX^ Therapy compared to rehabilitation alone as well as the changes in single outcomes between enrollment and the end of the rehabilitation-alone period versus between enrollment and the end of the ARC^EX^ Therapy period.

**Fig. 3:**
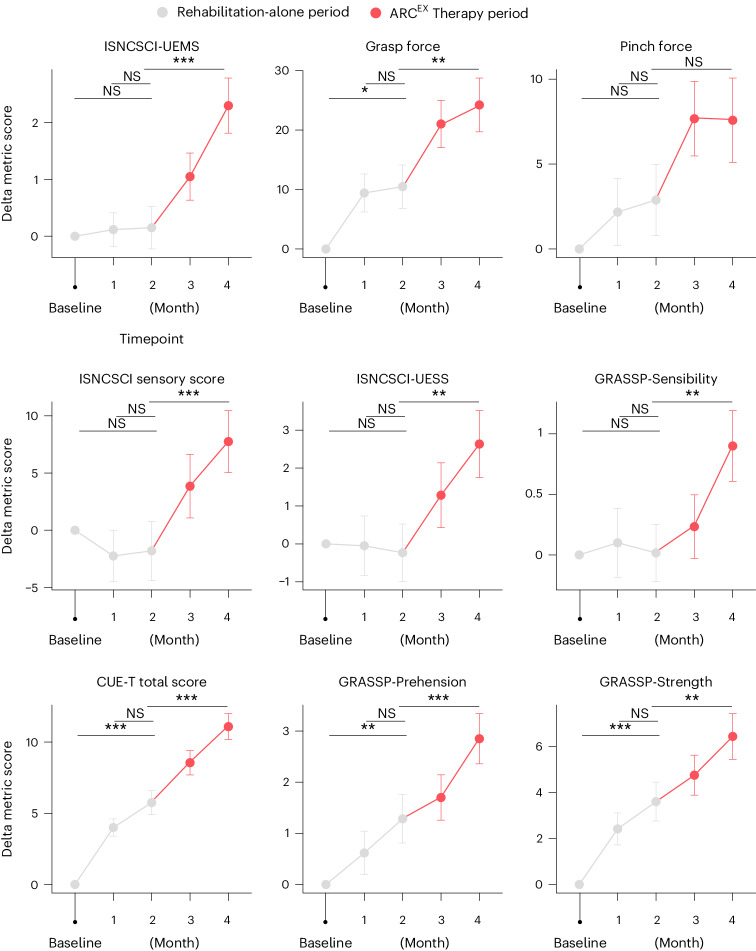
Effect of ARC^EX^ Therapy on force, sensory and functional performance. Improvements in outcomes of strength and functional performance domains during the rehabilitation-alone period and during the ARC^EX^ Therapy period. These results suggest that a longer period of ARC^EX^ Therapy may promote additional benefits. Red color indicates the period of ARC^EX^ Therapy. Statistics represent one-way repeated-measures ANOVA with Tukey’s HSD post hoc testing. *P < 0.05, **P < 0.01 and ***P < 0.001. NS, not significant.

**Extended Data Fig. 5:**
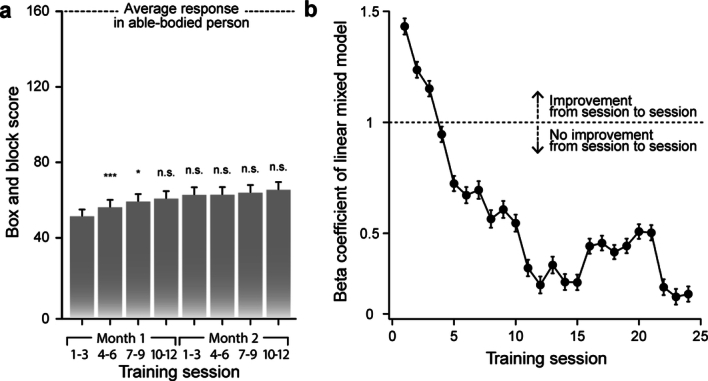
Improvements of hand and arm functions plateau in response to intense rehabilitation well before the end of the rehabilitation alone period. **a**, During each training session, the participant completed the box and block test. These systematic quantifications allowed to monitor improvements in this task over the rehabilitation alone period. During the first three weeks of the rehabilitation alone period, we detected a significant increase in the scores in the box and block test, normalized to the baseline score at enrollment for each participant. No statistically significant improvement of scores was detected during the following 5 weeks of the rehabilitation alone period. Statistics refers to a repeated measures one-way ANOVA with post hoc testing using the Tukey HSD method. * indicates p < 0.05. ** indicates p < 0.01. *** indicates p < 0.001. n.s. indicates non-significant. Bar graph indicates mean and standard error of the mean for each time point. Statistics provided in Supplementary Data 3. **b**, A rolling linear regression coefficient was calculated from the score of each box and block test for each participant using a mixed model linear regression. The dotted line coincides with a coefficient of one, above which improvements remain linear. Dot represents the coefficient of the linear model at each timepoint, and the whiskers represent the standard error of the mean on this model. The linear relationship between training sessions and improvements of scores in the box and block test vanished after three weeks (12 sessions) of rehabilitation alone, wherein the coefficient approached 0. Together these findings reveal the occurrence of a plateau in the improvement of arm and hand functions after three weeks of rehabilitation alone. Since participants performed the box and block test during each session, the initial improvement observed may be partially attributed to increased familiarity with the test through repeated practice.

**Extended Data Fig. 6:**
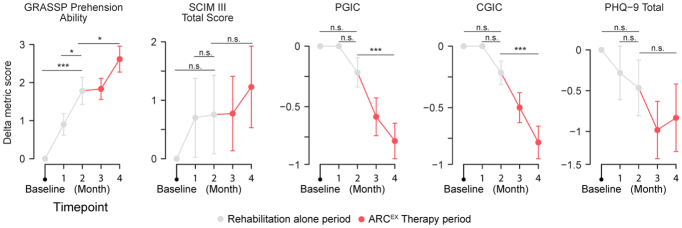
Effect of ARC^EX^ Therapy on additional secondary outcomes. Improvements in secondary outcome domains during the rehabilitation alone period, and during the ARC^EX^ Therapy period. Lower values of PGIC, CGIC and PHQ-9 represent improved quality of life. These results suggest that a longer period of ARC^EX^ Therapy may promote additional benefits. Red color indicates the period of ARC^EX^ Therapy. Statistics represent one-way repeated measures ANOVA with Tukey HSD post-hoc testing. * = p < 0.05. ** = p < 0.01. *** = p < 0.001. Line graphs represent the mean and standard error of the mean for each outcome measure. Statistics provided in Supplementary Data 3.

Structured rehabilitation is a methodology that can mediate improvement in arm and hand functions for people with chronic tetraplegia^44–46^. These improvements, however, are generally confined to functional domains, and bona fide changes in the underlying neurological status are not expected. Accordingly, we found that 63% of the participants met the MID responder criteria for improvements in arm and hand functions in response to the 2-month period of rehabilitation alone. This response rate was inferior to the response rate after the ARC^EX^ Therapy period (*P* = 0.012, McNemar’s test). As anticipated, most of the gain occurred during the first month of rehabilitation alone and primarily involved expected improvements in functional domains. Indeed, participants did not show significant improvements in standard neurological assessments, including upper limb motor and sensory scores, in response to rehabilitation alone (Fig. 3 and Extended Data Table 1). Moreover, analysis of all the individual outcomes between the first and second month of rehabilitation alone revealed an absence of significant improvement, indicating that a number of participants showed initial improvements but reached a rapid plateau that occurred before the onset of ARC^EX^ Therapy (Fig. 3 and Extended Data Fig. 5). These improvements were in stark contrast to those observed after ARC^EX^ Therapy, wherein significant improvements in functional domains and neurological status, including both upper limb motor and sensory scores, were observed throughout the period with ARC^EX^ Therapy (Fig. 3, Table 2, Extended Data Figs. 5 and 6, Extended Data Table 2 and Supplementary Data 1).

Table 2 and Fig. 3 report the improvements across individual outcomes that were measured monthly for each of the hierarchical secondary effectiveness endpoints and additional strength, function and sensory outcomes obtained for all the participants who completed the Up-LIFT trial. These comparisons revealed significant improvements over the course of ARC^EX^ Therapy in pinch force (mean difference = 4.8 N; 90% confidence interval (CI) = 1.25–8.44 N; *P* = 0.002), GRASSP-Prehension Performance score (mean difference = 1.6; 90% CI = 0.9–2.2; *P* < 0.001), GRASSP-Strength score (mean difference = 2.8; 90% CI = 1.6–3.9; *P* < 0.001) and ISNCSCI-UEMS (mean difference = 2.2; 90% CI = 1.5–2.8; *P* < 0.001). In addition to strength and functional performance domains, a significant increase in ISNCSCI total sensory score (TSS) (mean difference = 9.6; 90% CI = 6.3–12.8; *P* < 0.001) was detected in response to ARC^EX^ Therapy compared to rehabilitation alone. Additionally, exploratory statistical comparisons quantifying the magnitude of improvements obtained after rehabilitation alone compared to those obtained after an equivalent period of ARC^EX^ Therapy revealed the superiority of ARC^EX^ Therapy (Fig. 3 and Extended Data Fig. 6).

Improvements in strength, functional performance and sensory scores were associated with self-reported improvements in EuroQol five-dimensional five-level (EQ-5D-5L) scores (mean difference = 1.7; 90% CI = −1.3 to 4.8; *P* < 0.028; Table 2) in response to ARC^EX^ Therapy compared to rehabilitation alone. Improvements in independence, as measured with the SCIM III, failed to meet significance in the hierarchical statistical analysis. Due to the hierarchical statistical plan, changes in quality of life measured with the Abbreviated World Health Organization Quality of Life (WHOQOL-BREF) questionnaire were not tested, yet 52 (87%) of participants reported improvements in at least one WHOQOL-BREF subscore or the EQ-5D-5L questionnaire (Table 2 and Extended Data Table 3).

### Safety

No serious adverse events were reported that were related to either ARC^EX^ Therapy or study procedures in any of the 64 participants who were exposed to any procedures of the Up-LIFT trial (Table 3). A total of 238 adverse events occurred throughout the duration of the study (Table 3, Extended Data Table 3 and Supplementary Data 2). These adverse events were reported in 50 of the 64 individuals who were exposed to any aspect of the Up-LIFT trial procedures. Three of these 238 adverse events were considered serious due to hospitalization associated with the event yet were unrelated to ARC^EX^ Therapy or study procedures. They included constipation, urinary tract infections and bladder stone. Forty-four non-serious adverse events were related to ARC^EX^ Therapy and were reported in 17 of the 64 participants (Table 3). One of these 44 events was a severe adverse event, which was reported in one participant and was related to the occurrence of severe muscle spasms during one rehabilitation session within the ARC^EX^ Therapy period, but it was unrelated to ARC^EX^ Therapy because the stimulation was not turned on when these spasms occurred. There were no unexpected adverse events.

**Table 3:** Overview of adverse events (safety population)^a^

Event	Before rehabilitation	Rehabilitation alone	ARC^EX^ Therapy	Any time
Any adverse event	3 (4.7)	44 (68.8)	34 (53.1)	50 (78.1)
Any serious adverse event	1 (1.6)	1 (1.6)	1 (1.6)	3 (4.7)
Adverse event related to device	–	–	17 (26.6)	17 (26.6)
Serious adverse event related to device	–	–	0 (0.0)	0 (0.0)
Adverse event related to study procedures	0 (0.0)	8 (12.5)	13 (20.3)	18 (28.1)
Adverse event leading to discontinuation	0 (0.0)	0 (0.0)	0 (0.0)	0 (0.0)
Total number of adverse events	5	105	128	238

^a^ Data shown are the number of participants (percent). The safety population included participants (*n* = 64) exposed to any study procedure.

### Exploratory outcomes

Extended Data Tables 1, 2 and 4 report the changes in single outcomes for each of the pre-specified exploratory endpoints for all the participants who completed the Up-LIFT trial, as outlined in the statistical plan. Among numerous significant improvements in exploratory outcomes, we observed a significant decrease in the frequency of muscle spasms, as measured with the Penn Spasm Frequency Scale (PSFS) (mean difference = −0.2; 90% CI = −0.4 to 0.1; *P* = 0.009), improvement in sleep quality (Medical Outcomes Study (MOS) Sleep Scale) (mean difference = −4.33; 90% CI = −8.0 to −0.7; *P* = 0.025) and shortness of breath (Sleep Problems Index I (mean difference = −2.3; 90% CI = −4.5 to 0.2; *P* = 0.04)) and a reduction in pain (Numerical Rating Scale (NRS) of pain, mean difference = −0.2; 90% CI = −0.6 to 0.1; *P* = 0.04). Indeed, 51 participants (85%) reported improvements in at least one MOS Sleep Scale subscore.

### Post hoc analyses

**Extended Data Fig. 7:**
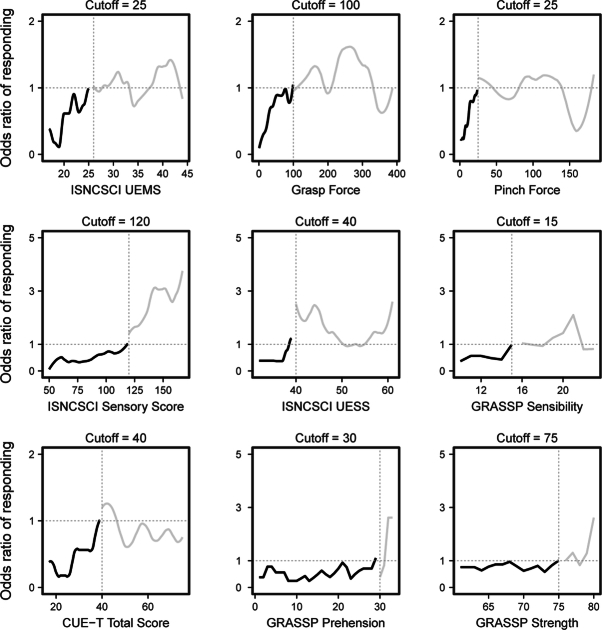
Identification of initial baseline characteristics that best predicted responder status. This analysis included sequential logistic regression models whereby participants were binarized into two groups: above or below a single numerical threshold^78,79^. Odds ratios were then calculated, which reflected the odds of being a responder based on sequential thresholds for each outcome measure included in the primary and secondary effectiveness end points. The sequential models were halted when the odds ratio crossed 1 (black traces), indicating a threshold above which participants demonstrated positive odds of responding to ARC^EX^ Therapy. This analysis revealed cutoffs for ISNCSCI-UEMS (cutoff = 25), Grasp force (cutoff = 100N), Pinch force (cutoff = 25N), CUE-T (cutoff = 40), ISNCSCI Sensory Score (cutoff = 120), ISNCSCI Upper Extremity Sensory Score (cutoff = 40), and GRASSP-Sensibility score (cutoff = 15).

We conducted a post hoc analysis based on logistic regression odds ratios to assess the minimal value at enrollment for each strength, functional performance and sensory scores associated with the likelihood of responding to ARC^EX^ Therapy (Extended Data Fig. 7). This analysis revealed cutoffs for ISNCSCI-UEMS (cutoff = 25), pinch force (cutoff = 25 N), grasp force (cutoff = 100 N), CUE-T (cutoff = 40), ISNCSCI sensory score (cutoff = 120), ISNCSCI upper extremity sensory score (cutoff = 40) and GRASSP-Sensibility score (cutoff = 15). We also found that participants improved more in the box and block test following the period of ARC^EX^ compared to rehabilitation alone (*P* < 0.001).

## Discussion

ARC^EX^ Therapy was found to be safe and effective in 72% of participants to mediate improvements of strength and function in the hands and arms that were associated with meaningful quality of life improvements for people living with chronic cervical SCI. These results met our pre-specified criteria, because we hypothesized that the percentage of participants responding to ARC^EX^ Therapy would exceed 50%.

We found that ARC^EX^ Therapy not only mediated significant improvements in outcomes related to upper extremity strength and functional performance domains but also improved the recovery of sensory function, as measured with the ISNCSCI TSS and the GRASSP-Sensibility score. Participants also reported a decrease in the frequency and severity of muscle spasms, improved sleep quality and reduced pain. These improvements translated into significant increases in overall well-being, measured with the EQ-5D-5L, as well as outcomes assessing level of independence during activities of daily living, such as improved self-care components relying on arm and hand functions.

ARC^EX^ Therapy mediated improvements in upper limb functions that exceeded those achieved with rehabilitation alone. Although participants in the Up-LIFT trial demonstrated some improvement in measures of arm and hand functions after a period of rehabilitation alone, their neurological status improved only when delivering ARC^EX^ Therapy. Indeed, all the clinical quantifications of upper extremity motor and sensory scores improved only after ARC^EX^ Therapy. Based on the design of the trial, however, one cannot exclude the possibility that the period of rehabilitation alone improved the potential for participants to respond subsequently to ARC^EX^ Therapy. Future preclinical and clinical studies will have to uncover the mechanisms that govern the relationship between the neurological status of the participant and the responses to ARC^EX^ Therapy or rehabilitation alone as well as their interactions. Finally, we also identified the minimal values of strength, functional and sensory domains at enrollment that can guide rehabilitation and neurological specialists in the selection of patients who would optimally benefit from ARC^EX^ Therapy.

The safety profile of ARC^EX^ Therapy was thoroughly established in the Up-LIFT trial. The incidence and nature of adverse events reported in the trial were consistent with published reports on people living with chronic cervical SCI^47,48^. The absence of any device-related serious adverse events establishes that ARC^EX^ Therapy meets the pre-specified primary and secondary safety endpoints.

The design and planning of this clinical trial was conducted with several considerations and potential limitations. Current standard of care for every patient with tetraplegia consists of a period of rehabilitation that is initiated after discharge from neurointensive care and typically lasts for a few months. Although the delivery of rehabilitation is supported by decades of research studies and clinical practice, the impact of rehabilitation on neurological status is expected to be limited and, in general, confined to functional improvements as opposed to improvement of the underlying neurological status^49^. Because of this limited effect, rehabilitation is generally not prescribed to patients once they have reached the chronic phase of tetraplegia^50^. Furthermore, neurological recovery in patients with tetraplegia is known to be as variable as the morphology and location of spinal cord damage, even among those with similar neurological classifications^51^. Consequently, the limited impact of rehabilitation alone on neurological recovery, coupled with the inherent variation in function of people with tetraplegia, did not support a standard randomized controlled trial design.

In the context of trials involving neuromodulation therapies, where the patients can physically feel the stimulation, consultations with the study investigators exposed four compelling reasons for an open-label design. First, the feasibility of blinding becomes challenging, if not impossible, when the treatment requires the participants to perceive the electrical fields produced by the neuromodulation therapies^52–56^. This robust perception elicited by treatments such as ARC^EX^ Therapy is in stark contrast to neuromodulation treatments for which there is no explicit sensation, wherein sham stimulation is generally standard practice in clinical trial design^57–59^. ARC^EX^ Therapy involves electrical fields that are perceived by patients, leading to immediate improvements in manual dexterity that support enhanced participation in rehabilitation^33,34^. If the stimulation is turned off, patients instantly notice the absence of stimulation and the lack of facilitated movement. On the other hand, because the amplitude of stimulation is the primary parameter that determines the facilitation of movement, there are few alternative parameters of stimulation that could be manipulated to elicit a perception of the stimulation by the patient without facilitating movement to some extent. Second, ethical considerations were raised about the role of sham stimulation, because subjecting participants to potential risks and discomfort without any expected benefit was deemed not appropriate for the population of people with tetraplegia for whom there are no available treatments. Therefore, it was not realistic or appropriate to design a relevant sham stimulation. Third, the sequential trial design enabled strong participant engagement, compliance and low attrition. Individuals with tetraplegia typically experience difficulties participating in trials with repeated sessions at rehabilitation centers. Fourth, this design provided the opportunity to gather valuable data on the participants’ subjective experiences, including sensation of stimulation and any side effects.

We also considered a randomized cross-over design, where the order of rehabilitation alone and rehabilitation augmented by ARC^EX^ Therapy was randomized for each participant. In this design, however, any data collected after rehabilitation augmented by ARC^EX^ Therapy are likely to be affected by the lasting benefits of this treatment. Indeed, sustained neurological improvements have been reported to last for at least 3–6 months after ARC^EX^ Therapy^8,24,33,34,60^, even after cessation of the treatment.

The additional limitations of the Up-LIFT trial primarily concern the time at which ARC^EX^ Therapy was initiated and the duration over which ARC^EX^ Therapy was delivered to the participants. Indeed, formal monthly assessments of all 60 participants revealed that, after 2 months of ARC^EX^ Therapy, the participants had not reached a plateau in their functional recovery. This absence of plateau suggests that extending the duration of ARC^EX^ Therapy beyond the arbitrary 2 months pre-specified in the Up-LIFT trial may not only mediate further improvements in strength, functional performance and sensation in people responding to ARC^EX^ Therapy but may also enable participants who did not respond to ARC^EX^ Therapy to meet the pre-established responder criteria if provided with sufficient exposure to the therapy. Second, all the recruited participants had experienced an SCI at least 1 year but up to 34 years before their enrollment in the Up-LIFT trial. Preclinical studies have shown that a window of opportunity for enhanced reorganization of residual neuronal pathways opens shortly after an SCI and that this reorganization augments neurological recovery. This window closes approximately 1 year after injury in humans^61^. Therefore, ARC^EX^ Therapy may not only accelerate the recovery of arm and hand functions but may also augment the extent of neurological improvements when delivered in the early phase after SCI. Although these possibilities were not explored in the Up-LIFT trial, post-market analyses and post-market clinical studies will enable the opportunity to address these hypotheses. Finally, future investigations involving both preclinical models and follow-up clinical studies must explore the mechanisms responsible for the immediate and long-term improvement of arm and hand functions in response to ARC^EX^ Therapy and how the stimulation interacts with structured rehabilitation. These studies will support the optimization of ARC^EX^ Therapy and will guide the design of trials exploring the application of ARC^EX^ Therapy to improve additional neurological functions in people living with SCI.

The Up-LIFT trial demonstrates the safety and efficacy of ARC^EX^ Therapy for the improvement of hand and arm functions in people living with chronic cervical SCI. If approved by regulatory authorities, ARC^EX^ Therapy will serve as a new treatment with established safety and efficacy to improve the neurological recovery of hand and arm functions. Based on the impact of spinal cord stimulation on the recovery of movement after stroke^38^ and Parkinson’s disease^62^, we anticipate that ARC^EX^ Therapy could also play a role in augmenting the recovery of people suffering from a range of neurological disorders.

## Methods

### Trial oversight

We conducted this trial according to the principles of the Declaration of Helsinki and Good Clinical Practice guidelines. The trial was conducted at 14 sites located in the United States, Canada, United Kingdom and The Netherlands. The trial and recruitment materials were approved by institutional review boards or ethics committees at each trial site as well as central approval from the Advarra institutional review board. An independent Data Safety Monitoring Board regularly reviewed the ongoing trial and could advise the sponsor to stop the trial for safety. The trial was sponsored by ONWARD Medical, which managed the trial through contract research organizations, provided the ARC^EX^ devices and provided field support for study investigators. Statistical analysis was performed by an independent statistician. All participants provided written informed consent.

### Participants

The trial included adult participants aged 22–75 years who had sustained a traumatic, non-progressive cervical (C2–C8) SCI more than 12 months before their enrollment. Only participants with American Spinal Injury Association (ASIA) Impairment Scale (AIS) classification^63^ B, C or D who presented with a GRASSP-Prehension Performance^64^ score greater than or equal to 10 or a GRASSP-Strength^64^ score greater than or equal to 30 were considered for enrollment. The participants who were prescribed anti-spasticity medications had to reduce their total baclofen dose to less than 30 mg per day before enrollment if needed and remain on stable medications throughout the study. All participants were capable of providing written informed consent.

Inclusion criteria specifically included:

1. At least 22 years of age and no older than 75 years at the time of enrollment
2. Non-progressive cervical SCI from C2–C8 inclusive
3. AIS classification B, C or D
4. Indicated for upper extremity training procedures by the participantʼs treating physician, occupational therapist or physical therapist
5. GRASSP-Prehension score ≥10 or GRASSP-Strength score ≥30
6. Minimum 12 months after injury
7. If prescribed anti-spasticity or pain medications, must be at stable dose for at least 4 weeks before commencing study procedures
8. Capable of providing informed consent

Exclusion criteria specifically included:

1. Has uncontrolled cardiopulmonary disease or cardiac symptoms as determined by the investigator
2. Has any unstable or significant medical condition that is likely to interfere with study procedures or likely to confound study endpoint evaluations, such as severe neuropathic pain, depression, mood disorders or other cognitive disorders
3. Has been diagnosed with autonomic dysreflexia that is severe, unstable and uncontrolled
4. Requires ventilator support
5. Has an autoimmune etiology of spinal cord dysfunction/injury
6. History of additional neurologic disease, such as stroke, multiple sclerosis and traumatic brain injury

### Trial design

The Up-LIFT trial was designed by the sponsor (ONWARD Medical) and the investigators as a prospective, single-arm, sequential treatment, multicenter, open-label, non-significant risk trial to evaluate the safety and efficacy of ARC^EX^ Therapy to improve the recovery of arm and hand functions in people with chronic cervical SCI. This design enabled participants to serve as their own controls, which is the most appropriate design to control for the large variation among participants in baseline impairment, potential for responsiveness and optimal stimulation dose. As an added benefit, this design enabled a comparison of the influence of ARC^EX^ augmented rehabilitation to rehabilitation alone within the same participants. There was no concurrent control group. This trial design has a long history of application in pivotal multicenter trials involving neuromodulation therapies^52–56^ and was established after extensive interactions with the FDA that involved careful consideration of alternative designs, including randomized control designs and cross-over designs. The full pre-registered clinical protocol is provided in the Supplementary Information.

The results of this prospective, single-arm, sequential treatment, multicenter, open-label, non-significant risk trial represent the collective efforts from 14 neurorehabilitation centers in North America and Europe that are led by clinicians and researchers with extensive experience in rehabilitation medicine for people with SCI. All sites received training on all protocols, which were standardized across the centers.

All participants enrolled in the clinical trial underwent an intensive, standardized in-clinic rehabilitation program^44^ over a period of 2 months (Fig. 2). After this period, participants continued the same rehabilitation program with the addition of ARC^EX^ Therapy for two additional months. ARC^EX^ Therapy was applied during the entire session of rehabilitation to facilitate movement. Throughout the study, participants completed a minimum of 12 and a maximum of 20 in-clinic rehabilitation sessions per month. All performance metrics were assessed at enrollment and every month until completion of the study (Fig. 2). All assessments were performed in the absence of stimulation and are detailed in the protocol available online.

ARC^EX^ Therapy was delivered with a research version of the ARC^EX^ device, termed the LIFT device. The device includes two surface electrodes that were positioned in between the vertebral processes located generally one vertebral segment rostral and one vertebral segment caudal to the site of injury. Two large return electrodes were positioned over the iliac crests or clavicles. Stimulation was delivered at 30 Hz with a 10-kHz carrier frequency overlay, which consisted of 10 pulses with a 10-kHz frequency and 100-µs pulse width^65^ (Fig. 1a and Extended Data Fig. 1). The amplitude of stimulation was configured based on motor thresholds, absence of induced movements and patient comfort (Supplementary Information). This principle resulted in a broad distribution of stimulation amplitudes that was expected based on the diversity of body habitus of the participants enrolled in the study (Extended Data Fig. 1). All stimulation parameters and electrode locations are reported in Extended Data Fig. 1.

### Endpoints

The primary effectiveness endpoint tested the hypothesis that most of the participants would demonstrate significant improvements in both strength and functional performance domains from the end of the rehabilitation-alone period to the end of the ARC^EX^ Therapy period. Participants were considered responders if they met MID criteria determined with Cohen’s effect size method^66^ for at least one outcome in each of the strength and functional performance domains. Outcomes related to the strength domain included the ISNCSCI-UEMS^67^ (MID = 2-point improvement), the GRASSP-Strength score^64^ (MID = 4-point improvement), pinch force (MID = greater than or equal to 2.4-N improvement) and grasp force (MID = greater than or equal to 6-N improvement). Outcomes related to the functional domain included the GRASSP-Prehension Performance score^64^ (MID = 2-point improvement) and the CUE-T^68^ (MID = 4-point improvement). The primary safety endpoint for the Up-LIFT trial was the incidence of serious adverse events related to the use of ARC^EX^ Therapy.

Secondary effectiveness endpoints included the superiority of responder rates after completion of ARC^EX^ Therapy compared to during the rehabilitation-alone period as well as changes in single outcomes between enrollment and the end of the rehabilitation-alone period compared to between enrollment and the end of the ARC^EX^ Therapy period. These secondary effectiveness endpoints were hierarchically ordered a priori in the following sequence: pinch force, GRASSP-Prehension Performance score^64^, GRASSP-Strength score^64^, ISNCSCI-UEMS^67^, ISNCSCI TSS^67^, EQ-5D-5L score^69^, SCIM III^70^ and WHOQOL-BREF score^71^. The secondary safety endpoint was the incidence of all adverse events and serious adverse events in the trial.

Exploratory endpoints included additional outcomes that measured changes in the quality of life and the long-term consequences of SCI. These outcomes included the NRS for pain, the International Spinal Cord Injury Pain Data Set (ISCIPDS)^72^, the MOS Sleep Scale^73^, a subset of scores within the SCIM III^70^, the GRASSP-Sensibility score^64^, the PSFS^74^, subset scores within the EQ-5D-5L^69^ and the WHOQOL-BREF^71^, the International Standards to document remaining Autonomic Function after Spinal Cord Injury (ISAFSCI)^75^, the Patient Health Questionnaire-9 (PHQ-9)^76^ and the Global Impression of Change (Clinician and Patient)^77^. The functional profile of responders versus non-responders was also explored.

### Statistical analyses

A statistical plan was discussed and agreed upon with the FDA. The pre-registered statistical plan is provided in the Supplementary Information. A sample of 65 participants was calculated assuming a minimum power of 80%, a two-sided type I error of 10%, a responder rate of 67%, a performance goal of 50% and a 25% drop-out rate. All effectiveness endpoints were assessed within pre-specified modified intention-to-treat populations, wherein only participants who underwent at least 24 sessions (average of 12 sessions per month) during the rehabilitation-alone period and at least 24 sessions during the ARC^EX^ Therapy period were included in the analysis. This minimum number of exposures to both interventions was required to perform comparisons between the outcomes of the rehabilitation-alone and ARC^EX^ Therapy periods. All participants exposed to a study procedure were included in the safety analysis population.

The primary effectiveness endpoint was evaluated using a one-sided exact binomial test to address the hypothesis that the proportion of responders exceeded 50%. Secondary effectiveness endpoints assessed the superiority of improvements after ARC^EX^ Therapy when compared to rehabilitation alone. The superiority of responder rates in response to ARC^EX^ Therapy compared to rehabilitation alone was assessed using McNemar’s test. Identified secondary effectiveness outcomes were then assessed in hierarchical descending order, whereby downstream hypotheses were considered non-significant as soon as an endpoint was not met. Each secondary effectiveness outcome was tested with a paired one-sided *t*-test or a Wilcoxon signed-rank test, as appropriate, with a type I error rate of 5%. Across each secondary effectiveness outcome within the hierarchy, the change from enrollment to rehabilitation alone was compared to the change from enrollment to completion of ARC^EX^ Therapy. Descriptive statistics on additional outcomes in the primary and secondary endpoints were also conducted.

Exploratory endpoints were tested with a paired one-sided *t*-test or a Wilcoxon signed-rank test, as appropriate, with a type I error rate of 5%. Additional post hoc analyses included the identification of initial baseline characteristics that best predicted responder status as well as time-course effects for each of the primary and secondary outcome measures, tested with a repeated-measures ANOVA and post hoc testing using Tukeyʼs honest significant difference (HSD) method and a mixed model analysis of box and block scores, comparing rehabilitation alone to ARC^EX^ Therapy. The former analysis included sequential logistic regression models, whereby participants were binarized into two groups: above or below a single numerical threshold^78,79^. Odds ratios were then calculated, which reflected the odds of being a responder based on sequential thresholds for each outcome measure tested in the primary and secondary endpoints. The sequential models were halted when the odds ratio crossed 1, indicating a threshold above which participants demonstrated a positive likelihood of responding to ARC^EX^ Therapy. The latter analysis was completed as a one-way ANOVA with Tukeyʼs HSD post hoc testing for each outcome measure. Analyses were performed with SAS software, version 9.4 (SAS Institute). Details are provided in the Supplementary Information.

### Reporting summary

Further information on research design is available in the Nature Portfolio Reporting Summary linked to this article.

## Online content

Any methods, additional references, Nature Portfolio reporting summaries, source data, extended data, supplementary information, acknowledgements, peer review information; details of author contributions and competing interests; and statements of data and code availability are available at 10.1038/s41591-024-02940-9.

### Supplementary information

Supplementary Information Trial oversight, additional methodological details, extended data tables and extended data figure legends.

Reporting Summary

Supplementary Data 1 Individual values of each outcome measure in each participant.

Supplementary Data 2 Adverse events.

Supplementary Data Pre-registered clinical protocol.

Supplementary Data Pre-registered statistical analysis plan.

Supplementary Data 3 Statistics accompanying Fig. 3 and Extended Data Figs. 5 and 6.

## Figures and Tables

**Extended Data Table 1:**
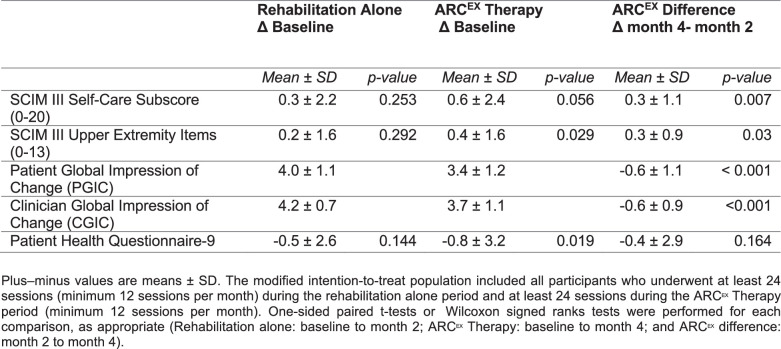
Patient-reported surveys related to quality of life, activities of daily living and impressions of change (modified intention-to-treat population)

**Extended Data Table 2:**
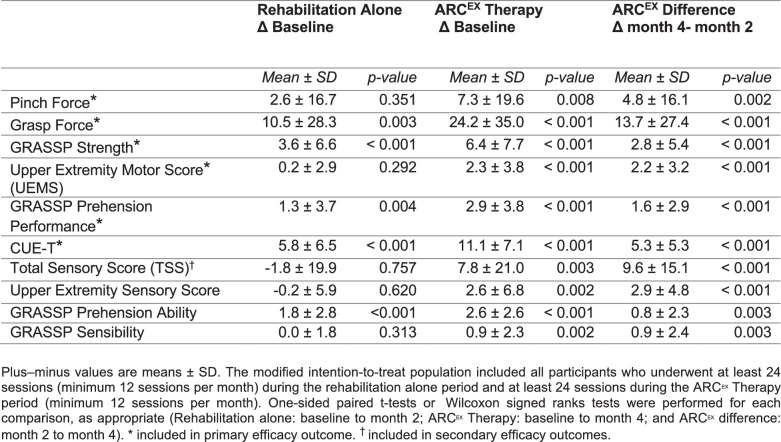
Additional domains within primary and secondary endpoints (modified intention-to-treat population)

**Extended Data Table 3:**
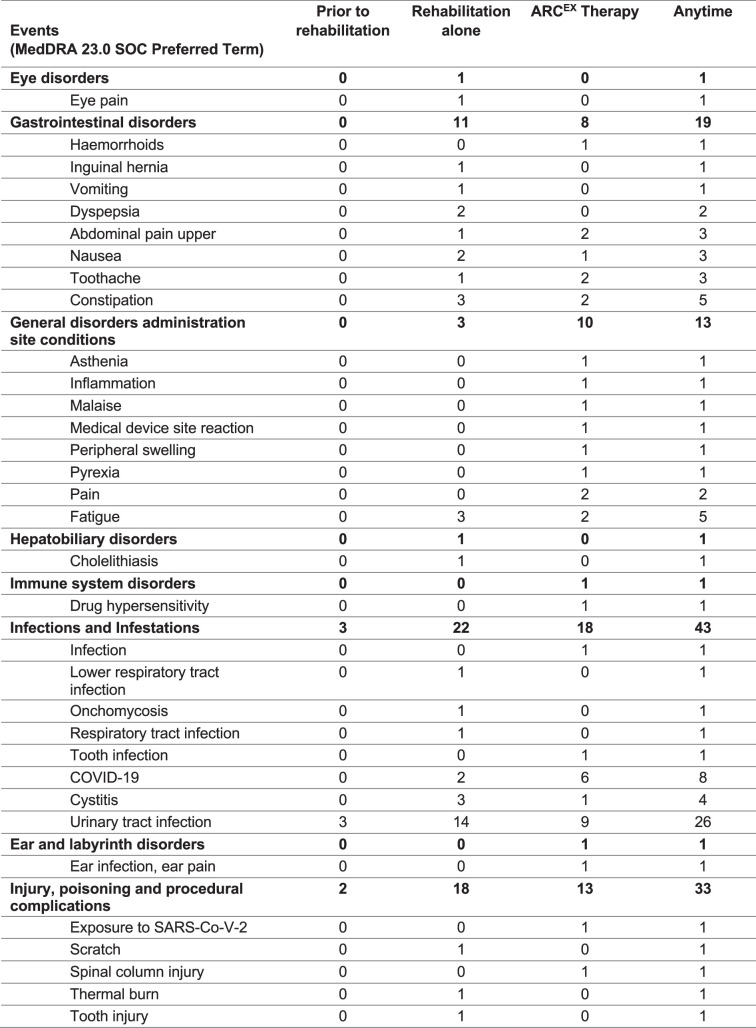

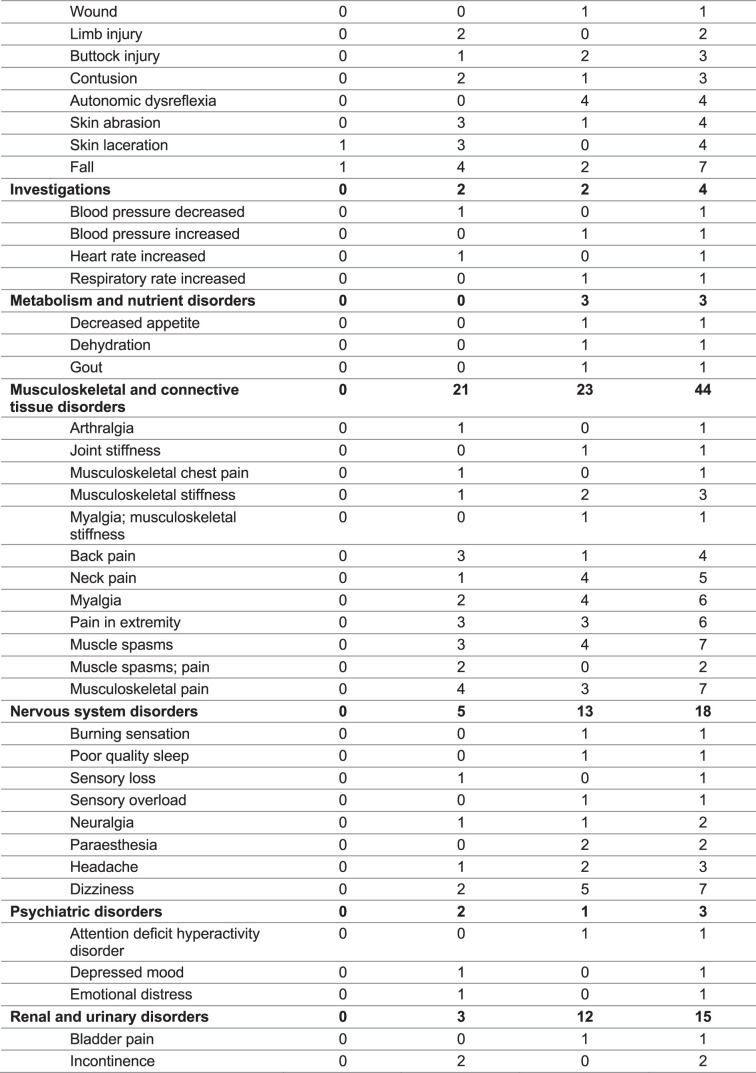

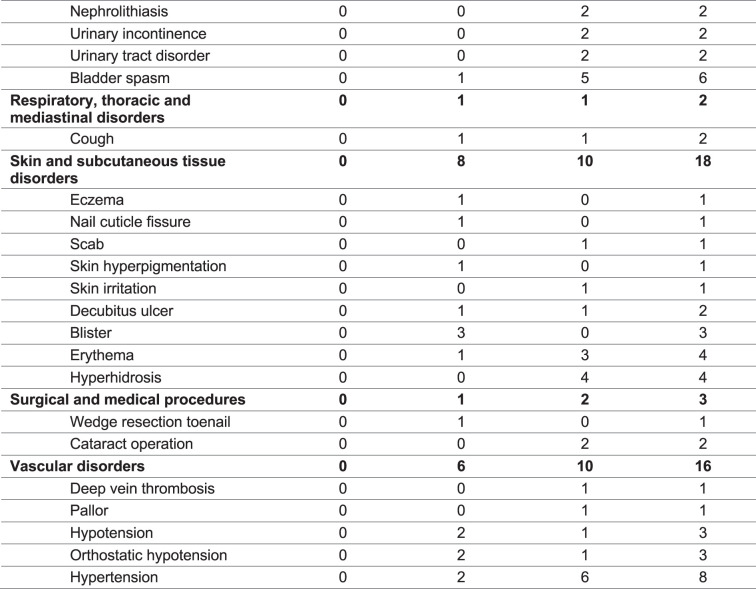
Detailed listing of adverse events (safety population)^*^

**Extended Data Table 4:**
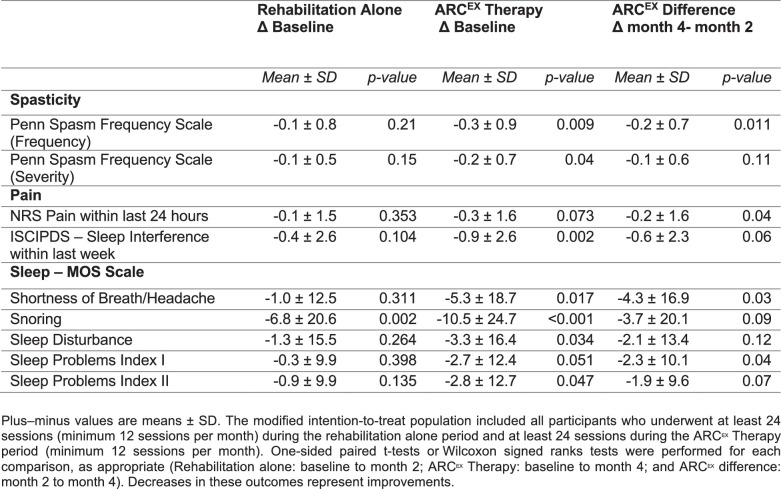
Spinal cord injury sequelae (modified intention-to-treat population)

## References

[1] Anderson, M. A. et al. Natural and targeted circuit reorganization after spinal cord injury. *Nat. Neurosci*. **25**, 1584–1596 (2022). 10.1038/s41593-022-01196-1 36396975

[2] Courtine G, Sofroniew MV (2019). Spinal cord repair: advances in biology and technology. Nat. Med..

[3] Wilson J, Hashimoto R, Dettori J, Fehlings M (2011). Spinal cord injury and quality of life: a systematic review of outcome measures. Evid. Based Spine Care.

[4] Kokotilo KJ, Eng JJ, Curt A (2009). Reorganization and preservation of motor control of the brain in spinal cord injury: a systematic review. J. Neurotrauma.

[5] Gomes-Osman J, Cortes M, Guest J, Pascual-Leone A (2016). A systematic review of experimental strategies aimed at improving motor function after acute and chronic. J. Neurotrauma.

[6] Munce SEP (2013). Impact of quality improvement strategies on the quality of life and well-being of individuals with spinal cord injury: a systematic review protocol. Syst. Rev..

[7] Asboth L (2018). Cortico-reticulo-spinal circuit reorganization enables functional recovery after severe spinal cord contusion. Nat. Neurosci..

[8] Kathe, C. et al. The neurons that restore walking after paralysis. *Nature* **611**, 540–547 (2022). 10.1038/s41586-022-05385-7 PMC9668750 36352232

[9] Wenger N (2016). Spatiotemporal neuromodulation therapies engaging muscle synergies improve motor control after spinal cord injury. Nat. Med..

[10] van den Brand R (2012). Restoring voluntary control of locomotion after paralyzing spinal cord injury. Science.

[11] Capogrosso M (2016). A brain–spine interface alleviating gait deficits after spinal cord injury in primates. Nature.

[12] Squair JW (2021). Neuroprosthetic baroreflex controls haemodynamics after spinal cord injury. Nature.

[13] Lavrov I (2008). Epidural stimulation induced modulation of spinal locomotor networks in adult spinal rats. J. Neurosci..

[14] Squair JW (2022). Implanted system for orthostatic hypotension in multiple-system atrophy. N. Engl. J. Med..

[15] Alam M (2017). Electrical neuromodulation of the cervical spinal cord facilitates forelimb skilled function recovery in spinal cord injured rats. Exp. Neurol..

[16] Shah P (2016). Unique spatiotemporal neuromodulation of the lumbosacral circuitry shapes locomotor success after spinal cord injury. J. Neurotrauma.

[17] Barra, B. et al. Epidural electrical stimulation of the cervical dorsal roots restores voluntary upper limb control in paralyzed monkeys. *Nat. Neurosci.* **25**, 924–934 (2022). 10.1038/s41593-022-01106-5 35773543

[18] Harkema S (2011). Effect of epidural stimulation of the lumbosacral spinal cord on voluntary movement, standing, and assisted stepping after motor complete paraplegia: a case study. Lancet.

[19] Harkema SJ (2018). Epidural spinal cord stimulation training and sustained recovery of cardiovascular function in individuals with chronic cervical spinal cord injury. JAMA Neurol..

[20] Angeli CA (2018). Recovery of over-ground walking after chronic motor complete spinal cord injury. N. Engl. J. Med..

[21] Gill ML (2018). Neuromodulation of lumbosacral spinal networks enables independent stepping after complete paraplegia. Nat. Med..

[22] Darrow D (2019). Epidural spinal cord stimulation facilitates immediate restoration of dormant motor and autonomic supraspinal pathways after chronic neurologically complete spinal cord injury. J. Neurotrauma.

[23] Rowald A (2022). Activity-dependent spinal cord neuromodulation rapidly restores trunk and leg motor functions after complete paralysis. Nat. Med..

[24] Wagner FB (2018). Targeted neurotechnology restores walking in humans with spinal cord injury. Nature.

[25] Hofstoetter US (2014). Modification of spasticity by transcutaneous spinal cord stimulation in individuals with incomplete spinal cord injury. J. Spinal Cord Med..

[26] Hofstoetter US (2020). Transcutaneous spinal cord stimulation induces temporary attenuation of spasticity in individuals with spinal cord injury. J. Neurotrauma.

[27] Phillips AA (2018). An autonomic neuroprosthesis: noninvasive electrical spinal cord stimulation restores autonomic cardiovascular function in individuals with spinal cord injury. J. Neurotrauma.

[28] Kreydin E (2020). Transcutaneous electrical spinal cord neuromodulator (TESCoN) improves symptoms of overactive bladder. Front. Syst. Neurosci..

[29] Gad PN, Kreydin E, Zhong H, Latack K, Edgerton VR (2018). Non-invasive neuromodulation of spinal cord restores lower urinary tract function after paralysis. Front. Neurosci..

[30] Herrity AN, Williams CS, Angeli CA, Harkema SJ, Hubscher CH (2018). Lumbosacral spinal cord epidural stimulation improves voiding function after human spinal cord injury. Sci. Rep..

[31] Lu DC (2016). Engaging cervical spinal cord networks to reenable volitional control of hand function in tetraplegic patients. Neurorehabil. Neural Repair.

[32] Gad P (2018). Non-invasive activation of cervical spinal networks after severe paralysis. J. Neurotrauma.

[33] Inanici, F., Brighton, L. N., Samejima, S., Hofstetter, C. P. & Moritz, C. T. Transcutaneous spinal cord stimulation restores hand and arm function after spinal cord injury. *IEEE Trans. Neural Syst. Rehabil. Eng.* **29**, 310–319 (2021). 10.1109/TNSRE.2021.3049133 33400652

[34] Inanici F (2018). Transcutaneous electrical spinal stimulation promotes long-term recovery of upper extremity function in chronic tetraplegia. IEEE Trans. Neural Syst. Rehabil. Eng..

[35] Sharma P (2023). Multi-site spinal cord transcutaneous stimulation facilitates upper limb sensory and motor recovery in severe cervical spinal cord injury: a case study. J. Clin. Med..

[36] Benavides FD (2020). Cortical and subcortical effects of transcutaneous spinal cord stimulation in humans with tetraplegia. J. Neurosci..

[37] Rejc E, Angeli CA, Atkinson D, Harkema SJ (2017). Motor recovery after activity-based training with spinal cord epidural stimulation in a chronic motor complete paraplegic. Sci. Rep..

[38] Powell, M. P. et al. Epidural stimulation of the cervical spinal cord for post-stroke upper-limb paresis. *Nat. Med.* **29**, 689–699 (2023). 10.1038/s41591-022-02202-6 PMC10291889 36807682

[39] Hoffman LR, Field-Fote EC (2010). Functional and corticomotor changes in individuals with tetraplegia following unimanual or bimanual massed practice training with somatosensory stimulation: a pilot study. J. Neurol. Phys. Ther..

[40] Kumru H (2021). Transcutaneous electrical neuromodulation of the cervical spinal cord depends both on the stimulation intensity and the degree of voluntary activity for training. a pilot study. J. Clin. Med..

[41] Jo HJ, Perez MA (2020). Corticospinal-motor neuronal plasticity promotes exercise-mediated recovery in humans with spinal cord injury. Brain.

[42] Courtine G, Harkema SJ, Dy CJ, Gerasimenko YP, Dyhre-Poulsen P (2007). Modulation of multisegmental monosynaptic responses in a variety of leg muscles during walking and running in humans. J. Physiol..

[43] National Spinal Cord Injury Statistical Center. Traumatic Spinal Cord Injury Facts and Figures at a Glance. https://msktc.org/sites/default/files/SCI-Facts-Figs-2022-Eng-508.pdf (2022).

[44] Gomes-Osman J, Tibbett JA, Poe BP, Field-Fote EC (2017). Priming for improved hand strength in persons with chronic tetraplegia: a comparison of priming-augmented functional task practice, priming alone, and conventional exercise training. Front. Neurol..

[45] Hoffman L, Field-Fote E (2013). Effects of practice combined with somatosensory or motor stimulation on hand function in persons with spinal cord injury. Top. Spinal Cord. Inj. Rehabil..

[46] Beekhuizen KS, Field-Fote EC (2008). Sensory stimulation augments the effects of massed practice training in persons with tetraplegia. Arch. Phys. Med. Rehabil..

[47] Diong J (2012). Incidence and predictors of contracture after spinal cord injury—a prospective cohort study. Spinal Cord.

[48] Garcia-Arguello LY (2017). Infections in the spinal cord-injured population: a systematic review. Spinal Cord.

[49] Waters RL, Adkins RH, Yakura JS, Sie I (1994). Motor and sensory recovery following incomplete tetraplegia. Arch. Phys. Med. Rehabil..

[50] Mateo S, Marco JD, Cucherat M, Gueyffier F, Rode G (2020). Inconclusive efficacy of intervention on upper-limb function after tetraplegia: a systematic review and meta-analysis. Ann. Phys. Rehabil. Med..

[51] Kalsi-Ryan S (2014). Outcome of the upper limb in cervical spinal cord injury: profiles of recovery and insights for clinical studies. J. Spinal Cord Med..

[52] Strollo PJ (2014). Upper-airway stimulation for obstructive sleep apnea. N. Engl. J. Med..

[53] Anand A (2023). Ketamine versus ECT for nonpsychotic treatment-resistant major depression. N. Engl. J. Med..

[54] Pluymaekers NAHA (2019). Early or delayed cardioversion in recent-onset atrial fibrillation. N. Engl. J. Med..

[55] Blumberger DM (2018). Effectiveness of theta burst versus high-frequency repetitive transcranial magnetic stimulation in patients with depression (THREE-D): a randomised non-inferiority trial. Lancet.

[56] Amundsen CL (2016). OnabotulinumtoxinA vs sacral neuromodulation on refractory urgency urinary incontinence in women: a randomized clinical trial. JAMA.

[57] Martínez-Fernández R (2020). Randomized trial of focused ultrasound subthalamotomy for Parkinson’s disease. N. Engl. J. Med..

[58] Kupsch A (2006). Pallidal deep-brain stimulation in primary generalized or segmental dystonia. N. Engl. J. Med..

[59] Mallet L (2008). Subthalamic nucleus stimulation in severe obsessive–compulsive disorder. N. Engl. J. Med..

[60] Rowald, A. et al. Recovery of trunk and leg motor functions within one day after chronic complete paralysis. *Nat. Med.* (in the press).

[61] Lammertse D (2007). Guidelines for the conduct of clinical trials for spinal cord injury as developed by the ICCP panel: clinical trial design. Spinal Cord.

[62] Milekovic, T. et al. A spinal cord neuroprosthesis for locomotor deficits due to Parkinson’s disease. *Nat. Med*. **29**, 2854–2865 (2023). 10.1038/s41591-023-02584-1 37932548

[63] Kirshblum S, Waring W (2014). Updates for the International Standards for Neurological Classification of Spinal Cord Injury. Phys. Med. Rehabil. Clin. North Am..

[64] Kalsi-Ryan S (2012). The graded redefined assessment of strength sensibility and prehension: reliability and validity. J. Neurotrauma.

[65] Gerasimenko Y (2015). Transcutaneous electrical spinal-cord stimulation in humans. Ann. Phys. Rehabil. Med..

[66] Selya AS, Rose JS, Dierker LC, Hedeker D, Mermelstein RJ (2012). A practical guide to calculating Cohen’s *f*^2^, a measure of local effect size, from PROC MIXED. Front. Psychol..

[67] Kirshblum SC (2011). Reference for the 2011 revision of the International Standards for Neurological Classification of Spinal Cord Injury. J. Spinal Cord Med..

[68] Marino RJ, Kern SB, Leiby B, Schmidt-Read M, Mulcahey MJ (2015). Reliability and validity of the capabilities of upper extremity test (CUE-T) in subjects with chronic spinal cord injury. J. Spinal Cord Med..

[69] Herdman M (2011). Development and preliminary testing of the new five-level version of EQ-5D (EQ-5D-5L). Qual. Life Res..

[70] Catz A, Itzkovich M, Agranov E, Ring H, Tamir A (1997). SCIM—spinal cord independence measure: a new disability scale for patients with spinal cord lesions. Spinal Cord.

[71] Jang Y, Hsieh C-L, Wang Y-H, Wu Y-H (2004). A validity study of the WHOQOL-BREF assessment in persons with traumatic spinal cord injury. Arch. Phys. Med. Rehabil..

[72] Widerström-Noga E (2008). The international spinal cord injury pain basic data set. Spinal Cord.

[73] *Measuring Functioning and Well-Being: The Medical Outcomes Study Approach* (eds Stewart, A. & Ware, J.) (RAND, 1992); 10.7249/CB361

[74] Mills PB, Vakil AP, Phillips C, Kei L, Kwon BK (2018). Intra-rater and inter-rater reliability of the Penn Spasm Frequency Scale in people with chronic traumatic spinal cord injury. Spinal Cord.

[75] Contributors (2012). International standards to document remaining autonomic function after spinal cord injury. J. Spinal Cord Med..

[76] Kroenke K, Spitzer RL, Williams JBW (2001). The PHQ-9: validity of a brief depression severity measure. J. Gen. Intern. Med..

[77] Kamper SJ, Maher CG, Mackay G (2009). Global rating of change scales: a review of strengths and weaknesses and considerations for design. J. Man. Manip. Ther..

[78] Squair JW (2019). Empirical targets for acute hemodynamic management of individuals with spinal cord injury. Neurology.

[79] Squair JW (2017). Spinal cord perfusion pressure predicts neurologic recovery in acute spinal cord injury. Neurology.

