# Supplementary material for: Non-invasive spinal cord electrical stimulation for arm and hand function in chronic tetraplegia: a safety and efficacy trial
Source: Nat Med. 2024 May 20;30(5):1276–83. doi: 10.1038/s41591-024-02940-9 (PMC11108781; doi:10.1038/s41591-024-02940-9)
Supplement: Supplementary file 1 — Trial oversight, additional methodological details, extended data tables and extended data figure legends. [file 41591_2024_2940_MOESM1_ESM.pdf]

nature medicine

Article

https://doi.org/10.1038/s41591-024-02940-9

Non-invasive spinal cord electrical stimulation for arm and hand function in chronic tetraplegia: a safety and efficacy trial

In the format provided by the authors and unedited

Supplementary Information

1. **Trial Oversight**
    1.1. Study investigators and institutions ....**p 1**
    1.2. Data Safety Monitoring Board ....**p 1**
    1.3. Trial Registration ....**p 1**
    1.4. Contributors ....**p 1**
2. **Additional Methodological Details**
    2.1. Participant recruitment / inclusion / exclusion...**p 1**
    2.2. Stimulation protocol..**p 2**
    2.3. Rehabilitation protocol..**p 2**
    2.4. Descriptions of the study assessments..**p 2**
        2.4.1. International Standard Neurological Classification of Spinal Cord Injury (ISNCSCI) ....**p 3**
        2.4.2. Graded Refined Assessment of Strength, Sensibility and Prehension (GRASSP) ....**p 3**
        2.4.3. Pinch and Grasp Force ....**p 4**
        2.4.4. Capabilities of Upper Extremity Test (CUE-T) ....**p 4**
        2.4.5. Numerical Rating Scale for Pain ....**p 4**
        2.4.6. International Spinal Cord Injury Pain Data Set (ISCIPDS) ....**p 4**
        2.4.7. Medical Outcomes Study (MOS) Sleep Score ....**p 4**
        2.4.8. Spinal Cord Independence Measure (SCIM III) ....**p 5**
        2.4.9. Penn Spasm Frequency Scale (PSFS) ....**p 5**
        2.4.10. 5-Dimension, 5-Level European Quality of Life (EQ-5D-5L) ....**p 5**
        2.4.11. World Health Organization Quality of Life Measure (WHOQOL-BREF) ....**p 5**
        2.4.12. International Standards to document remaining Autonomic Function after SCI (ISAFSCI) ……...**p 5**
        2.4.13. 9-Item Patient Health Questionnaire (PHQ-9) ……………….....................................**p 5**
        2.4.14. Patient/Clinician Global Impression of Change (PGIC/CGIC) ……………...................**p 5**
    2.5. Definition of Minimal Important Different (MID) ....**p 5**
3. **Extended Data Tables**
    - Extended Data Table 1. Additional Domains within Primary and Secondary End points ……….............**p 11**
    - Extended Data Table 2. Patient-Reported Surveys Related to Quality of Life, Activities of Daily Living and Impressions of Change...**p 11**
    - Extended Data Table 3. Spinal Cord Injury Sequelae ....**p 12**
    - Extended Data Table 4. Detailed Listing of Adverse Events (Safety Population) ....**p 13**
4. **Extended Data Figure Legends** ……………………………………………………..……………….**p 16**

1. Trial Oversight

1.1. Study investigators and institutions

1. University of Washington Medical Center Montlake (Dr. Chet Moritz, PhD)
2. Craig Hospital (Dr. Candace Tefertiller, DPT, PhD)
3. University Health Network -Toronto (Dr. Sukhvinder Kalsi-Ryan, BScPT, MSc, PhD)
4. Spaulding Rehabilitation Hospital (Dr. Randy Trumbower, PT, PhD)
5. University of Minnesota -Boynton Health (Dr. Leslie Morse, DO)
6. Shepherd Center (Dr. Edelle Field-Fote, PT, PhD)
7. Thomas Jefferson University (Dr. Ralph Marino, MD)
8. Mayo Clinic (Dr. Kristin Zhao, PhD)
9. Intl Collaboration on Repair Discoveries (ICORD) (Dr. Andrei Krassioukov, MD, PhD)
10. Bronx Veterans Medical Research Foundation, Inc. (Dr. Linda Murray, PhD)
11. University of Miami, Miller School of Medicine (Dr. James Guest, MD, PhD, FAANS)
12. Reade (Dr. Thomas Janssen, PhD)
13. St. Maartensklinek (Dr. Ilse van Nes, MD, PhD)
14. Queen Elizabeth (Dr. Margaret Purcell, MD)

1.2. Data Safety Monitoring Board

1. Dr Mark S. Nash (Ph.D., FACSM) (Chair of the DSMB)
   Professor, Departments of Neurological Surgery and Physical Medicine & Rehabilitation
   Associate Scientific Director for Research, The Miami Project to Cure Paralysis, Vice-Chair for Research, Department of Physical Medicine and Rehabilitation, Miller School of Medicine, University of Miami, FL
2. Dr. Noam Harel (MD, PhD)
   Associate Professor Neurology; Rehabilitation and Human Performance Icahn School of Medicine at Mt Sinai, NY
3. Dr. Graham Creasey (MB, ChB, FRCS Ed)
   Paralyzed Veterans of America Professor of Spinal Cord Injury Medicine (Emeritus) Neurosurgery Stanford University, CA

1.3. Trial Registration

Participants were enrolled (consented for screening) in the UpLift trial (NCT04697472) between January to December, 2021, and the trial was registered on https://clinicaltrials.gov/ on January 6, 2021.

1.4. Contributors

CM, EF, CT, JMD, GC, JWS, FA, contributed to the analyses and/or design of the study.
All the Study investigators contributed to acquisition or interpretation of the data.
GC and JWS wrote the manuscript in collaboration with all the authors and sponsor.

2. Additional Methodological Details

2.1. Participant recruitment / inclusion / exclusion

For inclusion in the trial, participants were required to fulfill all the following criteria: (i) At least 22 years old and no older than 75 years old at the time of enrollment, (ii) Non-progressive cervical spinal cord injury from C2-C8 inclusive, (iii) American Spinal Injury Association (ASIA) Impairment Scale (AIS) classification B, C,

or D, (iv) Indicated for upper extremity training procedures by subject's treating physician, occupational therapist, or physical therapist, (v) GRASSP-Prehension Performance score ≥ 10 or GRASSP-Strength score ≥ 30, (vi) Minimum 12 months post-injury. (vii) If prescribed anti-spasticity or pain medications, must be at stable dose for at least 4 weeks prior to commencing study procedures, (viii) Capable of providing informed consent. Any of the following was regarded as a criterion for exclusion from the trial: (i) Has uncontrolled cardiopulmonary disease or cardiac symptoms as determined by the Investigator, (ii) Has any unstable or significant medical condition that is likely to interfere with study procedures or likely to confound study end point evaluations like severe neuropathic pain, depression, mood disorders or other cognitive disorders, (iii) Has been diagnosed with autonomic dysreflexia that is severe, unstable, and uncontrolled, (iv) Requires ventilator support, (v) Has an autoimmune etiology of spinal cord dysfunction/injury, (vi) History of additional neurologic disease such as stroke, multiple sclerosis, traumatic brain injury, etc., (vii) Peripheral neuropathy (diabetic polyneuropathy, entrapment neuropathy, etc.), (vii) Spasms that limit the ability of the subjects to participate in the study training as determined by the Investigator, (viii) Received Botulinum toxin injections in their upper extremity, neck, or hand within 6 months prior to enrollment, (ix) Has urinary tract infection or any of the following issues in the upper extremity or the cervical spine region at the time of enrollment: painful musculoskeletal dysfunction unrelated to SCI, unhealed fracture, contracture, pressure sore, (x) Breakdown in skin area that will come into contact with electrodes, (xi) Presence of syringomyelia as confirmed by an MRI, (xii) Currently undergoing treatment for cancer or has been in remission for less than 2 years, (xiii) Received stem cell treatment within the past two years prior to enrollment, (xiv) Prior nerve or tendon transfer procedure in the upper extremities (xv) Total baclofen dose >30 mg per day, (xvi) Has any active implanted medical device, (xvii) Pregnant, planning to become pregnant or currently breastfeeding, (xviii) Concurrent participation in another drug or device trial that may interfere with this study, (xix) Has undergone a prior course of spinal stimulation therapy directed at UE improvement, (xx) In the opinion of the investigators, the study is not safe or appropriate for the participant and/or the participant is unlikely to return for the follow-up visits per the protocol.

2.2. Stimulation protocol

The stimulation protocol in this study was adapted from previously described protocols [33]. Subjects underwent neurophysiologic assessments with the LIFT System to assess whether their neurological status was compatible with the ARC^{EX} Therapy, and to identify the intensity of the stimulation that was necessary to reach the threshold to elicit muscle responses. This threshold guided the selection of the intensity of the stimulation. Stimulation was delivered below the identified threshold to elicit muscle responses, which was defined as a subthreshold stimulation intensity. The intensity of the stimulation was adjusted to maximize task performance during rehabilitation. The stimulation waveforms were configured as monophasic or biphasic (**Figure 1**) based on the configuration that mediated the most robust facilitation of arm and hand movements. The intensity of the stimulation was increased gradually (e.g., 5 mA steps) until the increase in muscle tone began to interfere with movement coordination or was judged uncomfortable by the participant. Treatment was performed with amplitudes of stimulation coinciding approximately with the motor threshold, and adjusted as needed for the remainder of the ARC^{EX} Therapy sessions. The specific stimulation parameters and optimal stimulation intensity were left to the discretion of the investigator and research team. A variety of electrode configurations and output settings were used without any safety concerns. In general, a burst frequency of 30 Hz, a carrier frequency of 10 kHz, and a pulse width of 100μs was used throughout. Varying parameters including electrode placement, stimulation mode, and amplitude range are reported in **Figure S1**.

2.3. Rehabilitation protocol

To ensure homogeneity across sites, a stereotypical rehabilitation program was defined [44]. This program involved 1 to 2 hours of training, 3-5 times per week covering task-specific categories including pinch, grasp,

grasp with rotation, and whole arm movement. The exact content of the rehabilitation program for each participant was determined by the study investigator in consultation with other research personnel at each site and tuned to the capabilities of each individual participant. The rehabilitation program consisted of functional tasks that included repetitive activities of gross upper extremity movement, isolated finger movements, simple and complex pinch, and grasping activities. For each category, 4-10 occupational therapy activities with various difficulty levels were identified. Each participant performed at least 1-2 exercises within the same category during each treatment session. Activities in each category were chosen according to the participants' ability. These activities were adjusted based on the evolution of the ability to perform the functional task over time (graded training). Typical movement patterns were encouraged by guidance and feedback. Compensatory movements, such as wrist tenodesis, were strongly discouraged during therapy delivery. When a participant had little to no voluntary movement, active assistance from a physical or occupational therapist, or qualified clinical team member, was provided to complete the desired activity. At the end of each session, improvements in upper extremity function and strength gained from the exercise training were assessed for each participant through a box and block test.

In total, the participants underwent rehabilitation alone for 2 months, participating in an average of 3 sessions/week for a total of 12-20 sessions/month, and a minimum number of 24 sessions of rehabilitation for the wash-in phase.

2.4. ARC^{EX} Therapy

Following 2 months of rehabilitation alone, participants began the ARC^{EX} Therapy phase of the trial. Participants followed the same rehabilitation protocol as described in Section 2.2 with the addition of externally applied electrical stimulation of the cervical spinal cord that was delivered continuously throughout the session using the LIFT device. These sessions involved 1-2 hours of rehabilitative training that were performed 3 to 5 times per week. In total, participants completed 12 to 20 sessions per month, ensuring that each participant performed at least 24 sessions of rehabilitation with ARC^{EX} Therapy.

2.5. Descriptions of the study assessments

2.5.1. International Standards for Neurological Classification of Spinal Cord Injury (ISNCSCI)

The International Standards for Neurological Classification of Spinal Cord Injury (ISNCSCI) including the ASIA Impairment Scale (AIS) [67], were developed and continuously maintained by the International Standards Committee of the American Spinal Injury Association (ASIA) and the International Spinal Cord Society (ISCoS) as a universal classification tool for Spinal Cord Injury based on a standardized sensory and motor assessment, with the most recent revised edition published in 2019 [67]. The impairment scale involves both a motor and sensory examination to determine the sensory and motor levels for the right and left side, the overall neurological level of the injury and completeness of the injury.

ISNCSCI motor and sensory scores are derived from 4 sub-scores: upper and lower extremities and left and right sides. Upper extremity and lower extremity motor scores (UEMS, LEMS) are derived from grading 5 muscles each in the upper and lower extremities on a scale of 0 (total paralysis) to 5 (normal active movement, full range of motion against gravity and sufficient resistance). Sensory scores are derived similarly for upper and lower extremities by grading pinprick and light touch sensation on a scale with 0 (sensation absent), 1 (present, abnormal) and 2 (present, normal).

2.5.2. Graded Refined Assessment of Strength, Sensibility and Prehension (GRASSP)

The GRASSP test is a clinical impairment measure specific for the assessment of arm and hand functions in people with tetraplegia [64]. GRASSP is a multimodal test that measures sensorimotor and prehension function in three domains that are important for the description of arm and hand functions, namely strength,

sensation and prehension. They comprise five subtests for each arm and hand (that is, the test was performed bilaterally): strength, dorsal sensation, palmar sensation, prehension ability and prehension performance. GRASSP Strength was assessed by testing for muscle contraction and range of motion with or without gravity and graded as 0 (no palpable muscle contraction) to 5 (full range of motion against gravity with maximum resistance) for 5 key muscles in each upper extremity (total score = 100). GRASSP Sensation (dorsal sensation and palmar sensation) was tested using Semmes-Weinstein 4 monofilament probes. The pressure applied and sensation elicited was represented by numeric values ranging from 0 (no response) to 4 (normal sensation) (total score = 24). GRASSP Prehension (prehension ability and performance) was divided into ability and performance. This domain captures the influence of sensation and strength on goal-oriented arm and hand tasks, such as cylindrical grasp and lateral pinch along with 6 timed tasks [64] (total score for GRASSP Prehension Ability = 24; GRASSP Prehension Performance = 40).

The GRASSP was developed with the intent to be a clinical and research tool that would (i) capture information on upper limb impairment from the population of people with tetraplegia, (ii) obtain integrated sensory and motor impairment data, and discriminate the population according to impairment and function, (iii) be responsive (sensitive) to change over time, (iv) assess the extent of spontaneous (natural) recovery, and (v) be applied in clinical settings and in clinical trials/studies to evaluate the effect of novel interventions.

2.5.3. Pinch and Grasp Force

Pinch and grasp force were measured by the Commander Echo Console with the Pinch and Hand Dynamometer (JTech Medical, Salt Lake City, UT) to quantify maximum finger and grasp strength in Newtons.

1.1.1. Capabilities of Upper Extremity Test (CUE-T)

CUE-T is an assessment tool that measures functional limitation and assesses the capacity to perform specific actions with one or both arms and hands in individuals with tetraplegia[68]. Items evaluate upper extremity actions such as reaching, lifting, pulling, and pushing in addition to various grasp patterns. Item scores range from 0 to 4; the total score ranges from 0 – 128 [68].

2.5.4. Numerical Rating Scale for Pain

This assessment was rated by the participant based on an anchored 0-10 rating for pain, whereby “0” scored the absence of any pain, while “10” denoted the worst pain imaginable. The recall period was adjusted based on how the question was phrased, e.g., in the last 7 days, rate your average pain on a scale ranging from 0 to 10. The score captures the average pain experienced by the participant during the recall period.

2.5.5. International Spinal Cord Injury Pain Data Set (ISCIPDS)

To standardize collection and reporting of pain in SCI, ISCIPDS was developed by an international consortium of pain and SCI experts [72]. It collects pain interference with day-to-day activities, mood, and sleep over a 7-day recall period. Interference and Worst Pain Problem(s) were scored on a scale from 0 (no interference) to 10 (extreme interference) with a recall period of 7 days.

2.5.6. Medical Outcomes Study (MOS) Sleep Score

The *MOS* Sleep Score was developed for the MOS, which was a two-year study conducted in people with chronic conditions. MOS-Sleep contains 12 self-rated questions [73]. Ten questions are ranked 1 to 6, and reflect sleep duration, sleep disturbance, adequacy, and somnolence. A question on the necessary duration of time to fall asleep, and a second question on how many hours are spent asleep each night are also included in the questionnaire. Scores ranged from 1 (all of the time) to 6 (none of the time) for each of the 10 self-rated questions examining sleep disturbance, snoring, shortness of breath/headache, sleep

adequacy, sleep somnolence and sleep problem indices I and II. Sleep quantity is reported as hours of sleep and a ranking of 1-6 for time to fall asleep is collected. Scores are coded as per the instructions and multiple outcomes are reported that reflect different aspects of sleep quality [73].

2.5.7. *Spinal Cord Independence Measure (SCIM III)*

The SCIM has been developed to address three specific areas of function in subjects with SCI [70]. A subset of scores was examined that included: self-care (feeding, grooming, bathing, and dressing), respiration and sphincter management, and mobility (bed and transfers and indoors/outdoors). Scores were calculated as per the guidelines of the SCIM III [70].

2.5.8. *Penn Spasm Frequency Scale (PSFS)*

A two-component, self-reported measure of frequency and severity of spasms after SCI [74]. In this trial, the frequency (Scored 0-4; 0 = No spasms to 4 = Spontaneous spasms occurring more than ten times per hour) and severity (Scored 1-3; 1 = Mild, 3 = Severe) were examined.

2.5.9. *5-Dimension, 5-Level European Quality of Life (EQ-5D-5L)*

EQ-5D instrument comprises a short descriptive system questionnaire to assess the health state of an individual [69]. Domains included in this trial were mobility, self-care, usual activities, pain/discomfort, and anxiety/depression. The state of each domain is represented by number ranging from 0 through 4. The state of all domains can then be represented together as a 5-digt number (e.g., 42311), where each number represents an individual domain. This number is then translated into an index score. The questionnaire also has a visual analogue scale (EQ VAS) to capture the health state. Scores for VAS domains ranged from 0 (worst health imaginable) to 100 (best health imaginable). Overall EQ-5D-5L Index scores ranged from 0.000 – 1.000 [69].

2.5.10. *World Health Organization Quality of Life Measure (WHOQOL-BREF)*

WHOQOL-BREF contains 26 questions in 4 domains with 24 of them assigned to the facets/areas relevant to quality of life. Two questions address overall quality of life and general health. The domains examined in this trial included physical health, psychological, social relationships, environment. Scores were transformed to be between 4-20 or 0-100, as per the instructions of the WHOQOL-BREF [71].

2.5.11. *International Standards to document remaining Autonomic Function after Spinal Cord Injury (ISAFSCI)*

The ISAFSCI comprises a semi-quantitative questionnaire on autonomic control including heart function, blood pressure, sweating, temperature regulation, broncho-pulmonary system, bladder and bowel management and sexual function [75]. Scoring was completed according to the guidelines, which included: Normal/abnormal (heart function, blood pressure, sweating, temperature regulation, broncho-pulmonary system); 0 = Complete loss of control to 2 = Normal function (bladder and bowel management, sexual function).

2.5.12. *9-Item Patient Health Questionnaire (PHQ-9)*

The PHQ-9 represents a clinical-standard, nine-item, self-reported measure of depression. Scores range from 0 (Not at all) to 3 (Nearly every day) for each item, resulting in a score of 0-27 [76].

2.5.13. *Patient/Clinician Global Impression of Change (PGIC/CGIC)*

Global impression of change (GIC) is a 9-point Likert scale used to assess treatment-induced changes in a subject's clinical status (improvement or decline) [77]. The GIC provides a general indication of changes

related to activity limitations, symptoms, emotions, and overall quality of life. The questionnaire is completed both by the study subjects (Patient GIC) and the physicians (Clinician GIC) and may be used to validate the relative clinical benefit of treatment as quantified by other outcome measures used in the study.

2.6. Definition of MID

Since there is no relevant prior data on minimum detectable difference (MDD) or minimal clinically important difference (MCID) in the population of people with chronic SCI, an appropriate alternative methodology was used to determine the effect size (d), as described by Cohen [66] as follows:

$$d = (m1 - m2) / \sqrt{(\sigma1^2 + \sigma2^2) / 2}$$

Where, m1, m2 = Means of two independent samples; σ1, σ2= Standard deviations of the respective populations. Cohen's effect size is a measure of difference in means between the test and the control populations. Cohen's effect size measures the degree to which a phenomenon exists in the population or is induced by the treatment. It can also quantify the degree to which the null hypothesis is false. The larger the effect size, the greater the certainty with which the null hypothesis can be rejected and the more clinically relevant the outcome. Small, medium, and large effect sizes were defined as 0.2, 0.5 and 0.8, respectively. This definition was applied for a small effect size (d = 0.2) in a small but representative sample of feasibility study data collected in up to 13 participants distributed across 3 sites to define a responder for the various primary efficacy outcome measures listed in the table below. The MID was therefore calculated according to the following formula for each outcome measure:

$$\text{MID} = d * \sqrt{(\sigma1^2 + \sigma2^2) / 2}$$

To be deemed a responder, a participant had to meet or exceed the values listed below for the respective outcomes: Outcomes related to the strength domain included the International Standards for Neurological Classification of Spinal Cord Injury Upper Extremity Motor Score [67] (ISNCSCI-UEMS; MID = 2-point improvement), the GRASSP Strength score [64] (GRASSP-Strength; MID = 4-point improvement), Pinch force (MID = greater than or equal to 2.4N improvement), and Grasp force (MID = greater than or equal to 6N improvement). Outcomes related to the functional domain included the GRASSP Prehension Performance score [64] (MID = 2-point improvement) and the Capabilities of Upper Extremity Test [68] (CUE-T; MID = 4-point improvement).

3. Extended Data Tables

Extended Data Table 1 | Additional Domains within Primary and Secondary End Points (Modified Intention-to-Treat Population)

	Rehabilitation Alone Δ Baseline		ARC^EX Therapy Δ Baseline		ARC^EX Difference Δ month 4- month 2	
	Mean ± SD	*p-value*	*Mean ± SD*	*p-value*	*Mean ± SD*	*p-value*
Pinch Force*	2.6 ± 16.7	0.351	7.3 ± 19.6	0.008	4.8 ± 16.1	0.002
Grasp Force*	10.5 ± 28.3	0.003	24.2 ± 35.0	< 0.001	13.7 ± 27.4	< 0.001
GRASSP Strength*	3.6 ± 6.6	< 0.001	6.4 ± 7.7	< 0.001	2.8 ± 5.4	< 0.001
Upper Extremity Motor Score* (UEMS)	0.2 ± 2.9	0.292	2.3 ± 3.8	< 0.001	2.2 ± 3.2	< 0.001
GRASSP Prehension Performance*	1.3 ± 3.7	0.004	2.9 ± 3.8	< 0.001	1.6 ± 2.9	< 0.001
CUE-T*	5.8 ± 6.5	< 0.001	11.1 ± 7.1	< 0.001	5.3 ± 5.3	< 0.001
Total Sensory Score (TSS)†	-1.8 ± 19.9	0.757	7.8 ± 21.0	0.003	9.6 ± 15.1	< 0.001
Upper Extremity Sensory Score	-0.2 ± 5.9	0.620	2.6 ± 6.8	0.002	2.9 ± 4.8	< 0.001
GRASSP Prehension Ability	1.8 ± 2.8	<0.001	2.6 ± 2.6	< 0.001	0.8 ± 2.3	0.003
GRASSP Sensibility	0.0 ± 1.8	0.313	0.9 ± 2.3	0.002	0.9 ± 2.4	0.003

Plus–minus values are means ± SD. The modified intention-to-treat population included all participants who underwent at least 24 sessions (minimum 12 sessions per month) during the rehabilitation alone period and at least 24 sessions during the ARC^{EX} Therapy period (minimum 12 sessions per month). One-sided paired t-tests or Wilcoxon signed ranks tests were performed for each comparison, as appropriate (Rehabilitation alone: baseline to month 2; ARC^{EX} Therapy: baseline to month 4; and ARC^{EX} difference: month 2 to month 4). * included in primary efficacy outcome. † included in secondary efficacy outcomes.

Extended Data Table 2 | Patient-Reported Surveys Related to Quality of Life, Activities of Daily Living and Impressions of Change (Modified Intention-to-Treat Population)

	Rehabilitation Alone Δ Baseline		ARC^EX Therapy Δ Baseline		ARC^EX Difference Δ month 4- month 2	
	Mean ± SD	*p-value*	*Mean ± SD*	*p-value*	*Mean ± SD*	*p-value*
SCIM III Self-Care Subscore (0-20)	0.3 ± 2.2	0.253	0.6 ± 2.4	0.056	0.3 ± 1.1	0.007
SCIM III Upper Extremity Items (0-13)	0.2 ± 1.6	0.292	0.4 ± 1.6	0.029	0.3 ± 0.9	0.03
Patient Global Impression of Change (PGIC)	4.0 ± 1.1		3.4 ± 1.2		-0.6 ± 1.1	< 0.001
Clinician Global Impression of Change (CGIC)	4.2 ± 0.7		3.7 ± 1.1		-0.6 ± 0.9	<0.001
Patient Health Questionnaire-9	-0.5 ± 2.6	0.144	-0.8 ± 3.2	0.019	-0.4 ± 2.9	0.164

Plus–minus values are means ± SD. The modified intention-to-treat population included all participants who underwent at least 24 sessions (minimum 12 sessions per month) during the rehabilitation alone period and at least 24 sessions during the ARC^{EX} Therapy period (minimum 12 sessions per month). One-sided paired t-tests or Wilcoxon signed ranks tests were performed for each comparison, as appropriate (Rehabilitation alone: baseline to month 2; ARC^{EX} Therapy: baseline to month 4; and ARC^{EX} difference: month 2 to month 4).

Extended Data Table 3 | Spinal Cord Injury Sequelae (Modified Intention-to-Treat Population)

	Rehabilitation Alone Δ Baseline		ARC^{EX} Therapy Δ Baseline		ARC^{EX} Difference Δ month 4- month 2	
	Mean ± SD	*p-value*	*Mean ± SD*	*p-value*	*Mean ± SD*	*p-value*
**Spasticity**						
Penn Spasm Frequency Scale (Frequency)	-0.1 ± 0.8	0.21	-0.3 ± 0.9	0.009	-0.2 ± 0.7	0.011
Penn Spasm Frequency Scale (Severity)	-0.1 ± 0.5	0.15	-0.2 ± 0.7	0.04	-0.1 ± 0.6	0.11
**Pain**						
NRS Pain within last 24 hours	-0.1 ± 1.5	0.353	-0.3 ± 1.6	0.073	-0.2 ± 1.6	0.04
ISCIPDS – Sleep Interference within last week	-0.4 ± 2.6	0.104	-0.9 ± 2.6	0.002	-0.6 ± 2.3	0.06
**Sleep – MOS Scale**						
Shortness of Breath/Headache	-1.0 ± 12.5	0.311	-5.3 ± 18.7	0.017	-4.3 ± 16.9	0.03
Snoring	-6.8 ± 20.6	0.002	-10.5 ± 24.7	<0.001	-3.7 ± 20.1	0.09
Sleep Disturbance	-1.3 ± 15.5	0.264	-3.3 ± 16.4	0.034	-2.1 ± 13.4	0.12
Sleep Problems Index I	-0.3 ± 9.9	0.398	-2.7 ± 12.4	0.051	-2.3 ± 10.1	0.04
Sleep Problems Index II	-0.9 ± 9.9	0.135	-2.8 ± 12.7	0.047	-1.9 ± 9.6	0.07

Plus–minus values are means ± SD. The modified intention-to-treat population included all participants who underwent at least 24 sessions (minimum 12 sessions per month) during the rehabilitation alone period and at least 24 sessions during the ARC_{EX} Therapy period (minimum 12 sessions per month). One-sided paired t-tests or Wilcoxon signed ranks tests were performed for each comparison, as appropriate (Rehabilitation alone: baseline to month 2; ARC_{EX} Therapy: baseline to month 4; and ARC_{EX} difference: month 2 to month 4). Decreases in these outcomes represent improvements.

Extended Data Table 4 | Detailed Listing of Adverse Events (Safety Population).*

Events (MedDRA 23.0 SOC Preferred Term)	Prior to rehabilitation	Rehabilitation alone	ARC^{EX} Therapy	Anytime
**Eye disorders**	0	1	0	1
Eye pain	0	1	0	1
**Gastrointestinal disorders**	0	11	8	19
Haemorrhoids	0	0	1	1
Inguinal hernia	0	1	0	1
Vomiting	0	1	0	1
Dyspepsia	0	2	0	2
Abdominal pain upper	0	1	2	3
Nausea	0	2	1	3
Toothache	0	1	2	3
Constipation	0	3	2	5
**General disorders administration site conditions**	0	3	10	13
Asthenia	0	0	1	1
Inflammation	0	0	1	1
Malaise	0	0	1	1

Medical device site reaction	0	0	1	1
Peripheral swelling	0	0	1	1
Pyrexia	0	0	1	1
Pain	0	0	2	2
Fatigue	0	3	2	5
Hepatobiliary disorders	0	1	0	1
Cholelithiasis	0	1	0	1
Immune system disorders	0	0	1	1
Drug hypersensitivity	0	0	1	1
Infections and Infestations	3	22	18	43
Infection	0	0	1	1
Lower respiratory tract infection	0	1	0	1
Onchomycosis	0	1	0	1
Respiratory tract infection	0	1	0	1
Tooth infection	0	0	1	1
COVID-19	0	2	6	8
Cystitis	0	3	1	4
Urinary tract infection	3	14	9	26

Ear and labyrinth disorders	0	0	1	1
Ear infection, ear pain	0	0	1	1
Injury, poisoning and procedural complications	2	18	13	33
Exposure to SARS-Co-V-2	0	0	1	1
Scratch	0	1	0	1
Spinal column injury	0	0	1	1
Thermal burn	0	1	0	1
Tooth injury	0	1	0	1
Wound	0	0	1	1
Limb injury	0	2	0	2
Buttock injury	0	1	2	3
Contusion	0	2	1	3
Autonomic dysreflexia	0	0	4	4
Skin abrasion	0	3	1	4
Skin laceration	1	3	0	4
Fall	1	4	2	7
Investigations	0	2	2	4
Blood pressure decreased	0	1	0	1

Blood pressure increased	0	0	1	1
Heart rate increased	0	1	0	1
Respiratory rate increased	0	0	1	1
**Metabolism and nutrient disorders**	0	0	3	3
Decreased appetite	0	0	1	1
Dehydration	0	0	1	1
Gout	0	0	1	1
**Musculoskeletal and connective tissue disorders**	0	21	23	44
Arthralgia	0	1	0	1
Joint stiffness	0	0	1	1
Musculoskeletal chest pain	0	1	0	1
Musculoskeletal stiffness	0	1	2	3
Myalgia; musculoskeletal stiffness	0	0	1	1
Back pain	0	3	1	4
Neck pain	0	1	4	5
Myalgia	0	2	4	6
Pain in extremity	0	3	3	6
Muscle spasms	0	3	4	7

Muscle spasms; pain	0	2	0	2
Musculoskeletal pain	0	4	3	7
Nervous system disorders	0	5	13	18
Burning sensation	0	0	1	1
Poor quality sleep	0	0	1	1
Sensory loss	0	1	0	1
Sensory overload	0	0	1	1
Neuralgia	0	1	1	2
Paraesthesia	0	0	2	2
Headache	0	1	2	3
Dizziness	0	2	5	7
Psychiatric disorders	0	2	1	3
Attention deficit hyperactivity disorder	0	0	1	1
Depressed mood	0	1	0	1
Emotional distress	0	1	0	1
Renal and urinary disorders	0	3	12	15
Bladder pain	0	0	1	1
Incontinence	0	2	0	2

Nephrolithiasis	0	0	2	2
Urinary incontinence	0	0	2	2
Urinary tract disorder	0	0	2	2
Bladder spasm	0	1	5	6
Respiratory, thoracic and mediastinal disorders	0	1	1	2
Cough	0	1	1	2
Skin and subcutaneous tissue disorders	0	8	10	18
Eczema	0	1	0	1
Nail cuticle fissure	0	1	0	1
Scab	0	0	1	1
Skin hyperpigmentation	0	1	0	1
Skin irritation	0	0	1	1
Decubitus ulcer	0	1	1	2
Blister	0	3	0	3
Erythema	0	1	3	4
Hyperhidrosis	0	0	4	4
Surgical and medical procedures	0	1	2	3
Wedge resection toenail	0	1	0	1

**Cataract operation**	**0**	**0**	**2**	**2**
**Vascular disorders**	**0**	**6**	**10**	**16**
**Deep vein thrombosis**	**0**	**0**	**1**	**1**
**Pallor**	**0**	**0**	**1**	**1**
**Hypotension**	**0**	**2**	**1**	**3**
**Orthostatic hypotension**	**0**	**2**	**1**	**3**
**Hypertension**	**0**	**2**	**6**	**8**

EXTENDED DATA FIGURE LEGENDS

Extended Data Fig. 1 | Location and stimulation parameters across study participants and sessions. a, Location of the two cathodes with respect to spinal segments. **b,** Profile of electrical stimulation waveforms. **c,** Percent of sessions with monophasic versus biphasic stimulation models. **d,** Range of amplitudes delivered during ARC^{EX} Therapy.

Extended Data Fig. 2 | Responder status for each outcome of the strength and functional domains. Participants were considered responders for each outcome (top panel) if they met the minimally important difference (MID), which was calculated as the change in score between the beginning of the ARC^{EX} Therapy period and the end of the ARC^{EX} Therapy period. The beginning of the ARC^{EX} Therapy period coincides with the end of the rehabilitation alone period. Outcomes related to the functional domain included the Capabilities of Upper Extremity Test [68] (CUE-T; MID = 4-point improvement) and the GRASSP Prehension Performance score [64] (MID = 2-point improvement). Outcomes related to the strength domain included the Pinch force (MID = greater than or equal to 2.4N improvement), Grasp force (MID = greater than or equal to 6N improvement), the GRASSP Strength score [64] (GRASSP-Strength; MID = 4-point improvement), and the International Standards for Neurological Classification of Spinal Cord Injury Upper Extremity Motor Score [67] (ISNCSCI-UEMS; MID = 2-point improvement). To be classified as a "Function Responder" or "Strength Responder" participants must have met the MID criteria for at least one outcome in each domain. To be considered an "Overall Responder", participants must have been classified as both a "Function Responder" and a "Strength Responder". Color indicates responder status for each row.

Extended Data Fig. 3 | Influence of injury severity. The percentage of participants classified as responders versus non-responders are classified based on their American Spinal Injury Association Impairment Scale (AIS) at enrollment.

Extended Data Fig. 4 | Influence of sex. The percentage of participants classified as responders versus non-responders are classified by sex.

Extended Data Fig. 5 | Improvements of hand and arm functions plateau in response to intense rehabilitation well before the end of the rehabilitation alone period. a, During each training session, the participant completed the box and block test. These systematic quantifications allowed to monitor improvements in this task over the rehabilitation alone period. During the first three weeks of the rehabilitation alone period, we detected a significant increase in the scores in the box and block test, normalized to the baseline score at enrollment for each participant. No statistically significant improvement of scores was detected during the following 5 weeks of the rehabilitation alone period. Statistics refers to a repeated measures one-way ANOVA with post hoc testing using the Tukey HSD method. * indicates $p < 0.05$. ** indicates $p < 0.01$. *** indicates $p < 0.001$. n.s. indicates non-significant. Bar graph indicates mean and standard error of the mean for each time point. Statistics provided in **Supplementary Data 3**. **b,** A rolling linear regression coefficient was calculated from the score of each box and block test for each participant using a mixed model linear regression. The dotted line coincides with a coefficient of one, above which improvements remain linear. Dot represents the coefficient of the linear model at each timepoint, and the whiskers represent the standard error of the mean on this model. The linear relationship between training sessions and improvements of scores in the box and block test vanished after three weeks (12 sessions) of rehabilitation alone, wherein the coefficient approached 0. Together these findings reveal the occurrence of a plateau in the improvement of arm and hand functions after three weeks of rehabilitation alone. Since participants performed the box and block test during each session, the initial improvement observed may be partially attributed to increased familiarity with the test through repeated practice.

Extended Data Fig. 6 | Effect of ARC^{EX} Therapy on additional secondary outcomes. Improvements in secondary outcome domains during the rehabilitation alone period, and during the ARC^{EX} Therapy period. Lower values of PGIC, CGIC and PHQ-9 represent improved quality of life. These results suggest that a longer period of ARC^{EX} Therapy may promote additional benefits. Red color indicates the period of ARC^{EX} Therapy. Statistics represent one-way repeated measures ANOVA with Tukey HSD post-hoc testing. * = $p < 0.05$. ** = $p < 0.01$. *** = $p < 0.001$. Line graphs represent the mean and standard error of the mean for each outcome measure. Statistics provided in **Supplementary Data 3**.

Extended Data Fig. 7 | Identification of initial baseline characteristics that best predicted responder status. This analysis included sequential logistic regression models whereby participants were binarized into two groups: above or below a single numerical threshold [78,79]. Odds ratios were then calculated, which reflected the odds of being a responder based on sequential thresholds for each outcome measure included in the primary and secondary effectiveness end points. The sequential models were halted when the odds ratio crossed 1 (black traces), indicating a threshold above which participants demonstrated positive odds of responding to ARC^{EX} Therapy. This analysis revealed cutoffs for ISNCSCI-UEMS (cutoff = 25), Grasp force (cutoff = 100N), Pinch force (cutoff = 25N), CUE-T (cutoff = 40), ISNCSCI Sensory Score (cutoff = 120), ISNCSCI Upper Extremity Sensory Score (cutoff = 40), and GRASSP-Sensibility score (cutoff = 15).
