# Supplementary material for: Non-invasive spinal cord electrical stimulation for arm and hand function in chronic tetraplegia: a safety and efficacy trial
Source: Nat Med. 2024 May 20;30(5):1276–83. doi: 10.1038/s41591-024-02940-9 (PMC11108781; doi:10.1038/s41591-024-02940-9)
Supplement: Supplementary file 2 — Reporting Summary [file 41591_2024_2940_MOESM2_ESM.pdf]

nature portfolio

Corresponding author(s): Gregoire Courtine

Last updated by author(s): Mar 25, 2024

Reporting Summary

Nature Portfolio wishes to improve the reproducibility of the work that we publish. This form provides structure for consistency and transparency in reporting. For further information on Nature Portfolio policies, see our Editorial Policies and the Editorial Policy Checklist.

Statistics

For all statistical analyses, confirm that the following items are present in the figure legend, table legend, main text, or Methods section.

n/a	Confirmed	
☐	☒	The exact sample size (*n*) for each experimental group/condition, given as a discrete number and unit of measurement
☐	☒	A statement on whether measurements were taken from distinct samples or whether the same sample was measured repeatedly
☐	☒	The statistical test(s) used AND whether they are one- or two-sided<br>*Only common tests should be described solely by name; describe more complex techniques in the Methods section.*
☐	☒	A description of all covariates tested
☐	☒	A description of any assumptions or corrections, such as tests of normality and adjustment for multiple comparisons
☐	☒	A full description of the statistical parameters including central tendency (e.g. means) or other basic estimates (e.g. regression coefficient) AND variation (e.g. standard deviation) or associated estimates of uncertainty (e.g. confidence intervals)
☐	☒	For null hypothesis testing, the test statistic (e.g. *F*, *t*, *r*) with confidence intervals, effect sizes, degrees of freedom and *P* value noted<br>*Give P values as exact values whenever suitable.*
☒	☐	For Bayesian analysis, information on the choice of priors and Markov chain Monte Carlo settings
☒	☐	For hierarchical and complex designs, identification of the appropriate level for tests and full reporting of outcomes
☐	☒	Estimates of effect sizes (e.g. Cohen's *d*, Pearson's *r*), indicating how they were calculated

Our web collection on statistics for biologists contains articles on many of the points above.

Software and code

Policy information about availability of computer code

Data collection	The trial was conducted at 14 sites located in the United States, Canada, United Kingdom, and the Netherlands. The trial and recruitment materials were approved by institutional review boards or ethics committees at each trial site, as well as central approval from Advarra institutional review board. An independent Data Safety Monitoring Board (DSMB) regularly reviewed the ongoing trial and could advise the sponsor to stop the trial for safety. The trial was sponsored by Onward Medical, which managed the trial through Contract Research Organizations, provided the ARCEX devices, and provided field support for study investigators. Statistical analysis was performed by an independent statistician. All participants provided written informed consent.
Data analysis	Analyses were performed with the use of SAS software, version 9.4 (SAS Institute). Details are provided in the Supplementary Appendix.

For manuscripts utilizing custom algorithms or software that are central to the research but not yet described in published literature, software must be made available to editors and reviewers. We strongly encourage code deposition in a community repository (e.g. GitHub). See the Nature Portfolio guidelines for submitting code & software for further information.

Data

Policy information about availability of data

All manuscripts must include a data availability statement. This statement should provide the following information, where applicable:

- Accession codes, unique identifiers, or web links for publicly available datasets
- A description of any restrictions on data availability
- For clinical datasets or third party data, please ensure that the statement adheres to our policy

Data that supports the findings will be made available upon reasonable request to the corresponding authors and in Supplementary Data 1.

Research involving human participants, their data, or biological material

Policy information about studies with human participants or human data. See also policy information about sex, gender (identity/presentation), and sexual orientation and race, ethnicity and racism.

Reporting on sex and gender	All demographic information is provided in demographic tables.
Reporting on race, ethnicity, or other socially relevant groupings	Not relevant.
Population characteristics	No covariates were included in the pre-registered statistical analysis.
Recruitment	The trial was conducted at 14 sites located in the United States, Canada, United Kingdom, and the Netherlands. The trial and recruitment materials were approved by institutional review boards or ethics committees at each trial site, as well as central approval from Advarra institutional review board. An independent Data Safety Monitoring Board (DSMB) regularly reviewed the ongoing trial and could advise the sponsor to stop the trial for safety. The trial was sponsored by Onward Medical, which managed the trial through Contract Research Organizations, provided the ARCEX devices, and provided field support for study investigators. Statistical analysis was performed by an independent statistician. All participants provided written informed consent. Each site was responsible for their own recruitment activities. Templates for recruitment (online/paper) were provided to the sites by Onward. All recruitment activities were approved by the local IRBs/ethics committees.
Ethics oversight	The trial and recruitment materials were approved by institutional review boards or ethics committees at each trial site, as well as central approval from Advarra institutional review board.

Note that full information on the approval of the study protocol must also be provided in the manuscript.

Field-specific reporting

Please select the one below that is the best fit for your research. If you are not sure, read the appropriate sections before making your selection.

☒ Life sciences ☐ Behavioural & social sciences ☐ Ecological, evolutionary & environmental sciences

For a reference copy of the document with all sections, see nature.com/documents/nr-reporting-summary-flat.pdf

Life sciences study design

All studies must disclose on these points even when the disclosure is negative.

Sample size	A statistical plan was discussed and agreed upon with the FDA. The pre-registered statistical plan is provided in Supplementary Information. A sample of 65 participants was calculated assuming a minimum power of 80%, a two-sided type I error of 10%, a responder rate of 67%, a performance goal of 50%, and a 25% drop out rate. All effectiveness end points were assessed within prespecified modified-intent-to-treat populations, wherein only participants that underwent at least 24 sessions (average of 12 sessions per month) during the rehabilitation alone period and at least 24 sessions during the ARCEX Therapy period were included in the analysis. This minimum number of exposures to both interventions was required to perform comparisons between the outcomes of the rehabilitation alone and ARCEX Therapy periods. All participants exposed to a study procedure were included in the safety analysis population.
Data exclusions	From January 14 to December 24, 2021, a total of 65 participants underwent screening, and were enrolled in the Up-LIFT trial (Fig. 2). Sixty participants had completed the entire protocol and assessments by the end of June, 2022. One participant withdrew from the study prior to any study procedures, two withdrew during the rehabilitation alone period for personal reasons unrelated to the study, and two withdrew during the ARCEX Therapy period, one due to protocol non-adherence and one for personal reasons.
Replication	The nature of this safety and efficacy trial did not require replication.
Randomization	No randomization was performed.
Blinding	All statistical analysis was performed by an independent blinded statistician.

Reporting for specific materials, systems and methods

We require information from authors about some types of materials, experimental systems and methods used in many studies. Here, indicate whether each material, system or method listed is relevant to your study. If you are not sure if a list item applies to your research, read the appropriate section before selecting a response.

Materials & experimental systems

n/a	Involved in the study
☒	☐ Antibodies
☒	☐ Eukaryotic cell lines
☒	☐ Palaeontology and archaeology
☒	☐ Animals and other organisms
☐	☒ Clinical data
☒	☐ Dual use research of concern
☒	☐ Plants

Methods

n/a	Involved in the study
☒	☐ ChIP-seq
☒	☐ Flow cytometry
☒	☐ MRI-based neuroimaging

Clinical data

Policy information about clinical studies

All manuscripts should comply with the ICMJE guidelines for publication of clinical research and a completed CONSORT checklist must be included with all submissions.

Clinical trial registration

NCT04697472

Study protocol

Supplementary Appendix.

Data collection

From January 14 to December 24, 2021, a total of 65 participants underwent screening, and were enrolled in the Up-LIFT trial (Fig. 2). Sixty participants had completed the entire protocol and assessments by the end of June, 2022. One participant withdrew from the study prior to any study procedures, two withdrew during the rehabilitation alone period for personal reasons unrelated to the study, and two withdrew during the ARCEX Therapy period, one due to protocol non-adherence and one for personal reasons. All clinical sites are listed in the study protocol.

Outcomes

The primary effectiveness end point tested the hypothesis that the majority of the participants would demonstrate significant improvements in both strength and functional performance domains from the end of the rehabilitation alone period to the end of the ARCEX Therapy period. Participants were considered responders if they met minimally important difference (MID) criteria determined with Cohen's effect size method 66 for at least one outcome in each of the strength and functional performance domains. Outcomes related to the strength domain included the International Standards for Neurological Classification of Spinal Cord Injury Upper Extremity Motor Score 67 (ISNCSCI-UEMS; MID = 2-point improvement), the GRASSP Strength score 64 (GRASSP-Strength; MID = 4-point improvement), Pinch force (MID = greater than or equal to 2.4N improvement), and Grasp force (MID = greater than or equal to 6N improvement). Outcomes related to the functional domain included the GRASSP Prehension Performance score 64 (MID = 2-point improvement) and the Capabilities of Upper Extremity Test 68 (CUE-T; MID = 4-point improvement). The primary safety end point for the Up-LIFT trial was the incidence of serious adverse events (SAEs) related to the use of ARCEX Therapy.

Secondary effectiveness end points included the superiority of responder rates following completion of ARCEX Therapy compared to during the rehabilitation alone period, as well as changes in single outcomes between enrollment and the end of the rehabilitation alone period compared to between enrollment and the end of the ARCEX Therapy period. These secondary effectiveness end points were hierarchically ordered a priori in the following sequence: Pinch force, GRASSP-Prehension Performance score 64, GRASSP-Strength score 64, ISNCSCI-UEMS 67, ISNCSCI Total sensory score 67, the EuroQol five-dimensional five-level (EQ-5D-5L) score 69, Spinal Cord Independence Measure (SCIM III) 70, and the Abbreviated World Health Organization Quality of Life questionnaire (WHOQOL-Bref) score 71. The secondary safety end point was the incidence of all adverse events (AEs) and SAEs in the trial. Exploratory end points included additional outcomes that measured changes in the quality of life and the long-term consequences of SCI. These outcomes included the Numerical Rating Scale (NRS) for pain, the International Spinal Cord Injury Pain Data Set (ISCIPDS) 72, the Medical Outcomes Study (MOS) Sleep Scale 73, a subset of scores within the Spinal Cord Independence Measure (SCIM III) 70, the GRASSP Sensibility Score 64, the Penn Spasm Frequency Scale (PSFS) 74, subset scores within the EuroQol 5 Dimension 5 Level Questionnaire (EQ-5D-5L) 69 and the WHOQOL-BREF 71, the International Standards to document remaining Autonomic Function after Spinal Cord Injury (ISAFSCI) 75, the Patient Health Questionnaire (PHQ-9) 76, and the Global Impression of Change (Clinician and Patient) 77. The functional profile of responders versus non-responders was also explored.

Plants

Seed stocks	*Report on the source of all seed stocks or other plant material used. If applicable, state the seed stock centre and catalogue number. If plant specimens were collected from the field, describe the collection location, date and sampling procedures.*
Novel plant genotypes	*Describe the methods by which all novel plant genotypes were produced. This includes those generated by transgenic approaches, gene editing, chemical/radiation-based mutagenesis and hybridization. For transgenic lines, describe the transformation method, the number of independent lines analyzed and the generation upon which experiments were performed. For gene-edited lines, describe the editor used, the endogenous sequence targeted for editing, the targeting guide RNA sequence (if applicable) and how the editor was applied.*
Authentication	*Describe any authentication procedures for each seed stock used or novel genotype generated. Describe any experiments used to assess the effect of a mutation and, where applicable, how potential secondary effects (e.g. second site T-DNA insertions, mosiacism, off-target gene editing) were examined.*
