# Supplementary material for: Non-invasive spinal cord electrical stimulation for arm and hand function in chronic tetraplegia: a safety and efficacy trial
Source: Nat Med. 2024 May 20;30(5):1276–83. doi: 10.1038/s41591-024-02940-9 (PMC11108781; doi:10.1038/s41591-024-02940-9)
Supplement: Supplementary file 5 — Pre-registered clinical protocol. [file 41591_2024_2940_MOESM5_ESM.pdf]

Up-LIFT Study

Clinical Investigational Plan (CIP)

**Study Title**	**Clinical assessment of upper extremity performance in individuals with spinal cord injury using the LIFT System to deliver non-invasive electrical spinal stimulation (ARC Therapy)**

LIST OF ABBREVIATIONS

AD	Autonomic Dysreflexia
ADL	Activities of Daily Living
ADE	Adverse Device Effect
AE	Adverse Event
ASIA	American Spinal Injury Association
CDE	Common Data Elements
CFR	Code of Federal Regulations
CIP	Clinical Investigative Protocol
CUE-Q/T	Capabilities of Upper Extremity – Questionnaire/Test
CRF	Case Report Form
DSMB	Data and Safety Monitoring Board
EMG	Electromyogram
FDA	Food and Drug Administration
GCP	Good Clinical Practice
FTP	Functional Task Practice
GRASSP	Graded Redefined Assessment of Strength, Sensibility and Prehension
ISAFSCI	International Standards to document remaining Autonomic Function after Spinal Cord Injury
ISCoS	International Spinal Cord Society
ISNCSCI	International Standards for Neurological Classification of Spinal Cord Injury
IRB	Institutional Review Board
ITT	Intent to Treat
ISO	International Organization for Standardization
LCD	Liquid Crystal Display
LED	Light Emitting Diode
MCID	Minimal Clinically Important Difference
MDD	Minimal Detectable Difference
MEP	Motor Evoked Potential
MID	Minimal Important Difference
mITT	Modified Intent to Treat
MOSS	Medical Outcomes Sleep Scale
MRI	Magnetic Resonance Imaging
NASCIC	North American Spinal Cord Injury Consortium
NRN	Neurorecovery Network

NRS	Numerical Rating Scale
NSCISC	National Spinal Cord Injury Statistical Center
NSR	Non-Significant Risk
PHQ-9	9-Item Patient Health Questionnaire
PIC	Patient Informed Consent
PSFS	Penn Spasm Frequency Scale
PPP	Per Protocol Population
SAE	Serious Adverse Event
SADE	Serious Adverse Device Effect
SCI	Spinal Cord Injury
SCIM	Spinal Cord Independent Measure
SCS	Spinal Cord Stimulation
SOP	Standard Operating Procedure
UE	Upper Extremity
UEMS	Upper Extremity Motor Score

1. TABLE OF CONTENTS

1. TABLE OF CONTENTS 9
2. CLINICAL INVESTIGATION SYNOPSIS 14
3. INTRODUCTION 21
   3.1. Device Name 21
   3.2. Disease State 21
   3.3. ARC Therapy and the LIFT System 22
   3.4. Background 22
   3.5. Rationale for Using ARC Therapy in Spinal Cord Injury 24
   3.6. Prior Clinical Experience with the LIFT System 25
   3.7. List of AEs and SAEs Reported in the Feasibility Studies Using the LIFT System 31
   3.8. Summary 32
4. INTENDED USE AND DEVICE DESCRIPTION 32
   4.1. Intended Use 32
   4.2. Device Description 32
      4.2.1. Components and Parameters 34
5. STUDY PURPOSE AND OBJECTIVES 36
   5.1. Study Purpose 36
   5.2. Study Objectives 36
      5.2.1. Safety 36
      5.2.2. Effectiveness 36
      5.2.3. Other 37
   5.3. Standard of Care: Functional Task Practice 37
6. STUDY ENDPOINTS 37
   6.1. Rationale for the Selection of Study Endpoints 37
   6.2. Primary Endpoint 39
      6.2.1. Safety 39
      6.2.2. Effectiveness 39
   6.3. Secondary Endpoints 39
   6.4. Observational Endpoints 40
   6.5. Rationale for the Selection of Observational Safety Data Reporting 40

7. Study Design ... 41
7.1. Investigational Sites ... 44
7.2. Study Assessments and Intervals ... 44
7.3. Subject Withdrawal ... 45
8. CRITERIA FOR ELIGIBILITY ... 45
8.1. Inclusion Criteria ... 45
8.2. Exclusion Criteria ... 45
9. SCHEDULE OF EVENTS ... 47
10. STUDY PROCEDURES ... 48
10.1. Screening and Enrollment ... 48
10.2. Baseline Evaluations ... 48
10.3. Functional Task Practice (Wash-in Period) ... 48
10.3.1. Interim FTP Assessment ... 49
10.3.2 Final FTP Assessment ... 49
10.4. Functional Task Practice with ARC Therapy (Stimulation Period) ... 49
10.4.1. Interim FTP+ARC Therapy Assessment ... 51
10.4.2. Final FTP+ARC Therapy Assessment – Primary Endpoint Visit ... 51
10.5. Follow-up Assessment (optional) ... 51
10.6. Medications ... 52
10.7. Medical History ... 52
10.8. COVID-19 Accommodations ... 52
10.9. Early Withdrawal or Early Termination Evaluation ... 52
11. STUDY OUTCOMES ... 53
11.1. International Standard Neurological Classification of Spinal Cord Injury (ISNCSCI) ... 53
11.2. Graded Refined Assessment of Strength, Sensibility and Prehension (GRASSP) ... 53
11.3. Pinch and Grasp Force ... 54
11.4. Capabilities of Upper Extremity Test (CUE-T) ... 54
11.5. Numerical Rating Scale for Pain ... 55
11.6. International Spinal Cord Injury Pain Data Set (ISCIPDS) ... 55
11.7. Medical Outcomes Study (MOS) Sleep Score ... 55
11.8. Spinal Cord Independence Measure (SCIM III) ... 55
11.9. Penn Spasm Frequency Scale (PSFS) ... 56
11.10. 5-Dimension, 5-Level European Quality of Life (EQ-5D-5L) ... 56

11.11. World Health Organization Quality of Life Measure (WHOQOL-BREF) 56
11.12. International Standards to document remaining Autonomic Function after Spinal Cord Injury (ISAFSCI) 57
11.13. 9-Item Patient Health Questionnaire 57
11.14. Patient/Clinician Global Impression of Change (PGIC/CGIC) 57
11.15. Motor Evoked Potential (MEP) 57
12. SUBJECT BENEFITS AND RISKS 58
12.1. Potential Benefits to Study Subjects 58
12.2. Potential Risks to Study Subjects 58
12.3. Data Safety Monitoring Board 58
12.4. Clinical Risk Analysis 58
12.4.1. Increased Risks to Subjects Posed by the Investigation 58
12.4.2. Manner in Which Risks Will be Minimized 59
12.4.3. Justification for the Investigation 61
12.4.4. Description of Patient Population 61
12.4.5. Adverse Events 62
12.5. Conclusion 62
13. SAFETY DEFINITIONS AND REPORTING REQUIREMENTS 62
13.1. Adverse Events 62
13.2. Serious Adverse Events 63
13.3. Hospitalizations 63
13.4. Primary Serious Adverse Events 63
13.5. Anticipated Adverse Events Associated with ARC Therapy 64
13.6. Adverse Device Effects 67
13.7. Serious Adverse Device Effects 67
13.8. Anticipated Serious Adverse Device Effects 67
13.9. Unanticipated Serious Adverse Device Effects 67
13.10. Handling and Reporting of Adverse Events and Adverse Device Effects 67
13.11. Severity 69
13.12. Relationship 69
13.13. Device Malfunction/Failure/Deficiency 70
13.14. Early Termination or Suspension of the Clinical Investigation 71
13.15. Procedures for Termination or Suspension 71

14. STATISTICAL ANALYSIS 72
14.1. Sample Size 72
14.2. Statistical Analyses 72
14.2.1. General Considerations 72
14.2.2. Descriptive Statistics 73
14.2.3. Study Day 73
14.2.4. Visit Windows 73
14.2.5. Statistical Significance 73
14.3. Analysis Population 73
14.4. Subject Disposition 74
14.5. Demographics and Baseline Characteristics 74
14.6. Primary Endpoints 74
14.6.1. Primary Safety Endpoint 74
14.6.2. Primary Effectiveness Endpoint 75
14.7. Secondary Endpoints 75
14.7.1. Secondary Safety Endpoint 75
14.7.2. Secondary Effectiveness Endpoint 75
14.8. Sensitivity Analyses 76
14.8.1. Primary Endpoint 76
14.8.2. Validation of Minimal Important Difference (MID) Criterion 77
14.9. Poolability Analyses 77
14.10. Safety Analyses 77
14.11. Interim Analyses 77
14.12. Protocol Deviations 77
14.13. Other Analyses 78
14.14. Follow-up Analyses 78
14.15. Changes from Planned Analyses 78
14.16. Subject Listings 78
14.17. Missing Data 78
14.17. Early Termination Evaluation 79
14.18. Subgroup Analysis 79
15. DATA QUALITY ASSURANCE 79
15.1. Handling and Storage of Data and Documents 79

15.2. Case Report Forms 80
15.3. Deviations 80
15.4. Monitoring Procedures and Visits 81
15.5. Clinical Investigational Plan Amendments 81
15.6. Data Management 82
15.7. Device Accountability 82
15.8. Study Reports 82
16. ETHICS 83
16.1. Declaration of Helsinki 83
16.2. Institutional Review Board or Independent Ethics Committee Approval 83
16.3. Informed Consent 83
16.4. Subject Identification and Confidentiality 83
17. REGULATORY REQUIREMENTS 84
17.1. Compliance with Regulations Applicable to Clinical Trials 84
17.1.1. Standards 84
17.1.2. Regulatory Documents 84
17.1.3. Subject Data Protection 84
17.1.4. Adverse Event Monitoring and Reporting 85
18. CHANGES TO THE CIP OR RELATED PROCEDURES 85
19. DEVIATIONS FROM THE CIP 85
20. GENERAL CONSIDERATIONS 85
20.1. Discontinuation of the Study 85
20.2. Use of Information and Publication 85
21. REFERENCES 87
22. APPENDICES 91
22.1. Appendix A. Data Safety Monitoring Board (DSMB) 91
22.2. Appendix B. Results of Safety Literature Review on ARC or Equivalent Therapies 92
22.3 Appendix C: Publication Policy 95
23. TABLES 97
24. FIGURES 98

2. CLINICAL INVESTIGATION SYNOPSIS

**Short Title**	Up-LIFT Study
**Study Title**	Clinical assessment of upper extremity performance in individuals with spinal cord injury using the LIFT System to deliver non-invasive electrical spinal stimulation (ARC Therapy).
**Sponsor**	ONWARD Medical, Inc.
**Device Description**	The LIFT System includes a Stimulator, Battery Charger, Electrodes, Programmer, Connecting Cables and accessories. A description of the system is provided below:<br>• Stimulator – The Stimulator is a 4-channel device that delivers a mono- or bi-phasic charge balanced waveform at constant current, pulsed stimulation through electrodes placed on the skin. Each channel is independent and intended to be connected to a separate pair of electrodes. The Stimulator delivers current through the channel output and uses a separate electrode as the return. Each channel may be configured independently with a unique current level, frequency, waveform, and various timing parameters. A program may be defined that specifies the length of time therapy is delivered and the preconfigured channels for a therapy session. The Stimulator is powered by an internal rechargeable battery. The Stimulator has an LCD display screen, functional touch sensitive buttons, LED and audible indicators, battery charger connector port, and a removable electrode cable assembly.<br>• Battery Charger – The Battery Charger is a direct wired charger that recharges the Stimulator batteries through the charger connector port.<br>• Electrodes – Commercially available hydrogel-based electrodes are affixed to the subject's skin midline over the spine using spinous processes as landmarks and are connected to the Stimulator's electrode cable assembly. Stimulation is delivered using cathodes as active contacts and two anodes serving as return electrodes. Cathode electrodes are placed midline on the skin of the neck, one above and one below the injury site. Additional cathode electrodes may also be placed at other vertebral positions, e.g., at the thoracic level to aid with trunk stability. Anode electrodes are placed symmetrically parallel to the iliac crests.<br>• Programmer – The Programmer is an off-the-shelf tablet-based computer with proprietary software used by physicians and clinical personnel that wirelessly communicates with and programs the Stimulator. The Programmer allows the Stimulator to be configured with programs that deliver therapy to the subject. The Programmer has the

	capability to update the Stimulator firmware and retrieve device log file data.<br><br>• Connecting Cables – The connecting cables transmit the stimulation energy to single-use hydrogel electrodes affixed to the subject's skin.
**Indication for Use**	The LIFT System is intended to be used to improve or restore upper extremity sensory and motor function in people with chronic neurological deficit resulting from incomplete spinal cord injury. The LIFT System is intended to be used in conjunction with physical or occupational therapy at the hospital and at a rehabilitation therapy clinic.
**Study Design**	The Up-LIFT Study is a prospective, single-arm study designed to evaluate the safety and effectiveness of non-invasive electrical spinal stimulation (ARC Therapy) administered by the LIFT System to treat upper extremity functional deficits in people with chronic tetraplegia. The primary endpoint of this pivotal study will report device related safety and changes in established metrics of upper extremity function and strength after treatment with the study device.<br><br>To ensure that the benefits realized in the study are directly attributable to the ARC Therapy administered by the LIFT System, all enrolled subjects will first undergo a guided, in-clinic conventional functional task practice (FTP) program lasting approximately two months to regain strength and function of their upper extremities (UE). Performance gains realized during the wash-in period provide a subject specific control that reflects the limits of conventional FTP without stimulation (standard of care). At the conclusion of the wash-in period, subjects will complete pre-stimulation baseline testing of UE function.<br><br>To test the additive benefit of training with stimulation, combined FTP and ARC Therapy will then be administered over a period of approximately two months using the LIFT System. FTP will follow established rehabilitation protocols that are specific to the individual subject's specific needs and capabilities (Gomes-Osman, Tibbett, Poe, & Field-Fote, 2017). Training will be graded to accommodate performance improvement over time, thus maximizing the potential benefit to subjects. To ensure consistency and safety, subjects will participate in a minimum of 12 and a maximum of 20 in-clinic training sessions per month. At the conclusion of this primary training period, changes in UE strength and function will be measured without active stimulation therapy and used to assess the primary study endpoints.<br><br>The choice of primary outcome measures for this pivotal study is dictated by the following factors –<br>1. Safety,<br>2. Relevance to UE function,<br>3. Capture improvements in both strength and function, and

<table>
<tr><td></td><td>4. Magnitude of changes that are clinically meaningful

All performance metrics will be assessed with stimulation off at enrollment, at the completion of the wash-in period and at the end of the ARC Therapy assessment period. An optional final set of assessments will be performed during a post-treatment follow-up visit to evaluate the immediate neuroprosthetic effect of active stimulation on strength and motor function. Subjects with clinically meaningful gains in multiple performance domains resulting from the ARC Therapy with LIFT will be considered responders. Additionally, gains during the wash-in (control) period will be compared to gains during the ARC Therapy with LIFT (test) period. Safety will be evaluated throughout the entire study through periodic monitoring and analysis of all reported adverse events.</td></tr>
<tr><td>Objectives</td><td>Safety: To provide confirmatory evidence that use of the LIFT System, inclusive of all components and accessories, is safe.

Effectiveness: To provide confirmatory evidence that use of the LIFT System provides an effective treatment for the restoration or improvement in UE strength and function.

Other: To provide data regarding the potential benefits of the LIFT System to achieve other secondary outcomes such as improvement in pain, spasticity, quality of life, cardiovascular (blood pressure) and autonomic function.</td></tr>
<tr><td>Study Endpoints</td><td>Primary:

Safety: Observational data regarding the incidence of serious adverse events (SAEs) related to the use of the study device and treatment procedures will be reported.

Effectiveness: The primary effectiveness outcome measure will test the hypothesis that a majority of the subjects will experience clinically significant improvement in selected strength and functional performance metrics after treatment with ARC Therapy administered by the LIFT System and FTP. A subject will be considered a treatment responder if she/he reports clinically relevant improvements in at least one outcome each in the Strength and Function domains as follows:

<table>
<tr><th>Strength</th><th></th><th>Function</th></tr>
<tr><td>ISNCSCI-UEMS</td><td></td><td>GRASSP-Prehension</td></tr>
<tr><td>GRASSP-Strength</td><td>and</td><td>CUE-T</td></tr>
<tr><td>Pinch force</td><td></td><td></td></tr>
<tr><td>Grasp force</td><td></td><td></td></tr>
</table>
</td></tr>
</table>

	**Secondary:**<br>Safety: All adverse events (AEs) and SAEs in the study will be reported.<br>Effectiveness: To capture meaningful improvements in established outcomes assessing upper extremity function, the following hierarchical testing will be carried out. These endpoints will be tested in descending order of importance through hierarchical testing as described in the Statistical Analysis Plan (SAP).<br>• Superiority of combined MPT and ARC Therapy with LIFT vs. FTP alone as described by statistically significant difference in responder rates (comparison of change from enrollment baseline to end of FTP with the change from enrollment baseline to end of combined FTP and ARC Therapy with LIFT)<br>• Quantitative comparison of individual performance metrics to establish superiority of FTP and ARC Therapy with LIFT compared to FTP alone:<br>• Pinch force<br>• GRASSP-Prehension<br>• GRASSP-Strength<br>• ISNCSCI-UEMS<br>• ISNCSCI-Total sensory score<br>• EQ-5D-5L<br>• SCIM<br>• WHOQOL-BREF<br>**Observational:**<br>Descriptive statistics on additional domains within the primary endpoint outcomes will be reported. The following additional assessments impacting patient quality of life and long-term consequences of SCI such as outcomes related to pain, spasticity, quality of life, sleep and bladder/bowel/sexual function will be reported as descriptive statistics and clinically relevant changes, where possible.<br>• Numerical Rating Scale (NRS) for pain<br>• International Spinal Cord Injury Pain Data Set ((ISCIPDS)<br>• Medical Outcomes Study (MOS) Sleep Scale<br>• Spinal Cord Independent Measure (SCIM III)<br>• Penn Spasm Frequency Scale (PSFS)<br>• EuroQol 5 Dimension 5 Level Questionnaire (EQ-5D-5L)<br>• World Health Organization Quality of Life (WHOQOL-BREF)<br>• International Standards to document remaining Autonomic Function after Spinal Cord Injury (ISAFSCI)<br>• Patient Health Questionnaire (PHQ-9)<br>• Global Impression of Change (Clinician and Patient)

	An optional follow-up assessment following completion of the study will be reported to allow for evaluation of the immediate neuroprosthetic effect of active stimulation on strength and motor function.
**Sample Size Justification**	The primary effectiveness endpoint is a responder analysis with a responder defined as a subject who demonstrates improvement in at least 1 outcome in each of the strength and function domains. Assuming a minimum power of 80%, a two-sided type I error of 10% (one-sided 5%), a responder rate of 67%, a 50% performance goal and 25% drop out rate, a sample size of 65 subjects is required to be enrolled in the study across a maximum of fifteen sites. Fifty-two of these subjects are intended to complete their four-month visit to ensure the primary endpoint is statistically powered. Any single site may enroll up to a maximum of 25% of the total sample size.
**Inclusion Criteria**	Subjects must meet all the following criteria:<br>1. At least 22 years old and no older than 75 years old at the time of enrollment<br>2. Non-progressive cervical spinal cord injury from C2-C8 inclusive<br>3. American Spinal Injury Association (ASIA) Impairment Scale (AIS) classification B, C, or D<br>4. Indicated for upper extremity training procedures by subject's treating physician, occupational therapist or physical therapist<br>5. GRASSP-Prehension score ≥10 or GRASSP-Strength score ≥30<br>6. Minimum 12 months post-injury<br>7. If prescribed anti-spasticity or pain medications, must be at stable dose for at least 4 weeks prior to commencing study procedures<br>8. Capable of providing informed consent
**Exclusion Criteria**	Subjects must not meet any of the following criteria:<br>1. Has uncontrolled cardiopulmonary disease or cardiac symptoms as determined by the Investigator<br>2. Has any unstable or significant medical condition that is likely to interfere with study procedures or likely to confound study endpoint evaluations like severe neuropathic pain, depression, mood disorders or other cognitive disorders<br>3. Has been diagnosed with autonomic dysreflexia that is severe, unstable, and uncontrolled<br>4. Requires ventilator support<br>5. Has an autoimmune etiology of spinal cord dysfunction/injury<br>6. History of additional neurologic disease such as stroke, multiple sclerosis, traumatic brain injury, etc.

	7. Peripheral neuropathy (diabetic polyneuropathy, entrapment neuropathy, etc.)<br>8. Spasms that limit the ability of the subjects to participate in the study training as determined by the Investigator<br>9. Received Botulinum toxin injections in their upper extremity, neck, or hand within 6 months prior to enrollment<br>10. Has urinary tract infection or any of the following issues in the upper extremity or the cervical spine region at the time of enrollment: painful musculoskeletal dysfunction unrelated to SCI, unhealed fracture, contracture, pressure sore<br>11. Breakdown in skin area that will come into contact with electrodes<br>12. Presence of syringomyelia as confirmed by an MRI<br>13. Currently undergoing treatment for cancer or has been in remission for less than 2 years<br>14. Received stem cell treatment within the past two years prior to enrollment<br>15. Prior nerve or tendon transfer procedure in the upper extremities<br>16. Total baclofen dose >30 mg per day<br>17. Has any active implanted medical device<br>18. Pregnant, planning to become pregnant or currently breastfeeding<br>19. Concurrent participation in another drug or device trial that may interfere with this study<br>20. Has undergone a prior course of spinal stimulation therapy directed at UE improvement<br>21. In the opinion of the investigators, the study is not safe or appropriate for the participant
**Treatment Schedule**	**Screening and informed consent**<br>Patients' eligibility criteria based on inclusion/exclusion criteria will be reviewed and the investigator will begin the process of patient informed consent for patients who meet all criteria for enrollment.<br>**Enrollment and baseline assessments**<br>Patients who meet all the enrollment criteria and sign the informed consent will be enrolled in the study. Baseline evaluations will be performed once the subject and investigator have established an acceptable schedule for the subject's continuous participation in the study.<br>**Functional task practice training period (wash-in period)**<br>Subjects will visit the clinic for a series of FTP sessions over a period of approximately 2 months. The extent and duration of each session will be guided by the specific needs of the subject but will be graded to maximize the potential benefit of the training. A minimum of 12 and a maximum of 20 sessions per month will be performed.

	The subject will repeat the baseline testing after the first month of FTP and again at the end of the second month or at the completion of required number of training sessions. Adverse events will be collected at each clinic visit.<br>**ARC Therapy training period**<br>Subjects will visit the clinic for a series of FTP and ARC Therapy administered by the LIFT System sessions over a period of approximately 2 months. As before, the extent and duration of each session will be guided by the specific needs of the subjects for a minimum of 12 and a maximum of 20 sessions per month.<br>The subject will repeat the baseline testing after the first month of FTP and ARC Therapy with LIFT and again at the end of the second month or at the completion of a required number of ARC Therapy sessions. Adverse events will be reviewed and collected at each clinic visit.<br>**Post treatment period**<br>To evaluate the immediate neuroprosthetic effect of active stimulation on strength and motor function, a relevant set of assessments will be performed during a post-treatment follow-up with both stimulation off and on. This optional assessment will be scheduled from 1-30 days after the completion of the 4 month evaluation.<br>The subject will complete the study exit form and exit the study. In case of any ongoing AEs, the subject will be followed until the event is resolved or the subject's condition is deemed to have stabilized.
**Criteria for Withdrawal**	All subjects are free to withdraw from participation in this study at any time, for any reason, and without prejudice. Subjects may be withdrawn from the study when they request early discontinuation or are lost to follow-up.<br>The clinical investigator may terminate a subject from the study at any time for lack of therapeutic effect that is intolerable to the subject, or otherwise considered unacceptable, for intolerable or unacceptable AEs, intercurrent illness, noncompliance with study procedures, administrative reasons, or in the clinical investigator's opinion, to protect the subject's best interest.
**Enrollment Period**	Enrollment is expected to last approximately 12 months.
**Study Duration**	The total study duration is approximately 17 months assuming 12 months for subject enrollment and a 5 month follow-up period.

3. INTRODUCTION

3.1. Device Name

The device under clinical investigation is the LIFT System delivering non-invasive electrical spinal stimulation called ARC Therapy.

3.2. Disease State

Traumatic SCI to the cervical spine (vertebral segments C2-C8) results in tetraplegia. Unlike those with paraplegia, the major challenge for individuals with tetraplegia is the loss of function of the trunk and both lower and upper extremities. Injury to higher levels of the cervical spine will cause greater impairment in the upper limbs, and the degree of impairment is reduced as the level of injury moves rostrocaudally from the occiput toward the T1 vertebra (Figure 1).

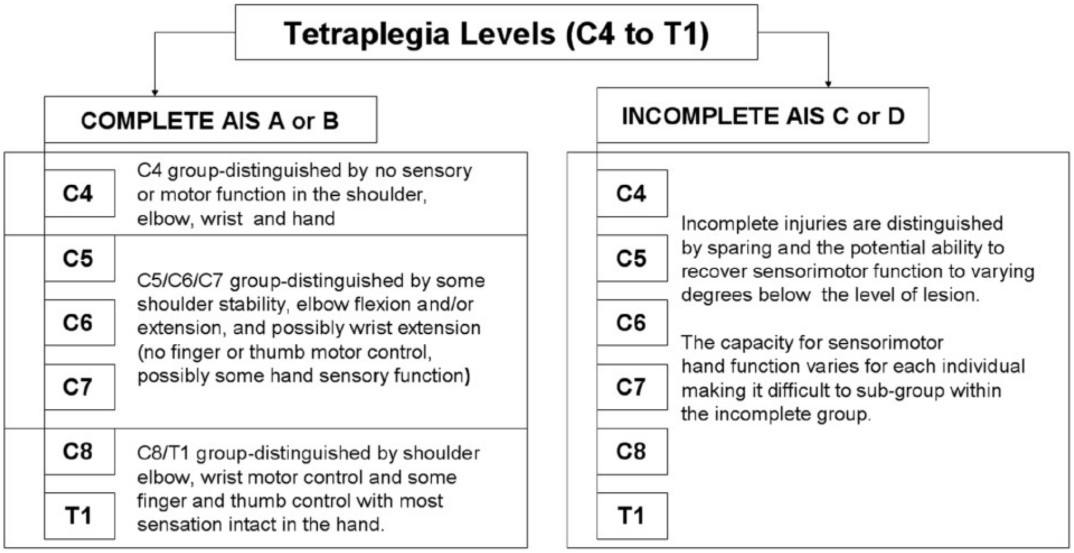

Figure 1. Adapted from Kalsi-Ryan and Verrier, Physiotherapy Canada, 2011

In the case of individuals with complete SCI, motor, or sensory function below the neurological level of injury is often not regained. On the other hand, those with incomplete injuries may spontaneously recover sensory and motor function. However, the degree of recovery and its influence on function are highly unpredictable. In general, a couple of rehabilitation strategies are followed-

- Compensating for functional loss by using remnant capacity of the sensorimotor system
- Restoring lost sensorimotor capacity

ARC Therapy and the LIFT System focus primarily on the second rehabilitative strategy and/or augmenting existing sensorimotor function in individuals with tetraplegia through electrical stimulation.

3.3. ARC Therapy and the LIFT System

Advances in understanding the role of electrical stimulation in spinal cord injury have demonstrated that volitional hand control and reduction in spasticity can be achieved with implantable spinal cord stimulation (SCS) by modulating the motor circuits (Dimitrijevic, et al., 1986) (Dimitrijevic, Illis, Nakajima, Sharkey, & Sherwood, 1986) (Lu, et al., 2016). Differential activation of the motor circuits and the dorsal roots is also possible with transcutaneous spinal cord stimulation. ARC Therapy is one such transcutaneous approach to activate the spinal circuits involved in the propriospinal system which may have a positive impact on motor function. Early feasibility studies have shown that outcomes similar to implantable SCS can be achieved non-invasively using the LIFT System (Gad, et al., 2018).

3.4. Background

Of the 17,000 new cases every year, traumatic SCI results in either complete or incomplete tetraplegia in 58% of them (National Spinal Cord Injury Statistical Center (NSCISC). Spinal Cord Injury Facts and Figures at a Glance. Available from: https://www. nscisc.uab.edu/Public/Facts%202016.pdf , 2016). Etiology of the injury in nearly two-thirds of these cases involve vehicular accidents or falls. Tetraplegia results in loss of hand and arm function which may significantly limit independence with activities of daily living (ADLs) and result in a reduced quality of life. Recovery of this function is often the top priority for these individuals (Anderson, 2004) (Snoek, IJzerman, Hermens, Maxwell, & Biering-Sorensen, 2004) (Lo, Tran, Anderson, Craig, & Middleton, 2016). In persons with tetraplegia, hand muscle force generation is highly correlated with success or failure in the ability to perform common functional tasks (Smaby, et al., 2004). Therefore, it does not come as a surprise that restoration of this function is cited as the highest priority for individuals with SCIs, five times greater than other functions like bladder, bowel, sexual or movement (Anderson, 2004).

For individuals with chronic SCI, along with impaired sensorimotor issues, the secondary complications associated with immobility may present additional challenges. These include complications to respiratory, cardiovascular, urinary, and bowel systems. Additionally, individuals with SCI may experience spasticity, pain, pressure ulcers, osteoporosis and bone fractures leading to significant health complications and reduced quality of life (Sezer, Akkuş, & Uğurlu, 2015).

Respiratory complications include insufficiency of respiratory muscles, ineffective cough, reduced vital capacity, reduced compliance of lung and chest wall resulting in pneumonia, respiratory failure, and atelectasis. Obstructive sleep apnea is another common complication reported after SCI.

Orthostatic hypotension is defined as a drop of ≥10 mm of Hg and ≥20 mm of Hg, in systolic and diastolic pressure, respectively, when body position changes from supine to upright. This is due to reduced efferent sympathetic activity and loss of reflex vasoconstriction due to the SCI and has high prevalence in individuals with cervical injuries (Krassioukov, Eng, Warburton, & Teasell, 2009). Autonomic dysreflexia (AD) occurs in individuals with SCI at levels of T6 and above with higher incidence in

individuals with complete cervical injuries. The condition is caused by spinal reflex mechanisms initiated by a noxious stimuli entering the spinal cord below the level of injury. This afferent stimuli generates a sympathetic overactivity leading to vasoconstriction below the neurological lesion, along with involvement of splanchnic circulation that causes vasoconstriction and hypertension. Bladder distension and fecal impaction are the most common causes that trigger AD.

SCI causes neurogenic bladder as a result of areflexia or hyperreflexia of the detrusor and sphincter and may result in detrusor-sphincter dyssynergia, reflex or overflow incontinence, residual urine, or urinary retention (Benevento & Sipski, 2002). Neurogenic bowel affects nearly half (46.9%) of the SCI patients with higher level cord lesion, completeness, and time since injury as predictors of severity (Liu, et al., 2009). The condition is caused by colonic dysfunction resulting from lack of nervous control (Krassioukov, Eng, Claxton, Sakakibara, & Shum, 2010). Typically, individuals with tetraplegia suffer from upper motor neuron bowel syndrome due to lack of functioning abdominal musculature and impaired voluntary control of the external anal sphincter resulting in constipation and fecal retention (Stiens, Bergman, & Goetz, 1997).

Spasticity is a secondary complication associated with SCI affecting 70% of the patients (Gorgey, et al., 2010) (Rekand, Hagen, & Grønning, 2012). It is characterized by hypertonus, increased intermittent or sustained involuntary somatic reflexes (hyperreflexia), clonus and painful muscle spasms. Loss or reduced descending supraspinal inhibitory pathways are the likely cause (Rekand, Hagen, & Grønning, 2012).

Another common complication in SCI is neuropathic pain presenting above, at or below the level of lesion. In above the level neuropathic pain, the etiology is usually complex regional pain syndrome or compressive mononeuropathies while at the level of the injury pain may result from damaged nerve root or spinal cord. Finally, burning, aching, tingling, or stabbing sensation often occurs below the level of the injury, also called deafferentation pain (Siddall & Middleton, 2006).

Treatment options for individuals with SCI include surgery to restore hand function, medications to reduce side effects of the injury including pain and spasticity, rehabilitation including electrical stimulation, and assistive technologies.

In appropriate candidates, surgical interventions to restore elbow extension, wrist extension, reconstruction of grasp function and stabilization of the thumb may help in restoring function with the goal to improve independence with ADLs (Friden & Gohritz, 2015). Medications are often prescribed to manage the side effects of SCI like spasticity and pain. Antispastic agents like baclofen, benzodiazepines are prescribed to reduce hypertonicity and involuntary jerks of the muscles. Despite a heightened risk for side effects like dizziness, edema and somnolence, gabapentinoids like gabapentin and pregabalin are effective not only in treating neuropathic pain but also secondary conditions associated with SCI like anxiety, depression, and sleep interference (Mehta, McIntrye, Dijkers, Loh, & Teasell, 2014) (Davari, Amani, Amani, Khanijahani, & Akbarzadeh, 2020).

Physical and occupational therapies focus on rehabilitation and restoration of function after SCI. Restorative therapies not involving surgery include use of exoskeletons or gait orthosis to enable or assist with mobility. Electrical stimulation is another restorative therapy gaining traction. The approach may be non-invasive or invasive and broadly, fall into 2 major groups: functional electrical stimulation (FES) and transcutaneous or epidural SCS. The former targets motor axons of peripheral nerves while the latter targets either the dorsal column or the dorsal roots. FES is usually administered through self-adhesive electrodes placed over muscle motor points on the skin and an external stimulator (e.g., The Bionic Glove by Neuromotion, Edmonton, Canada; MyndMove Inc., Ontario, Canada). Implantable FES approaches work by electrodes placed either epimysially (Freehand system, NeuroControl, Cleveland, USA) or inside the muscle (Fesmate, NEC Medical Systems, Tokyo, Japan) and powered externally. FES and SCS studies have shown some promising results in hand and/or arm function improvement (Hoffman & Field-Fote, 2013) (Gad, et al., 2018).

SCI also results in a significant financial burden to the healthcare system. For individuals with high tetraplegia (C1-C4, AIS ABC), the annual costs for the first year and each subsequent year are $1,065,980 and $185,111, respectively. In a low tetraplegic (C5-C8, AIS ABC), the annual costs for the first year and each subsequent year are $ 770,264 and $113,557, respectively (National Spinal Cord Injury Statistical Center (NSCISC). Spinal Cord Injury Facts and Figures at a Glance. Available from: https://www. nscisc.uab.edu/Public/Facts%202016.pdf , 2016).

In summary, although most obvious, motor deficits comprise only one part in the vast spectrum of problems faced by individuals with SCI. Complications associated with this condition are significant and extend well beyond motor deficits as described in this section. Treatment options available for these individuals are limited and none so far offers a prognosis of durable fully restored function. Electrical stimulation may not only restore function but also improve other co-morbid conditions associated with SCI such as spasticity, sensory deficits, cardiovascular abnormalities, and autonomic issues, thereby improving the overall quality of life of these individuals.

3.5. Rationale for Using ARC Therapy in Spinal Cord Injury

In healthy individuals, muscle force generation is dictated by motor cortex excitability (Clark, Mahato, Nakazawa, Law, & Thomas, 2014). In cervical SCI resulting in tetraplegia, the descending tracts from cortex to spinal cord are damaged thereby limiting information transfer (Darian-Smith, Galea, & Draian-Smith, 1996). The end result is impaired hand muscle force generation resulting in weak power and precision of grip. Thus, augmenting the cortical signals reaching the spinal motoneurons with external stimulation may help restore hand/arm function. The inherent neuroplasticity of the central nervous system post-SCI may work synergistically with the applied external stimulation to achieve this outcome (Dietz & Fouad, 2014; Edgerton & Roy, 2012).

In nearly two-thirds of the 169 individuals with SCI implanted with an epidural SCS system, Waltz et al. demonstrated improvement in upper extremity motor function while reducing spasticity and improving bladder function (Waltz, Andreesen, & Hunt, 1987). Recently, in a case report of 2 individuals with motor incomplete SCI

implanted with cervical SCS systems, clinically significant improvement in upper extremity motor scores and grip strength were reported (Lu, et al., 2016). In another report, application of transcutaneous electrical stimulation resulting in improved upper extremity function was observed (Inanici, et al., 2018). Thus, irrespective of stimulation modality, it appears that targeting the dorsal column/root may promote neuroplasticity that is clinically meaningful.

3.6. Prior Clinical Experience with the LIFT System

Beginning in May of 2017, a series of investigator-sponsored, small-scale clinical studies using ARC Therapy administered by the LIFT System were undertaken, some of which still are ongoing. Studies were conducted at the following seven (7) sites:

1. Strides SCI Functional Fitness (San Juan Capistrano, CA)
2. Rancho Los Amigos National Rehabilitation Center (Downey, CA)
3. University of Washington (Seattle, WA)
4. Queen Elizabeth Spinal Unit (Glasgow, Scotland, UK)
5. Neurokinex Rehab Centers (London, UK)
6. Craig Hospital (Englewood, CO)
7. Kessler Rehabilitation Center (West Orange, NJ)

Under IRB or ethics committee approval and after obtaining informed consent, a total of 52 individuals were enrolled in a total of eight (8) studies. Individuals with total or partial loss of motor and/or sensory function due to spinal cord injury participated in these initial studies. To a varying degree, each study enrolled subjects with different sites of injury ranging in classification and age from AIS A to AIS D and 18-66 years old, respectively. Subjects were 1-23 years post-injury. Each subject was involved in a type of activity-based training along with ARC Therapy administered by the LIFT System for at least 2 months on average.

Clinically meaningful gains in motor and sensory function were assessed using a combination of metrics derived from established neurological and physical medicine rehabilitation practices. Gains include recovery or improvement in voluntary control of the lower and upper extremities, hand, trunk control, cardiovascular function, thermoregulation, independent standing, activities of daily living (ADLs), and quality of life. Over the course of these studies, a set of best practices have emerged which has formed the basis of the proposed pivotal clinical study.

Historically, the focus of ARC Therapy had been to improve or restore voluntary movement in the lower extremities of people with SCI with encouraging results. Equally important, if not more, is improved hand and arm function that promotes independence with ADLs such as feeding, bathing, dressing, and bladder/bowel management among other activities. Therefore, these 8 studies were focused on different objectives including both upper and lower extremity recovery.

The following table lists all clinical studies performed through a cutoff date of 1st of May 2020 using ARC Therapy administered by the LIFT System (Table 1) arranged chronologically by start date. These studies were single arm feasibility studies in SCI subjects in which proof of concept and preliminary safety and effectiveness of ARC Therapy with LIFT in treating SCI were evaluated in various protocols.

Study ID	Start Date	No. of Subjects	Study Site	Injury Site	Area of Focus
1	May 2017	13	Strides	C3-C7<br>C4-T11	Restore hand function, standing, stepping
2	Aug 2017	7	University of Washington	C3-C5	Restore hand function
3	Oct 2017	7	Rancho Los Amigos	T11 and above	Restore bladder function
4	Mar 2018	4	University of Washington	C3-C7	Locomotion & autonomic function
5	Jun 2018	4	Queen Elizabeth	C2-C6	Determine safety & efficacy
6	Jul 2018<br>Nov 2019	2<br>6	Neurokinex	C4<br>T6-T12	Determine safety & efficacy, restore hand function, standing, stepping
7	Jul 2018	7	Craig	C5-C6	Restore hand function, standing, stepping, Neurorecovery Network Protocol
8	Jul 2018	2	Kessler	C5-C8	Restore hand function, standing, stepping
**Total**		**52**	**7**	**C2-C8, T5-12**	

Table 1. List of feasibility studies using the LIFT device.

Four of these feasibility studies (study IDs 1, 2, 7 and 8) included 20 patients who followed a treatment protocol focused on upper extremity recovery that is similar to what is planned for the proposed pivotal study. Although there were differences in the study protocols, the data from this subset represents a generally homogenous data set with outcomes that are suggestive of the results expected from the proposed pivotal study.

The aim of these studies was threefold: 1) test run a study design that will be used as a basis for the pivotal study; 2) identify the optimum endpoints to target; and 3) investigate the effectiveness of ARC Therapy with LIFT to improve hand and arm function in individuals with significant deficit due to chronic cervical SCI.

After a period of baseline assessment, the upper extremity studies included a sequential design of alternating blocks of intervention. For example, one month of exercise therapy alone was followed by one month of exercise therapy plus stimulation; typically, 3 sessions per week for 1-2 hours per session. In some cases, patients received continued therapy in a randomization scheme over an additional period of time. Functional testing at prescribed intervals was used to assess treatment effects and safety.

Each individual enrolled underwent a medical evaluation including International Standards for Neurological Classification of Spinal Cord Injury (ISNCSCI) to confirm the level of injury and to determine eligibility for participation based on IRB-approved inclusion/exclusion criteria. Once an individual was determined to be eligible, he/she underwent neurophysiologic testing (spinal motor evoked potentials recorded with

surface EMG) with the LIFT system, to assess if their neurological state would be responsive to stimulation therapy and what their threshold potential may be. If the neurophysiologic testing was positive (which was performed in only select sites), the individual was allowed to proceed. Prior to the start of the therapy, individuals also underwent multiple tests such as pinch and grasp force measurements using force transducers, GRASSP, CUE-Q/T and completed questionnaires such as SCIM III, The Neurogenic Bowel Questionnaire, The Neurogenic Bladder Symptom Score, Patient Health Questionnaires (PHQ-9), and SF-Qualiveen Instrument.

The exercise training program was comprised of functional task practice that included repetitive activities of gross upper extremity movement, isolated finger movements, simple and complex pinch, and grasp performance measures. For each category, 8-10 activities with varying levels of difficulty were assigned to the subjects such that each subject performed 1-2 activities within the same category during each treatment session. Activities in each category were chosen according to the subjects' ability and were modified as the ability to perform the functional task progressed over time. Typical movement patterns were encouraged by guidance and feedback. When a subject had little to no voluntary movement, active assistance from a physical or occupational therapist was provided to model the desired activity.

ARC Therapy was administered by the LIFT System. Biphasic or monophasic 1 ms pulses at a frequency of 15-30 Hz modulated by an overlapping high frequency of 10 kHz were used. Stimulation intensity ranged from 5-100 mA, based on the threshold to evoke motor response and also according to the subjects' comfort level. Stimulation was delivered via commercially available 1.25" round electrodes as cathodes and two 1.5" x 3.5" rectangular electrodes as anodes (Axelgaard Manufacturing Co. Ltd., USA) or equivalent. Cathode electrodes were placed midline on the skin of the neck, one above and one below the injury site, using inion and spinous processes as landmarks. Anode electrodes were placed symmetrically parallel to the iliac crests. Stimulation was applied intermittently for up to 90 minutes during the session simultaneous with exercise therapy.

The demographics of patients enrolled in the four upper extremity studies are described in Table 2. Upper Extremity Study Demographics. In aggregate, the demographics of those enrolled were 80% male, age 34.4±14.1 yr., post-chronic non-progressive cervical injury 5±5.3 yr., classified as AIS-A though AIS-D.

The results of the studies demonstrated performance gains in each individual enrolled with improvement in one or more of the various outcome measures that were tested. In addition, new functional gains were also noted such as the ability to pick up and hold objects, manage the use of an object like a utensil, or perform a new task like play a musical instrument.

Study ID	N	% Male	Age in Yr. (Range)	AIS	Injury Level	Time Since Injury in Yr. (Range)	Study Duration in Mo
1	5	100	34.8 (25-61)	B (1), C (4)	C3-C7	7.8 (1.5-23)	2
2	7	71	40.6 (28-62)	B (3), C (2), D (2)	C3-C5	4.3 (1.5-12)	4
7	7	71	27.0 (18-55)	A (2), B (2), C (2), D (1)	C5-C6	1.9 (1-4)	1-4
8	1	100	39.0	A (1)	C5	15.0	12
**Total**	**20**	**80**	**34.4 (18-62)**	**A (3), B (6), C (8), D (3)**	**C3-C7**	**5.0 (1-23)**	**3.3 (1-12)**

Table 2. Demographics of upper extremity SCI subjects enrolled in the LIFT feasibility studies.

Study ID: 1
Title: Non-invasive Electrical Spinal Cord Stimulation to Improve Motor Function and Functional Outcomes in Individuals with Paralysis
PI: Sujin Lee MD
Site: Strides SCI Functional Fitness Rehab Center, California
Publication: Pending

This study was initiated in May of 2017 at Strides SCI Functional Fitness Rehabilitation Center, San Juan Capistrano, CA to evaluate the benefits of ARC Therapy in promoting long-term neuroplasticity and recovery of hand and arm function in individuals living with chronic paralysis. Five patients (all male, mean age 34.8 yrs.) with both motor complete (N=1) and incomplete (N=4) cervical SCI were screened and enrolled in the upper extremity phase of the study. Four completed the study with one withdrawing prior to final assessment to participate in another study. Each participant was subjected to task-specific OT followed by OT and ARC Therapy with LIFT over a 2-month period. Improvements in pinch force, hand strength, and prehension performance were noted in some with training alone though greater gains were realized once ARC Therapy was applied. This confirmed the need to include a period of training alone at the start of any study to ensure an appropriate pre-intervention baseline state. Remarkably, one study subject, whose injury was 23 years ago (ASIA C, injury level C5), demonstrated an increased ability to pick-up small objects with only a 4-5-point increase in GRASSP-Strength. Another subject (ASIA B, 5 years post-injury, injury level C7) demonstrated a 17% increase in grip strength in his left hand and a 50% increase in his right hand with a 4-point increase in GRASSP-Prehension after OT and ARC Therapy with LIFT. This markedly improved hand function was characterized by resolution of his tenodesis.

In addition to the results described above for upper extremities in this study, eight subjects (mean age: 36.3 yrs.; 62% male) with both motor complete (N=4) and incomplete (N=4) SCI were enrolled in a second phase of the study that included

standing and stepping. The objective of this study phase was to evaluate the effects of ARC Therapy with LIFT in restoring lower extremity function, along with assessing human factors for the application, comfort, and safe use of the device. This study is ongoing, having so far demonstrated the safety and tolerability of ARC Therapy with LIFT. Preliminary functional outcomes for improved ability to stand with better trunk control and stability, along with improved endurance, and the ability to step were achieved. Functional gains after discharge were also noted. Complete outcomes will be reported at the conclusion of the study.

**Study ID:**	2
**Title:**	Non-invasive spinal cord stimulation restores hand and arm function after paralysis
**PI:**	Fatma Inanici MD PhD, Lorie Brighton PT, Soshi Samejima MS DPT, Chet Moritz PhD
**Site:**	University of Washington, Seattle, Washington
**Publication**:	In press

This study was initiated in August of 2017 at The University of Washington, Seattle to evaluate the benefits of ARC Therapy with LIFT to promote long-term neuroplasticity and recovery of hand and arm function in patients with cervical motor complete or incomplete SCI. Seven patients were screened and enrolled with six completing the study (4 men, 2 women; mean age: 40.6 yrs.); one withdrew due to scheduling conflicts. Subjects initially received one month of upper extremity exercise training alone, followed by ARC Therapy with LIFT paired with the same training. Two subjects with motor complete paralysis had no voluntary movement of their fingers or thumbs on at least one hand at baseline and remained paralyzed after four weeks of training alone. After four subsequent weeks of training paired with ARC, they were able to move their fingers and thumbs and produce measurable pinch force for the first time since injury. The remaining four subjects showed similar improvement.

As measured by the GRASSP score, improved hand function was significantly higher after stimulation compared to training alone for pinch force, arm and hand strength, and dexterity ($p<0.025$, paired-sample t-test). Pinch force improved significantly from 2.4- to 4.8-fold during stimulation combined with training compared to baseline levels ($F(1.1,5.3)=8.8$, $p=0.029$). One-way repeated ANOVA measures also confirmed significant improvement in GRASSP test measures for strength ($F(2,10)=18.0$, $p<0.001$) and quantitative prehension ($F(2,10)=49.3$, $p<0.001$). Post-hoc comparisons showed that all measures were significantly greater at the end of stimulation than after training alone, whereas the changes with training alone were not significant (except for GRASSP strength) from baseline. Importantly, all functional improvements were sustained after three to six months of follow up; all measures were significantly greater ($p<0.05$) at the final follow-up visit compared to baseline.

The upper extremity motor scores (ISNSCI) of all subjects improved by up to eight points at the end of stimulation paired with training compared to two points or less following training alone. Additional benefits included reduction in spasticity, normalized heart rate, and improved bladder function for several participants. Several

subjects improved to such a degree that they were able to resume complex hobbies such as oil painting and playing the guitar for the first time in up to 12 years after injury.

Study ID: 7
Title: Transcutaneous Spinal Cord Stimulation in Combination with Massed Practice Training in Spinal Cord Injury
PI: Candy Tefertiller PT, DPT, PhD, NCS
Site: Craig Hospital, Denver, Colorado
Publication: Pending

This study was initiated in July 2018 at Craig Hospital, Englewood, Colorado to evaluate the feasibility and safety of ARC Therapy with LIFT in a clinical setting to promote further neurological recovery in individuals with chronic SCI. IRB approval was obtained to enroll 10 individuals who had previously completed the Neurorecovery Network (NRN) rehabilitation program. The study entailed the repetition of the prescribed NRN therapy program with the addition of concomitant ARC Therapy administered by the LIFT System.

Individuals enrolled had previously completed at least 40 sessions of the NRN program consisting of 20 sessions of upper extremity and 20 sessions of lower extremity training. Each had been discharged from outpatient therapy due to a plateau in progress occurring at least 3 months prior to enrolment in the study.

This study is ongoing with 7 subjects enrolled to date; mean age of 27 years; 71% male with both motor complete (N=4) and motor incomplete (N=3) SCIs. The study protocol stipulates 1.5 hours of massed practice training in combination with ARC Therapy with LIFT for upper extremity function and 1.5 hours of massed practice training with ARC Therapy with LIFT for lower extremity function per day for 5 days each week for 4-16 weeks, for a total of 20-80 sessions. Anecdotally, each subject has been able to achieve further progress with the addition of stimulation therapy compared to what was achieved during application of the otherwise extensive NRN protocols. Clinically meaningful improvement in hand and arm function for the upper extremity appears to have resulted from the combined therapy, with some individuals showing a change in upper extremity motor score (change of 1-8 points). Formal outcomes will be reported at the conclusion of the study.

Study ID: 8
Title: Non-Invasive Electrical Spinal Cord Stimulation to Improve Motor functions and Functional Outcomes in Individuals with Paralysis
PI: Gail Forrest PhD
Site: Kessler Rehabilitation Center, West Orange, New Jersey
Publication: Pending

This study was initiated in July 2018, at Kessler Rehabilitation Institute, West Orange, NJ to evaluate the benefits of ARC Therapy with LIFT in promoting long-term neuroplasticity and recovery of upper and lower extremity function in individuals living with chronic paralysis. Two subjects have been enrolled to date: Subject#1, a 39 y/o male AIS A who is 15 years post-injury for assessing upper extremity recovery; and subject#2 a 49 y/o female AIS D 4 years post-injury to assess lower extremity

functional recovery. Positive outcomes have been noted thus far with subject#1 demonstrating increased hand strength and subject#2 demonstrating the ability to stand with minimal assistance and take steps with the use of a walking cane. Formal outcomes will be reported at the conclusion of the study.

3.7. List of AEs and SAEs Reported in the Feasibility Studies Using the LIFT System

A listing of all reported AEs and SAEs in the feasibility studies using the LIFT System are presented in Table 3. Listing of all AEs and SAEs reported in the feasibility studies and their sequelae are listed below.

#	IRB # Subject #	Site	Mo/Yr.	Type	Severity	Device Related	Description
1	TSCS-07 #995633	Strides	Dec 2017	SAE	Severe	No	Autonomic dysreflexia
2	TSCS-07 #582202	Strides	Dec 2018	SAE	Moderate	No	Syrinx formation
3	RNI00000205 Subject #5	U Washington	Mar 2018	AE	Minor	No	Deep vein thrombosis
4	RNI00000642 Subject #9	U Washington	May 2019	AE	Minor	No	Urinary tract infection
5	RNI00000703 Subject #2	U Washington	Aug 2019	AE	Severe	No	Fall
6	RNI00000562 Subject #7	U Washington	Jan 2020	AE	Minor	Likely	Skin rash
7	HRA 254950 #G3	Neurokinex	Feb 2020	AE	Minor	Yes	Neuropathic pain

Table 3. Listing of all AEs and SAEs reported in the feasibility studies using LIFT device.

1: Patient experienced an episode of autonomic dysreflexia while warming up for therapy session requiring emergency first aid and hospitalization to manage BP. The adverse event was considered serious. Problem resolved and patient was discharged after several days of treatment and observation.

2: Patient developed syrinx after completion of study. The adverse event was considered serious. Early signs of motor impairment were noted; patient was seen by his neurosurgeon, syrinx was addressed; lost to follow-up.

3: Patient developed deep vein thrombosis after baseline assessment, though prior to training or stimulation. Patient was referred to her private physician for care. Patient was allowed to return to the study after a successful course of treatment on an outpatient basis.

4: Patient developed a urinary tract infection with kidney stones post-baseline, prior to testing. Treated and released.

5: Fall accident during gait training w/o stimulation sustaining a nose fracture; subject was treated, released, and eventually cleared to return to the study.

6: Patient developed a rash on his wrist and forearms after receiving stimulation; family history of hives; possible attribute to normalization of autonomic nervous system function. Local topical therapy was applied, with selfcare and monitoring condition resolved.

7: Patient experienced neuropathic pain during stimulation with training; managed by reducing level of intensity and exposure to stimulation.

In summary, none of the SAEs were deemed device related.

3.8. Summary

Initial clinical experience with the LIFT System has demonstrated safety and efficacy across multiple studies and investigators.

4. INTENDED USE AND DEVICE DESCRIPTION

4.1. Intended Use

The LIFT System is intended to be used to improve or restore upper extremity sensory and motor function in people with chronic neurological deficit resulting from incomplete spinal cord injury. The LIFT System is intended to be used in conjunction with physical or occupational therapy at the hospital and at a rehabilitation therapy clinic.

4.2. Device Description

The functionality of the LIFT System is based on the principle that neuromodulating the spinal circuitry with transcutaneous or noninvasive electrical spinal stimulation enables individuals with paralysis to process proprioceptive, auditory, and visual input to regain voluntary control of paralyzed muscles. ARC Therapy enables voluntary movement by providing a subthreshold stimulus to viable but electrically dormant neurons to re-engage those neurons allowing the strengthening of motor and sensory pathways with intensive training.

In a clinical setting (e.g., hospital, physician's office, or rehabilitation clinic), the subject's physician, therapist, or other qualified medical personnel authorized to prescribe the therapy, will program the stimulation therapy parameters based on the specific needs of the individual and therapeutic strategy. The hydrogel electrodes are affixed to the subject at specified locations overlying the spinal cord using anatomical landmarks, then the electrodes are connected to the stimulator's electrode cable assembly. The choice of stimulation parameters is dictated by threshold required to induce a stimulation-evoked spinal response that is recorded by surface electromyogram (EMG) electrodes or based on the motor response evoked in the upper extremity muscles. Up to 5 programs may be programmed at one time based on the threshold stimulation parameters. During an initial assessment of programmed settings, the user has the ability to change the stimulation parameters, such as current amplitude, at any time while therapy is being delivered. Also, the therapy can be stopped at any time.

In an ambulatory setting (e.g., at home), the rehabilitation therapist, qualified medical assistant, or caregiver will affix the hydrogel electrodes and connect the electrode leads to the stimulator's electrode cable assembly as before. While the stimulator is turned on, the user will be able to select one of the specific programs previously set up by the clinical staff and initiate stimulation therapy by pressing the stimulation button. At any time during the delivery of therapy by the stimulator, the therapist, assistant, subject, or caregiver will be able to turn the stimulator off, as well as increase or decrease the current amplitude within a narrowly defined range that is set by the clinical staff based on threshold testing. (Home use will be evaluated in a subsequent study.)

The subject interface shall allow the initiation or termination of therapy, selection of a stored therapy program, and adjustment of the stimulator current to ±10% of the setpoint so long as the maximum current setting is not exceeded. The stimulator shall create a log of events during therapy delivery. The therapy delivery logs shall be stored in the stimulator and shall be retained after power has been removed or the battery is fully depleted. A minimum of 180 therapy session logs shall be stored on the device.

Generic name: Transcutaneous Spinal Cord Neurostimulator
Type of therapy: Noninvasive Electrical Spinal Stimulation
Model name: LIFT
Model number: Beta
Name and address of legal manufacturer:

ONWARD Medical, Inc.
7 Carmel Circle
Lexington, MA 02421

Manufacturing performed on behalf of ONWARD Medical, Inc. by:
Minnetronix Medical, Inc. (an Original Equipment Manufacturer holding ISO 13485 and 9001 Certification)
1635 Energy Park Drive
St. Paul, MN 55108

Classification: Not classified

The LIFT Stimulator System is being developed to meet the requirements of 21 CFR 820.30 and ISO 13485:2016. The data development plan includes non-clinical bench testing and clinical studies. The approval request for the device will demonstrate that the requirements for the following international standards have been met:

- IEC 60601-1 2005: Amd 2012: Medical Electrical Equipment – Part 1: General requirements for basic safety and essential performance
- IEC 60601-1-2 2014: Medical Electrical Equipment – Part 1-2: General requirements for basic safety and essential performance – Collateral Standard: Electromagnetic disturbances – Requirements and tests
- IEC 60601-1-6 2010: Amd 2013: Medical Electrical Equipment – General requirements for basic safety and essential performance – Collateral Standard: Usability

- IEC 60601-1-8 2006: Amd 2012: Medical Electrical Equipment – General requirements for basic safety and essential performance – Collateral Standard: General requirements, tests, and guidance for alarm systems in medical electrical equipment and medical electrical systems
- IEC 60601-2-10 2012: Amd 2016: Medical Electrical Equipment – Part 2-10: Particular requirements for the basic safety and essential performance of nerve and muscle stimulators
- IEC 62304 2006: Amd 2015: Medical device software – Software life-cycle processes
- IEC 62366-1 2015: Medical devices – Application of usability engineering to medical devices
- ANSI/AAAMI/ISO 10993-1: 2009/(R)2013: Biological evaluation of medical devices – Part 1: Evaluation and testing within a risk management process
- ISO 14155 2011: Clinical investigation of medical devices for human subjects – Good clinical practice
- IEC 60601-1-11 2015 Home Healthcare

Testing has been performed to establish the safe use in controlled clinical environments by Medical Equipment Compliance Associates, LLC. Testing was performed per the following applicable standards: IEC 60601-1 (2005: Amd 2012) and IEC 60601-2-10 (2010: Amd 2013). The evaluation for clinical trial use included the following testing and essential performance requirements from the specified standards: Determination of Accessible Parts (IEC 60601-1 Clause 5.9.2, Touch and Patient Leakage Current (IEC 60601-1 Clause 8.7), Dielectric Voltage Withstand (IEC 60601-1 Clause 8.8.3), Temperature of Accessible and Applied Parts (IEC 60601-1 Clause 11.1.1), and Limitation of Output Parameters (IEC 60601-2-10 Clause 201.12.4.104). In summary, Medical Equipment Compliance Associates found the testing of the LIFT Stimulator System to be in compliance with the above standards.

4.2.1. Components and Parameters

The LIFT System is composed of a Stimulator, Battery Charger, Electrodes, Programmer, Connecting Cables, and accessories (Figure 2). A description of the system is provided below:

- Stimulator – The Stimulator is a 4-channel device that delivers a mono- or bi-phasic charge balanced waveform at constant current, pulsed stimulation through electrodes placed on the skin (Table 4). Each channel is independent and intended to be connected to a separate pair of electrodes. The Stimulator delivers current through the channel output and uses a separate electrode as the return. Each channel may be configured independently with a unique current level, frequency, waveform, and various timing parameters. A program may be defined that specifies the length of time therapy is delivered and the preconfigured channels for a therapy session. The Stimulator is powered by an internal rechargeable battery. The Stimulator has an LCD display screen, functional touch sensitive buttons, LED and audible indicators, battery charger connector port, and a removable electrode cable assembly.

- Battery Charger – The Battery Charger is a direct wired charger that recharges the Stimulator batteries through the charger connector port.

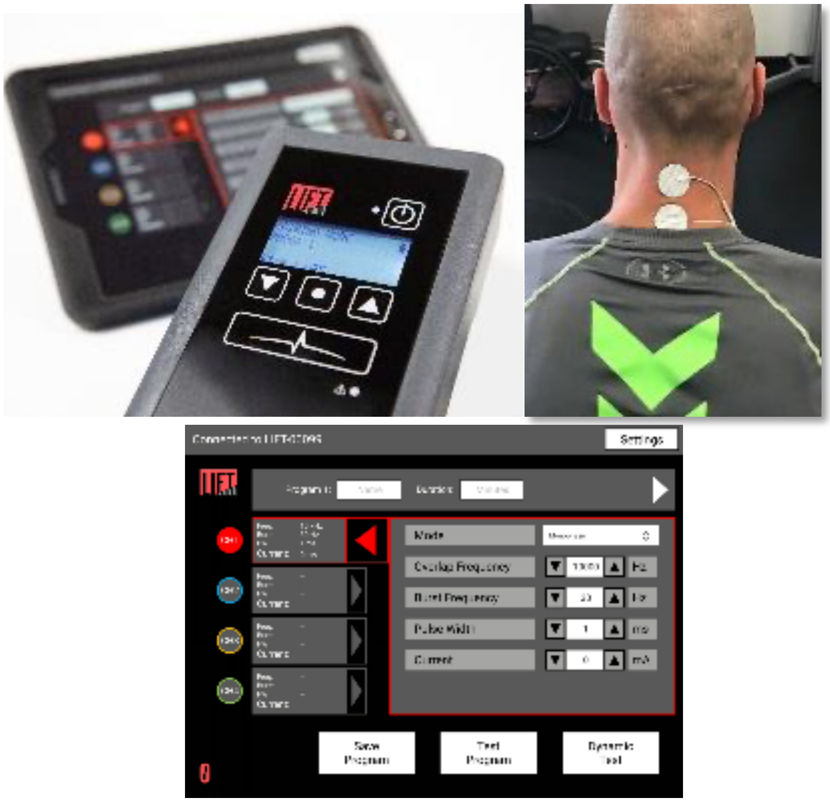

Figure 2. The LIFT System components (top left) when used for non-invasive electrical spinal stimulation at the cervical level (top right). A close-up of the tablet display of the programmer (bottom).

- Electrodes – Commercially available hydrogel-based electrodes are affixed to the subject’s skin midline over the spine using spinous processes as landmarks and are connected to the Stimulator’s electrode cable assembly. Stimulation is typically delivered using 1.25” round electrodes as cathodes and two 1.5” x 3.5” rectangular electrodes as anodes or equivalent. Cathode electrodes are placed midline on the skin of the neck, often placing one above and one below the injury site. Additional cathode electrodes may also be placed at other vertebral positions. e.g., at the thoracic level to aid with trunk stability. Anode electrodes are placed symmetrically parallel to the iliac crests. Electrodes of any size can serve as cathodes and anodes so long as they do not violate the charge density specifications associated with skin damage/burns.
- Programmer – The Programmer is an off the shelf Android tablet-based computer with proprietary software used by physicians and clinical personnel that wirelessly communicates with and programs the Stimulator. The wireless communication between the stimulator and the programmer utilizes the Bluetooth Low Energy protocol. The Programmer allows the Stimulator to be

configured with programs that deliver therapy to the subject. The Programmer has the capability to retrieve device log file data.

- Connecting Cables – The connecting cables transmit the stimulation energy to single-use hydrogel electrodes affixed to the subject's skin.

	Parameter	Stimulator Specification
Treatment Modes	Functional Programs	5 Programs
Stimulation Output Parameters	Quantity of Output Channels	4
	Output Waveform	Monophasic, Biphasic, Rectified
	Current Amplitude	0.0 mA – 300 mA
	Burst Frequency	0.2 Hz – 100 Hz
	Pulse Width	0.5 ms – 5 ms
	Overlapping Frequency	5 kHz – 10 kHz
	Therapy Duration	1 min – 480 min
General Specifications	Stimulator Power Supply	2600 mAh, 7.26 V, 19 Wh
	Stimulator IP Classification	IP22
	Stimulator Size	8.11" x 4.33" x 1.85"
	Stimulator Weight	19 ounces
Operating Conditions	Temperature	5° C to 40° C
	Relative Humidity	15% to 90% non-condensing
	Atmospheric Pressure	700 hPa to 1060 hPa
Storage Conditions	Temperature	-18° C to 55° C

Table 4. Parameters of the LIFT stimulator.

5. STUDY PURPOSE AND OBJECTIVES

5.1. Study Purpose

The purpose of this pivotal study is to evaluate the safety and effectiveness of non-invasive electrical spinal stimulation (ARC Therapy) therapy administered by the LIFT System (ONWARD Medical, Inc., Lexington, MA, USA, henceforth referred as "The Sponsor") for restoration of upper extremity function in individuals with chronic tetraplegia when compared to rehabilitation therapy, the current standard of care. Additionally, prospective data on quality of life and autonomic function improvements will be captured as secondary and/or observational endpoints.

5.2. Study Objectives

5.2.1. Safety

To provide confirmatory evidence that use of the LIFT System, inclusive of all components and accessories, is safe.

5.2.2. Effectiveness

To provide confirmatory evidence that use of the LIFT System provides an effective treatment for the restoration or improvement in upper extremity strength and function.

5.2.3. Other

To provide data regarding the potential benefits of the LIFT System to achieve other secondary outcomes such as improvement in pain, spasticity, quality of life, cardiovascular (blood pressure) and autonomic function.

5.3. Standard of Care: Functional Task Practice

Individuals with tetraplegia undergo occupational therapy with the goal of improving upper extremity strength and function, trunk control, lower extremity strength and function, bladder catheterization to name a few. This study will uniquely offer training to improve upper extremity strength and function through movements and motor strategies that comprise common everyday activities called "functional task practice (FTP) " as a primary therapeutic factor (Gomes-Osman, Tibbett, Poe, & Field-Fote, 2017). The use of FTP best describes the therapy offered to the study subjects. The authors reported that with bimanual FTP, when offered with repetitive peripheral nerve stimulation (PNS), significant improvements in precision grip force were observed in both the stronger and weaker hand in the FTP + PNS group (effect size: 0.51, p = 0.04 and 0.54, p = 0.03, respectively). This technique of offering FTP with somatosensory stimulation was further validated in a randomized controlled study demonstrating meaningful improvement in function, strength, and sensory testing (Beekhuizen & Field-Fote, Sensory Stimulation Augments the Effects of Massed Practice Training in Persons With Tetraplegia, 2008). These findings support the use of FTP in incomplete SCI subjects as an effective control to compare any new therapies.

6. STUDY ENDPOINTS

6.1. Rationale for the Selection of Study Endpoints

The choice of study endpoints for this pivotal study was guided by multiple factors:

1. Safety,
2. Relevance to UE function,
3. Capture improvements in both strength and function, and
4. Magnitude of changes that are clinically meaningful.

To align on specific outcome variables, the recommendations for SCI common data elements (CDE) proposed by National Institute of Neurological Disorders and Stroke (NINDS) were reviewed. The SCI CDE Working Group is supported by the NINDS CDE Team. This group recommended standardized, validated instruments for SCI research. The International Spinal Cord Society (ISCoS) and the American Spinal Injury Association (ASIA) have since collaborated to incorporate the International SCI data sets into the NINDS CDEs. Next, the findings from stakeholder groups like North American Spinal Cord Injury Consortium (NASCIC), Praxis Spinal Cord Institute and Neurotech Network were reviewed to identify the priorities of those suffering from the condition. Lastly, discussions with thought leaders in SCI research were also solicited. Based on these inputs, it was determined that a single composite primary endpoint that includes ISNCSCI, GRASSP, CUE-T, pinch and grasp strengths would be ideal to capture hand/arm improvements in both strength and function dimensions. To get a

complete picture of the improvements with ARC Therapy, additional measures that reflect functional recovery, quality of life, and autonomic function will be captured as secondary and observational endpoints as reported in the following sections.

In the absence of relevant prior data on minimum detectable difference (MDD) or minimal clinically important difference (MCID) in this specific, chronic SCI population, an alternative approach is to use the effect size as described by Cohen (Cohen, 1988).

Cohen's effect size, ! = !"#!$ / %('"!('$!)

where,

m1, m2 = Means of two independent samples

$\alpha 1$, $\alpha 2$ = Standard deviations of the respective populations

Cohen's effect size is a measure of difference in means between the test and the control populations. It measures the degree to which a phenomenon exists in the population or is induced by the treatment. It can also quantify the degree to which the null hypothesis is false. The larger the effect size, the greater the certainty with which the null hypothesis can be rejected and more clinically relevant the outcome. Small, medium, and large effect sizes were defined as 0.2, 0.5 and 0.8, respectively.

In a comprehensive review, Wu et al. discussed the limitations of not being able to use MDD/MCID in the chronic SCI population and instead suggest that Cohen's effect size (d) is a better statistic to use (Wu, et al., 2015). Using this method, one can calculate the minimal important difference (MID) for each metric within the primary endpoint resulting from exposure to ARC Therapy:

$$\text{MID} = d \; * \; \sqrt{(\sigma 1^2 + \sigma 2^2) / 2}$$

This definition was applied for a small effect size (d=0.2) in a small but representative sample of feasibility study data collected in up to 13 subjects from across 3 sites to define a responder for the various primary outcome measures listed in Table 5. To be deemed a responder, a subject has to meet or exceed the values listed below for the respective outcomes.

Outcome	MID
**ISNCSCI-UEMS**	2-point change
**GRASSP-Strength**	4-point change
**GRASSP-Prehension**	2-pt change
**Pinch force**	≥2.4 N
**Grasp force**	≥6 N
**CUE-T**	4-point change

Table 5. MID definition based on pilot study data for primary endpoint outcomes.

It is understood that calculating MID using this approach has two major limitations:

1) Treatments offered to subjects (both FTP and ARC Therapy) differed somewhat with different outcome data collected at each site.
2) The pilot data had a small sample size with large variations. Thus, it is likely that the MID values may be artificially inflated.

The former issue will be addressed in the consistent implementation of a uniform pivotal study protocol across all sites. The latter is addressed through a pre-defined sensitivity analysis as described in Section 14.8.2.

6.2. Primary Endpoint

6.2.1. Safety

Observational data regarding the incidence of serious adverse events (SAEs) related to the use of the study device and treatment procedures will be reported.

6.2.2. Effectiveness

The primary effectiveness outcome measure will test the hypothesis that a majority of the subjects will experience clinically significant improvement in selected strength and functional performance metrics after treatment with ARC Therapy administered by the LIFT System and FTP. A subject will be considered a treatment responder if she/he reports clinically relevant improvements in at least one outcome each of the Strength and Function domains as follows in Table 6.

Strength		Function
ISNCSCI-UEMS		GRASSP-Prehension
GRASSP-Strength	and	CUE-T
Pinch force		
Grasp force		

Table 6. List of outcomes to assess improvements in upper extremity strength and function at primary endpoint.

6.3. Secondary Endpoints

Safety: All AEs and SAEs in the study will be reported. This includes device and procedure related adverse events experienced during the study pursuant to the reporting guidelines stipulated in Section 13 Safety Definitions and Reporting Requirements.

Effectiveness: To capture meaningful improvements in established outcomes assessing upper extremity function, the following hierarchical testing will be carried out. These endpoints will be tested in descending order of importance through hierarchical testing as described in the Statistical Analysis Plan (SAP).

- Superiority of combined FTP and ARC Therapy with LIFT vs. FTP alone as described by statistically significant difference in responder rates (comparison of change from enrollment baseline to end of FTP with the change from enrollment baseline to end of combined FTP and ARC Therapy with LIFT)
- Quantitative comparison of individual performance metrics to establish superiority of FTP and ARC Therapy with LIFT compared to FTP alone:
  - Pinch force
  - GRASSP-Prehension
  - GRASSP-Strength
  - ISNCSCI-UEMS
  - ISNCSCI-Total sensory scoreEQ-5D-5L
  - SCIM
  - WHOQOL-BREF

6.4. Observational Endpoints

Descriptive statistics on additional domains within the primary endpoint outcomes will be reported. The following additional assessments impacting individuals with SCI such as outcomes related to pain, spasticity, quality of life, sleep and bladder/bowel/sexual function will be reported as descriptive statistics and clinically relevant changes, where possible.

- Numerical Rating Scale (NRS) for pain
- International Spinal Cord Injury Pain Data Set (ISCIPDS)
- Medical Outcomes Study (MOS) Sleep Scale
- Spinal Cord Independent Measure (SCIM III)
- Penn Spasm Frequency Scale (PSFS)
- EuroQol 5 Dimension 5 Level Questionnaire (EQ-5D-5L)
- World Health Organization Quality of Life (WHOQOL-BREF)
- International Standards to document remaining Autonomic Function after Spinal Cord Injury (ISAFSCI)
- Patient Health Questionnaire (PHQ-9)
- Global Impression of Change (Clinician and Patient)

An optional follow-up assessment performed from 1-30 days after completion of the study will be reported to allow for evaluation of the immediate neuroprosthetic effect of active stimulation on strength and motor function.

6.5. Rationale for the Selection of Observational Safety Data Reporting

The LIFT System provides a form of non-invasive stimulation therapy. Neuromuscular electrical stimulation (NMES), a neuromuscular analog of ARC Therapy, has been in use for several decades with a well-established safety profile. Most adverse events are transient and mild-to-moderate in severity and spontaneously resolve upon cessation of stimulation. Moreover, studies where ARC Therapy was administered using the

LIFT System, both unpublished and published in peer reviewed journals, report a low rate of device-related SAEs as reported in Section 3.7. Since the number of subjects participating in these studies are small, the best indicator for SAE incidence is per stimulation session. In the 20 UE subjects followed-up for an average of 3.3 months, conservatively assuming 8 sessions/month, these subjects received a minimum of 528 stimulation sessions with no device related SAEs reported. In 2019, FDA determined that the study titled “Transcutaneous Spinal Stimulation in Patients with Cervical Spinal Cord Injury" is a nonsignificant risk (NSR) device study because it did not meet the definition of a significant risk (SR) device under 21 CFR 812.3(m) of the investigational device exemption (IDE) regulation (21 CFR 812) (FDA, 2006). A device is deemed significant risk if it meets one of the following four criteria-

1. Is intended as an implant and presents a potential for serious risk to the health, safety, or welfare of a subject;
2. Is purported or represented to be for a use in supporting or sustaining human life and presents a potential for serious risk to the health, safety, or welfare of a subject;
3. Is for a use of substantial importance in diagnosing, curing, mitigating, or treating disease, or otherwise preventing impairment of human health and presents a potential for serious risk to the health, safety, or welfare of a subject; or
4. Otherwise presents a potential for serious risk to the health, safety, or welfare of a subject.

ARC Therapy administered by the LIFT System does not meet any of the four above stated criteria and is therefore a nonsignificant risk (NSR) device. Additionally, a non-systematic review of literature on ARC Therapy or similar non-invasive neuromodulation therapies demonstrated negligible device or procedure related SAEs (n=770, Appendix B). For all of the above stated reasons, safety reporting of the primary endpoint in this study will only include observational statistics to describe study/device/procedure related SAEs.

7. Study Design

The Up-LIFT Study is a prospective, single-arm study designed to evaluate the safety and effectiveness of non-invasive electrical spinal stimulation (ARC Therapy) administered by the LIFT System to treat upper extremity functional deficits in people with chronic tetraplegia. The primary endpoint of this pivotal study will report device related safety and changes in established metrics of upper extremity function and strength after treatment with the study device.

Individuals diagnosed with a cervical SCI who are at least 12 months post injury (AIS B-D) that meet all of the inclusion criteria but none of the exclusion criteria will be enrolled. To ensure that the benefits realized in the study are directly attributable to ARC Therapy with LIFT, all enrolled subjects will first undergo a guided, in-clinic conventional functional task practice (FTP) program to regain strength and function of their upper extremity (UE) over a period of approximately two months. Performance gains realized during the wash-in period provide a subject specific control that reflects the limits of

conventional FTP without stimulation (standard of care). At the conclusion of the wash-in period, subjects will complete pre-stimulation baseline testing of UE function.

To test the additive benefit of training with stimulation, combined FTP and ARC Therapy with LIFT will then be administered over a period of approximately two months. FTP will follow established rehabilitation protocols that are specific to the individual subject's needs and capabilities (Gomes-Osman, Tibbett, Poe, & Field-Fote, 2017). Training will be graded to accommodate performance improvement over time, thus maximizing the potential benefit to subjects. To ensure consistency and safety, subjects will participate in a minimum of 12 and a maximum of 20 in-clinic training sessions per month.

All performance metrics will be assessed at enrollment, at the completion of the wash-in period and at the end of the ARC Therapy assessment period. Subjects with clinically meaningful gains in multiple performance domains resulting from the ARC Therapy with LIFT will be considered responders. Additionally, gains during the wash-in (control) period will be compared to gains during the ARC Therapy with LIFT (test) period. Safety will be evaluated throughout the entire study through periodic monitoring and analysis of all reported adverse events. A study flow diagram is illustrated in

Figure 3.

To evaluate the immediate neuroprosthetic effect of active stimulation on strength and motor function, a relevant set of assessments will be performed during a post-treatment follow-up with both stimulation off and on. This optional assessment will be scheduled from 1-30 days after the completion of the 4 month evaluation.

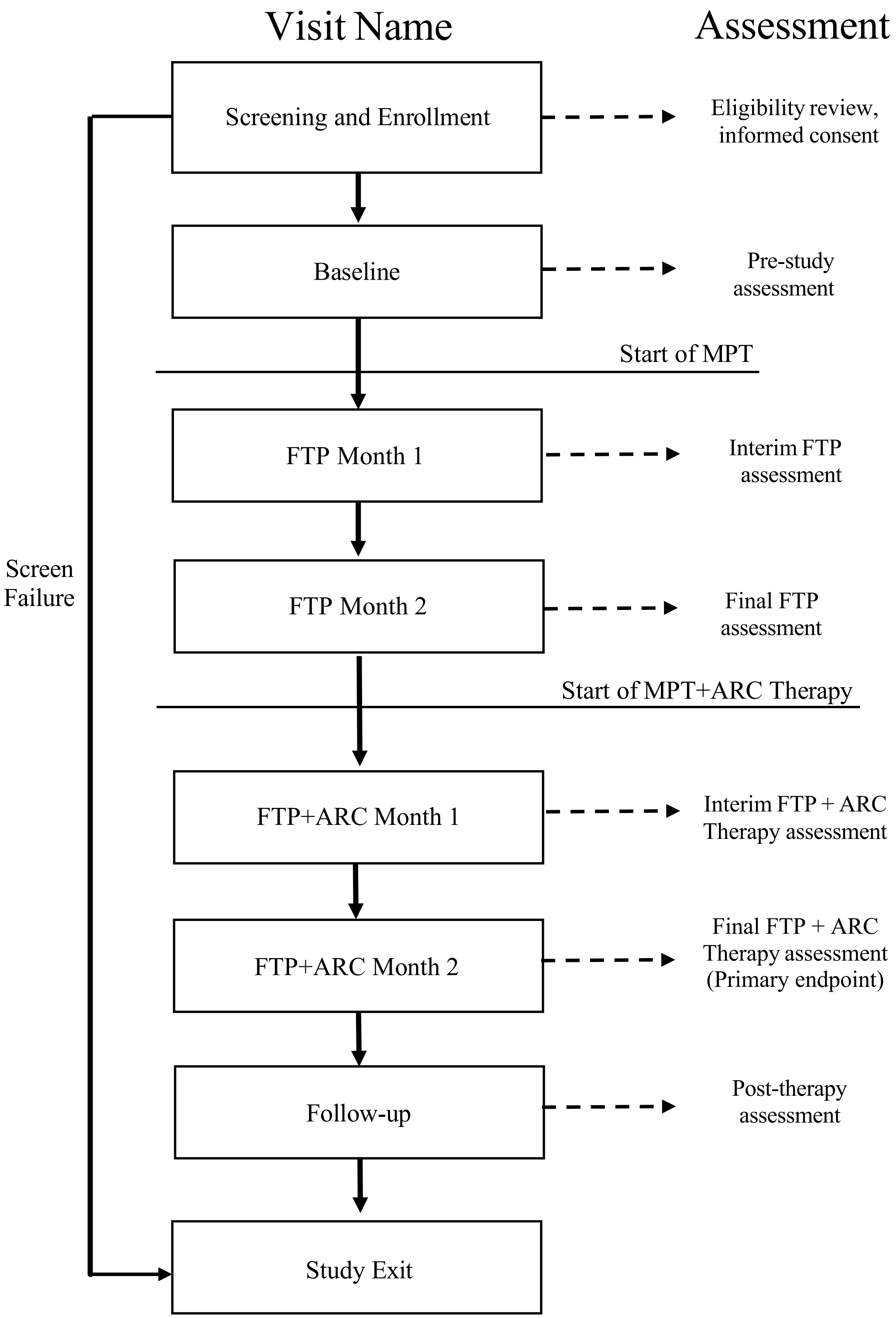

Figure 3. Study flow diagram listing all clinical assessment visits.

7.1. Investigational Sites

Up to 15 sites in the United States (US) and outside the US (OUS) will participate in the study. Investigators with a background in physical medicine and rehabilitation, neurology, occupational or physical therapy focused on spinal cord injury will be selected and will agree to comply with all aspects of the investigational protocol. The Sponsor reserves the right to replace non-participating sites, if needed, to overcome recruitment challenges.

7.2. Study Assessments and Intervals

For the purpose of assessing study endpoints, subjects will be followed for up to approximately 4 months post-enrollment: 2 months of FTP during the wash-in period followed by 2 months of FTP and ARC Therapy with LIFT. Subjects with unexpected logistical difficulty to complete the minimum required number of training sessions in the period allotted will be allowed to extend the training period up to one additional month or until 24 sessions with each therapy mode have been completed, at the discretion of the investigator.

The screening and enrollment visit is used to assess eligibility criteria and to provide informed consent review (

Figure 3). Enrollment maybe done as a separate visit if the subject requires more time to review the patient informed consent (PIC). Pre-study assessments including upper extremity strength and function, co-morbidities and quality of life will be recorded at the baseline visit. During the 2-month wash-in period, based on the baseline assessments, each subject will receive FTP that is tailored to their injury and neurological status. FTP will require subjects to frequent the clinic approximately three times a week for the entire duration of the study period. An interim evaluation of performance will be conducted after 1 month of FTP. At the end of 2 months of FTP (or at the completion of required number of training sessions, if later), assessments will be carried out to capture improvements in strength, functional recovery, and other secondary and observational endpoints. This is followed by the FTP+ARC Therapy phase of the study, where the LIFT System delivers electrical energy during FTP sessions for the next 2 months. An interim and a final evaluation of performance will be performed after months 3 and 4. All baseline assessments will be repeated at these four timepoints. An optional follow-up evaluation will be performed 1-30 days after the 4 month evaluation. It is expected that each subject will maximize the number of FTP and FTP+ARC Therapy sessions during the allotted time period to allow for the greatest potential treatment benefit (up to the allowed 40 total sessions with each treatment).

Assuming approximately 12 months for subject enrollment and a 5-month follow-up period post-primary endpoint, the total study duration is approximately 17 months.

7.3. Subject Withdrawal

All subjects are free to withdraw from participation in this study at any time, for any reason, and without prejudice.

Subjects may be withdrawn from the study for any of the following reasons:

- Subject requests early discontinuation
- Subject is lost to follow-up
- Subject is unable to meet minimum number of desired training sessions

If a subject is withdrawn before completing the study, the reason for withdrawal (assuming the subject is willing and able to share this information) will be entered on the Study Completion Form. Subjects who withdraw before completing the study will be followed only through the date of their withdrawal.

In addition, any comments (spontaneous or elicited) or complaints made by the subject or physician caring for the subject but not involved in the investigation will be documented on the eCRF.

The investigator and the site will make every effort to collect and document information about subjects who are lost to follow-up.

The investigator may terminate a subject from the study at any time for lack of therapeutic effect that is intolerable to the subject, or otherwise considered unacceptable, for intolerable or unacceptable AEs, intercurrent illness, noncompliance with study procedures, administrative reasons, or in the clinical investigator's opinion, to protect the subject's best interest.

8. CRITERIA FOR ELIGIBILITY

8.1. Inclusion Criteria

Subjects must meet all of the following criteria:

1. At least 22 years old and no older than 75 years old at the time of enrollment
2. Non-progressive cervical spinal cord injury from C2-C8 inclusive
3. American Spinal Injury Association (ASIA) Impairment Scale (AIS) classification B, C, or D
4. Indicated for upper extremity training procedures by subject's treating physician, occupational therapist or physical therapist
5. GRASSP-Prehension score ≥ 10 or GRASSP-Strength score ≥30
6. Minimum 12 months post-injury
7. If prescribed anti-spasticity or pain medications, must be at stable dose for at least 4 weeks prior to commencing study procedures
8. Capable of providing informed consent

8.2. Exclusion Criteria

Subjects must not meet any of the following criteria:

1. Has uncontrolled cardiopulmonary disease or cardiac symptoms as determined by the Investigator
2. Has any unstable or significant medical condition that is likely to interfere with study procedures or likely to confound study endpoint evaluations like severe neuropathic pain, depression, mood disorders or other cognitive disorders
3. Has been diagnosed with autonomic dysreflexia that is severe, unstable, and uncontrolled
4. Requires ventilator support
5. Has an autoimmune etiology of spinal cord dysfunction/injury
6. History of additional neurologic disease such as stroke, multiple sclerosis, traumatic brain injury, etc.
7. Peripheral neuropathy (diabetic polyneuropathy, entrapment neuropathy, etc.)
8. Spasms that limit the ability of the subjects to participate in the study training as determined by the Investigator
9. Received Botulinum toxin injections in their upper extremity, neck, or hand within 6 months prior to enrollment
10. Has urinary tract infection or any of the following issues in the upper extremity or the cervical spine region at the time of enrollment: painful musculoskeletal dysfunction unrelated to SCI, unhealed fracture, contracture, pressure sore
11. Breakdown in skin area that will come into contact with electrodes
12. Presence of syringomyelia as confirmed by an MRI
13. Currently undergoing treatment for cancer or has been in remission for less than 2 years
14. Received stem cell treatment within the past two years prior to enrollment
15. Prior nerve or tendon transfer procedure in the upper extremities
16. Total baclofen dose >30 mg per day
17. Has any active implanted medical device
18. Pregnant, planning to become pregnant or currently breastfeeding
19. Concurrent participation in another drug or device trial that may interfere with this study
20. Has undergone a prior course of spinal stimulation therapy directed at UE improvement
21. In the opinion of the investigators, the study is not safe or appropriate for the participant and/or the subject is unlikely to return for the follow-up visits per the protocol

9. SCHEDULE OF EVENTS

Assessment	Pre-Treatment Visits[1]			FTP[2]				FTP + ARC Therapy[2]				Follow-up[7]
	Screening	Enrollment	Baseline	Therapy Visits	1 mo[3] (±7 d)	Therapy Visits	2 mo[3] (±7 d)	Therapy Visits	3 mo[3] (±7 d)	Therapy Visits	4 mo[3] (±7 d)	4 mo (+30 d)
Informed Consent	X			12-20 sessions/month[5]		12-20 sessions/month[5]		12-20 sessions/month[5]		12-20 sessions/month[5]		
Inclusion/Exclusion Criteria	X											
Medical History		X										
Surgical History		X										
Medications		X			X		X		X		X	X
Occupational Therapy History		X										
Urine Pregnancy Test		X					X					
MRI	X[6]											
ISNCSCI			X		X		X		X		X	
GRASSP	X				X		X		X		X	X
Pinch & Grasp Force			X		X		X		X		X	X
CUE-T			X		X		X		X		X	X
NRS for Pain			X		X		X		X		X	
ISCI Pain Data Set			X		X		X		X		X	
SCIM III			X		X		X		X		X	
MOS-S			X		X		X		X		X	
PSFS			X		X		X		X		X	
EQ-5D-5L			X		X		X		X		X	
WHOQOL-BREF			X		X		X		X		X	
ISAFSCI			X		X		X		X		X	
PHQ-9			X		X		X		X		X	
PGIC					X		X		X		X	X
CGIC					X		X		X		X	X
Adverse Events	X	X	X		X		X		X		X	X
Study Exit	X[4]	X[4]	X[4]		X[4]		X[4]		X[4]		X[4]	X

Table 7. Schedule of events for the study.

1. *Screening and enrollment or enrollment and baseline can be done as single visits.*
2. *To ensure that study subjects undergo at least 24 in-clinic FTP and 24 FTP+ARC Therapy training sessions, final assessment may be extended by an additional month.*
3. *Interim and final assessments should be scheduled after each monthly training period is completed.*
4. *Complete the Study Exit CRF at completion of the study or at study termination if occurring earlier.*
5. *Optimal electrode placement and stimulation amplitude may be determined using motor evoked potential [MEP] testing. This is an optional assessment.*
6. *Optional assessment to confirm the presence of syringomyelia if suggested by symptoms.*
7. *Optional 2-day assessment performed with ARC Therapy OFF (day 1) then ON (day 2) from 1-30 days after completion of 4-month evaluation. Not needed to repeat testing with stim OFF if performed within 3 days of 4 month visit.*

10. STUDY PROCEDURES

10.1. Screening and Enrollment

When a suitable candidate indicated for ARC Therapy presents to the clinic for consideration for enrollment in the study, the investigator will explain the potential risks and benefits of participation to the patient. Patients will be provided with a copy of the informed consent for review and will be given ample opportunity to read and pose questions they may have about the study. During the enrollment visit, patient’s medical, surgical, and occupational therapy histories will be recorded along with their prescribed list of medications. Additionally, GRASSP instrument will be administered to assess eligibility. Since study subjects have chronic SCI, the recorded values will be treated as the patient's baseline GRASSP score. If a patient subsequently fails to meet eligibility criteria, they will be considered a screening failure and may receive standard of care for their condition outside of the study. If the patient is deemed eligible to participate in the study, they will be sent home with the PIC to discuss the study with their caregiver(s). If after review, the patient agrees to participate, the informed consent will be signed by the patient and recorded on a screening/enrollment log at this visit. In the event the patient is unable to sign the PIC, a legal representative may sign the informed consent on her/his behalf.

10.2. Baseline Evaluations

Baseline evaluations will be performed as listed in Table 7. Schedule of events for the study.

Subjects will perform the complete suite of required strength and functional tests. Questionnaires related to strength and function associated with upper extremity function along with those associated with autonomic function, quality of life and additional diseases symptoms will be captured. Any ongoing medical condition not associated with the condition being treated will also be documented.

10.3. Functional Task Practice (Wash-in Period)

Once enrolled, subjects will be assigned an FTP regimen that takes into account their injury, medical history, and existing body of knowledge on UE neurorestorative training. The PI and research team will establish the benefits of an FTP program while identifying any barriers to implementation before coming up with programs tailored for individual study subjects.

Subjects will undergo FTP and ARC Therapy with LIFT for 2 months participating in an average of 3 sessions/week for a total of 12-20 sessions/month. To ensure homogeneity across sites, FTP is defined as 1-2 hours of training covering at least 5 categories of task specific training including pinch, grasp, grasp with rotation, and whole arm movement. The exact FTP regimen for the subject will be determined by the study investigator in consultation with other research personnel at the site. The exercise training program will be comprised of functional tasks that included repetitive activities of gross upper extremity movement, isolated finger movements, plus simple

and complex pinch, and grasp performance measures. For each category, 4-10 occupational therapy activities with various difficulty levels will be identified. Each subject will perform at least 1-2 exercises within the same category during each treatment session. Activities in each category will be chosen according to the subjects' ability and modified as the ability to perform the functional task progresses over time (graded training). Typical movement patterns will be encouraged by guidance and feedback. When a subject has little to no voluntary movement, active assistance from a physical or occupational therapist, or qualified clinical team member will be provided to complete the desired activity. At the end of each session, subjects will be assessed for improvements in upper extremity function and strength gained from the exercise training through a simple box and block test.

Vitals should be collected at a minimum of 3 times per session.

- At the start of the session prior to electrode placement
- At mid-point of the session when peak values may result
- At the end of the session, before the daily assessments

10.3.1. Interim FTP Assessment

This assessment is scheduled after 1 month of FTP to capture any interim improvements from the training. At this visit, assessments including upper extremity strength and function, pain, spasticity, sleep, autonomic functions, quality of life and global impression of change will be captured. Adverse events, if any, and change in medications will also be recorded.

10.3.2 Final FTP Assessment

This assessment is scheduled after 2 months of FTP. At this visit, all assessments performed at baseline will be repeated including upper extremity strength and function, pain, spasticity, sleep, autonomic functions, quality of life and global impression of change. Adverse events, if any, and change in medications will also be recorded. Outcomes collected at this visit will serve as a pre-stimulation baseline. To ensure that study subjects undergo at least 24 in-clinic FTP training sessions, final assessment may be extended by an additional month.

10.4. Functional Task Practice with ARC Therapy (Stimulation Period)

After their FTP alone training is completed, subjects will undergo FTP and ARC Therapy administered by the LIFT System for an additional 2 months with between 12-20 sessions/month. ARC will be administered using the LIFT System as described in Section 4.2.1. in conjunction with a similar FTP regimen as before.

The LIFT System and ARC Therapy Delivery: The LIFT System delivers programmable electrical stimulation that is comprised of two modulated frequencies: a base frequency (usually 15-30 Hz) and an overlapping frequency that is usually 10 kHz, on up to four independent channels (Figure 4). In this illustration, biphasic rectangular waveform 1 ms in duration and a 10 kHz overlapping frequency that repeats 30 cycles/second (30 Hz) is applied over the cervical spine. The overlapping

frequency is believed to either block unmyelinated, small C-fibers associated with skin nociceptors or bypass the capacitive reactance to lower the overall skin impedance. This allows for safe, tolerable deeper penetration of the resulting electric field thereby reaching the deeper dorsal roots in the epidural space.

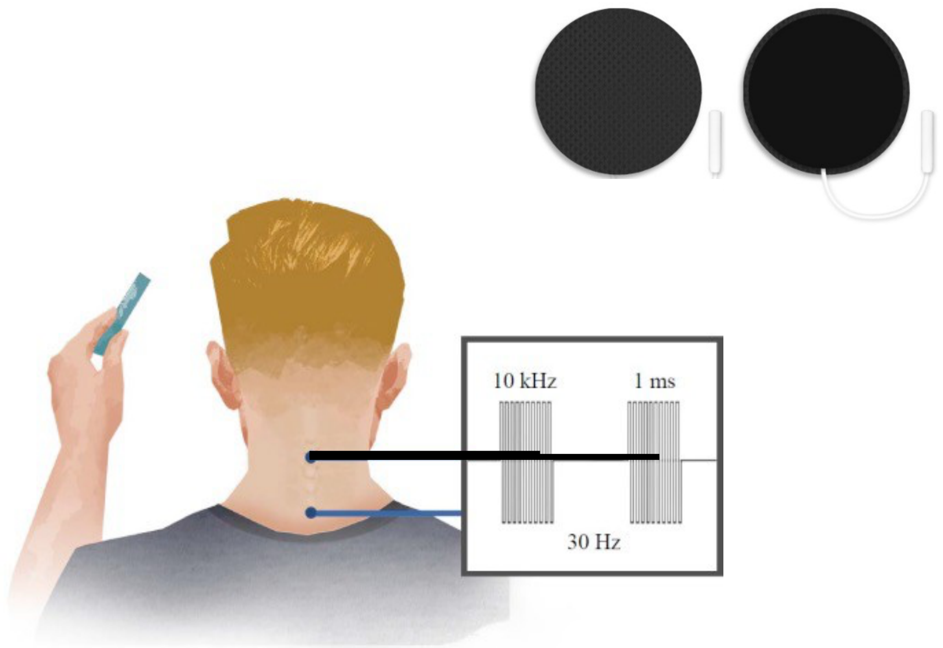

Figure 4. Potential electrode locations for stimulation and a sample stimulation waveform (bottom inset) using round hydrogel electrodes (top inset). Figure adapted from Inanici et al. (Inanici, et al., 2018).

Stimulation may be delivered via commercially available 1.25” round electrodes as cathodes and two 1.5” x 3.5” rectangular plates as anodes (Axelgaard Manufacturing Co. Ltd., USA) or equivalent. Cathode electrodes are usually placed midline on the skin of the neck, one above and one below the injury site, using inion and spinous processes as landmarks. Additional electrodes may be placed per investigators’ discretion. Anode electrodes may be placed symmetrically parallel to the iliac crests or other locations deemed safe and effective. Electrodes of any size can serve as cathodes and anodes so long as they do not violate the stimulator specification for maximum charge density which is intended to reduce the risk of skin damage/burns. It is suggested that stimulation be applied for between 1-2 hours during the session in conjunction with FTP.

The stimulation protocol in this study was adapted from Inanici et al. (Inanici, et al., 2018). Subjects will undergo neurophysiologic testing with the LIFT System, to assess if their neurological state will respond to stimulation therapy and what their threshold potential may be. In the absence of MEP recording capabilities, optimal stimulation parameters for each subject will be based on the motor responses that were tested in the first stimulation session. Subthreshold stimulation intensity is used. Stimulation intensity may be adjusted to maximize task performance, typically using the least amount of energy required to achieve the desired effect. Monophasic or biphasic stimulation waveforms, whichever elicits a better response, may be utilized. It is

suggested that the stimulation intensity be increased gradually (e.g., 5 mA steps) until muscle tone begins to interfere with coordination or subjects' comfort level. The specific stimulation parameters and optimal stimulation amplitude is left to the discretion of the investigator and the research team. The duration and frequency of therapy sessions will be graded to maximize each subject's performance gains. Heart rate and blood pressure will be monitored throughout each therapy session.

Details on application and use of the LIFT System for upper extremity motor recovery including recommended stimulation location and settings are described in the LIFT Instruction Manual.

10.4.1. Interim FTP+ARC Therapy Assessment

This assessment is scheduled after 1 month of FTP+ARC Therapy to capture any interim improvements from the stimulation plus training. At this visit, all assessments performed at baseline will be repeated including upper extremity strength and function, pain, spasticity, sleep, autonomic functions, quality of life and global impression of change. Adverse events, if any, and change in medications will also be recorded.

10.4.2. Final FTP+ARC Therapy Assessment – Primary Endpoint Visit

This visit is scheduled after 2 months of FTP and ARC Therapy with LIFT and serves as the primary endpoint assessment visit. At this visit, all assessments performed at baseline will be repeated including upper extremity strength and function, pain, spasticity, sleep, autonomic functions, quality of life and global impression of change. To ensure that study subjects undergo at least 24 in-clinic FTP+ARC Therapy training sessions, final assessment may be extended by an additional month. Adverse events, if any, and change in medications will also be recorded. Subjects will complete the Study Exit form concluding their participation in the study. Any ongoing AEs will be followed-up until resolution or until the subject reaches the end of the study.

The assessments performed and questionnaires completed at the 5 major study visits (Baseline, Interim FTP, Final FTP, Interim FTP+ARC Therapy, Final FTP+ARC Therapy) typically take several hours to complete. To ease the burden on the subject, the investigator may choose to administer them on 2 consecutive visits.

10.5. Follow-up Assessment (optional)

Following the completion of the 4 month visit, subjects will perform a relevant set of assessments during a two day follow-up visit to occur from 1-30 days later. These assessments will include GRASSP Strength, GRASSP Prehension, CUE-T, pinch force, grasp force and the Global Impression of Change questionnaire. Each assessment will be performed twice: on day one without stimulation and on day two with active ARC Therapy stimulation. If the follow-up assessment is performed within 3 days of the 4 month visit, then it is not necessary to repeat the assessments with stimulation off. Stimulator settings should generally duplicate those used most recently during training but may be optimized as needed.

10.6. Medications

As the two drug classes most likely to influence the study outcomes, it is required that spasticity and pain medications are maintained at a stable dose for at least four weeks prior to the commencement of study procedures. Patients should consult with their primary care physician (PCP) and their investigator if they have any questions or concerns. The study entry criteria stipulate that baclofen intake in potential subjects should not exceed 30 mg per day. Patients on higher doses of baclofen shall consult with their PCP to determine if they can be titrated safely to a lower dose that meets the study inclusion criterion. This new dosage should be maintained stable for 4 weeks before the subject can participate in any study procedure. It is also recommended that subjects take their baclofen at least eight hours prior to their next FTP or FTP+ARC Therapy session.

10.7. Medical History

Individuals with a history of traumatic brain injury may be qualified for enrollment if, in the opinion of the investigator, there is no cognitive impairment that would limit the ability to provide informed consent or limit the ability to follow directions or perform study activities. Individuals with a history of peripheral neuropathy may be qualified for enrollment if, in the opinion of the investigator, these conditions are mild, have been resolved or are stable such that they will not impact the individual's ability to comply with the study requirements or result in an increased risk to the subject participating in the study.

10.8. COVID-19 Accommodations

Sites invited to participate in the study will undergo a site qualification process to ensure that they meet the minimum requirements for inclusion in the study. It is expected that local guidelines governing safety and risk management practices for subjects will result in site-specific differences such as treatment venues and caregiver-subject interactions. In the event that local guidelines change during the study, e.g., to accommodate increased infection risk to subjects, the Sponsor and investigator will review the study requirements for participating sites to ensure that study procedures may continue without unwarranted risk to subjects and investigators and without impacting the results of the study. It is understood that these accommodations may include the increased use of remote monitoring of subjects, delivery of assessments in socially distanced settings and other similar adaptations to the local standard of care.

10.9. Early Withdrawal or Early Termination Evaluation

All subjects are free to withdraw from participation in this study at any time, for any reason, and without prejudice. Subjects may be withdrawn from the study when they request early discontinuation or are lost to follow-up.

The clinical investigator may terminate a subject from the study at any time for lack of therapeutic effect, or otherwise considered unacceptable, for intolerable or

unacceptable AEs, intercurrent illness, noncompliance with study procedures, administrative reasons, or in the clinical investigator's opinion, to protect the subject's best interest.

11. STUDY OUTCOMES

11.1. International Standard Neurological Classification of Spinal Cord Injury (ISNCSCI)

The International Standards for Neurological Classification of Spinal Cord Injury (ISNCSCI) or more commonly referred to as the ASIA Impairment Scale (AIS), was developed by the American Spinal Injury Association (ASIA) as a universal classification tool for Spinal Cord Injury based on a standardized sensory and motor assessment, with the most recent revised edition published in 2019. The impairment scale involves both a motor and sensory examination to determine the sensory and motor levels for the right and left side, the overall neurological level of the injury and completeness of the injury.

ISNCSCI motor and sensory scores are derived from 4 sub-scores: upper and lower extremities and left and right sides. Upper extremity and lower extremity motor scores (UEMS, LEMS) are derived from grading 5 muscles each in the upper and lower extremities on a scale of 0 (total paralysis) to 5 (normal active movement, full range of motion against gravity and sufficient resistance). Sensory scores are derived similarly for upper and lower extremities by grading pin prick and light touch sensation on a scale of 0 (absent) to 2 (normal or intact).

Domains: Motor, sensory, neurological levels of sensory and motor, neurological level of injury, complete or incomplete, ASIA Impairment Scale (AIS) and Zone of Partial Preservation (ZPP)

Scoring and range: Motor score: 0-100, sensory score (light touch and pinprick: 0-112 each)

Recall period: Instantaneous

Validation literature: (Graves, Frankiewicz, & Donovan, 2006)

11.2. Graded Refined Assessment of Strength, Sensibility and Prehension (GRASSP)

The GRASSP is a clinical impairment measure specific to the upper limb for use after tetraplegia. It is a multimodal test that measures sensorimotor and prehension function in three domains important in describing arm and hand function (strength, sensibility and prehension) comprising five subtests for each upper limb: strength, dorsal sensation, palmar sensation, prehension ability and prehension performance. These numerical scores provide a comprehensive profile of upper-limb function.

GRASSP Strength (strength) - assessed by testing for muscle contraction and range of motion with or without gravity and graded as 0 (no palpable muscle contraction) to 5 (full range of motion against gravity with maximum resistance).

GRASSP Sensibility (dorsal sensation and palmar sensation) - tested using Semmes-Weinstein 4 monofilament probes. The pressure applied and sensation elicited was represented by numeric values ranging from 0 (no response) to 4 (normal sensation).

GRASSP Prehension (prehension ability and performance) - divided into ability vs. performance. This domain represents the influence of sensation and strength on goal-oriented upper limb tasks like cylindrical grasp and lateral pinch along with 6 timed tasks.

The GRASSP was developed with the intent to be a clinical and a research tool that would:

- capture information on upper limb impairment from the traumatic tetraplegic population
- obtain integrated sensory and motor impairment data, and discriminate the population according to impairment and function
- be responsive (sensitive) to change over time
- assess the extent of spontaneous (natural) recovery
- be applied in clinical settings and in clinical trials/studies to evaluate the effect of novel interventions

Domains: Strength, sensation, prehension

Scoring: sensation (dorsal and palmar, 0-12 each), strength (Ten muscles per upper limb graded from 0-5 for a total 50 for each upper limb), prehension: ability (12) and performance (30)

Recall period: Instantaneous

Validation literature: (Kalsi-Ryan, Beaton, Curt, Duff, & Popovic, 2012)

11.3. Pinch and Grasp Force

Pinch and grasp force will be measured by the Commander Echo Console with the Pinch and Hand Dynamometer (JTech Medical, Salt Lake City, UT) to quantify finger and grasp strength.

Domains: Force

Scoring: Up to 222N

Recall period: Instantaneous

11.4. Capabilities of Upper Extremity Test (CUE-T)

CUE-T is an assessment tool that measures functional limitation and assesses the amount of difficulty experienced in performing specific actions with one or both arms and hands in individuals with tetraplegia. Questions focus on the individuals' ability to reach or lift; pull and push with their arms; move and position their arm and wrist; use their hand and fingers; and press with the tip of the index finger.

Domains: Activity – subcategory: mobility

Scoring and range: 1 – Totally limited, can't do it at all to 7 – Not at all limited; 32 – 224

Recall period: Instantaneous

Validation literature: (Marino, Kern, Leiby, Mary Schmidt-Read, & Mulcahey, 2015)

11.5. Numerical Rating Scale for Pain

An anchored 0-10 rating for pain where "0" represents no pain and 10 represents worst pain imaginable. The recall period can be adjusted based on how the question is phrased (e.g., in the last 7 days, rate your average pain on a scale of 0-10). The score usually represents the average pain experienced by the subject in the recall period.

Recall period: Variable based on how the question is posed.

11.6. International Spinal Cord Injury Pain Data Set (ISCIPDS)

To standardize collection and reporting of pain in SCI, ISCIPDS was developed by an international consortium of pain and SCI experts. It collects pain interference with day-to-day activities, mood, and sleep over a 7-day recall period.

Domains: 2 (Interference and worst pain problem(s))

Scoring: Interference: 0 (no interference) – 10 (extreme interference)

Recall period: 7-days

Validation literature: (Widerström-Noga, et al., 2008)

11.7. Medical Outcomes Study (MOS) Sleep Score

This is a sleep scale developed for the Medical Outcomes Study (MOS), a two-year study of subjects with chronic conditions. MOS-Sleep contains 10 self-rated questions on sleep duration, sleep disturbance, adequacy, and somnolence.

Domains: Not applicable

Scoring and range: 1 (all of the time) – 6 (none of the time); 1 – 60 points

Recall period: 4 weeks

Validation Literature: (Hays R. &., 1992)

11.8. Spinal Cord Independence Measure (SCIM III)

The SCIM has been developed to address three specific areas of function in subjects with spinal cord injuries (SCI).

- self-care (feeding, grooming, bathing, and dressing)
- respiration and sphincter management
- mobility (bed and transfers and indoors/outdoors)

Additionally, the SCIM can also be used to help guide clinicians in determining treatment goals and objectives for subjects with a SCI. By helping clinicians determine

areas of limitations for their subjects with spinal cord injuries, both therapists and subjects alike will be benefitting from this functional measurement tool.

Domains: Self-care, respiration and sphincter management, mobility

Scoring: self-care:20, respiration, and sphincter management: 40, mobility: 40

Range: 0 – 100

Anchor: 0 – total dependence, 100 – complete independence

Validation literature: (Catz, Itzkovich, Agranov, Ring, & Tamir, 1997)

11.9. Penn Spasm Frequency Scale (PSFS)

A two-component, self-reported measure of frequency and severity of spasms after spinal cord injury.

Domains: Frequency, severity

Scoring: Frequency (0-4), severity (1-3)

Anchor: Frequency (0-No spasms to 4-Spontaneous spasms occurring more than ten times per hour), severity (1-Mild to 3-Severe)

Validation literature: (Adams, Ginis, & Hicks, 2007)

11.10. 5-Dimension, 5-Level European Quality of Life (EQ-5D-5L)

EQ-5D instrument comprises a short descriptive system questionnaire to assess the health state of an individual. Each state is represented by a 5-digit number ranging from 0 through 4 (e.g., 42311), where each number represents an individual domain. The number is then translated into an index score. The questionnaire also has a visual analogue scale (EQ VAS) to capture the health state.

Domains: 5 (Mobility, self-care, usual activities, pain/discomfort, anxiety/depression)

Scoring and range: VAS: 0 (Worst health imaginable) –100 (best health imaginable); EQ-5D-5L Index: 0.000 – 1.000

Recall period: Same day

Validation literature: (Herdman, et al., 2011)

11.11. World Health Organization Quality of Life Measure (WHOQOL-BREF)

WHOQOL-BREF contains 26 questions in 4 domains with 24 of them assigned to the facets/areas relevant to quality of life. Two questions address overall quality of life and general health.

Domains: Physical health, psychological, social relationships, environment

Scoring and range: Transformed scores (4-20 or 0-100)

Validation literature: (Jang, Hsieh, Wang, & Wu, 2004)

11.12. International Standards to document remaining Autonomic Function after Spinal Cord Injury (ISAFSCI)

A semi-quantitative questionnaire on autonomic control including heart function, blood pressure, sweating, temperature regulation, broncho-pulmonary system, bladder and bowel management and sexual function.

Scoring: Normal/abnormal (heart function, blood pressure, sweating, temperature regulation, broncho-pulmonary system); 0 – Complete loss of control through 2 – Normal function (bladder and bowel management, sexual function)

Relevant literature: (Krassioukov, et al., 2012)

11.13. 9-Item Patient Health Questionnaire

A nine-item, self-reported measure of depression.

Domains: N/A

Scoring and range: 0 (Not at all) – 3 (Nearly every day); 0-27

Recall period week: 2 weeks

Validation literature: (Kroenke, Spitzer, & Williams, 2001)

11.14. Patient/Clinician Global Impression of Change (PGIC/CGIC)

Global impression of change (GIC) is a 9-point Likert scale used to assess treatment-induced changes in a subject's clinical status (improvement or decline). The GIC will provide a general indication of changes related to activity limitations, symptoms, emotions, and overall quality of life. The questionnaire is completed both by the study subjects (Patient GIC) and the physicians (Clinician GIC) and may be used to validate the relative clinical benefit of treatment as quantified by other outcome measures used in the study.

Range: NA

Anchor: Very much improved, much improved, moderately improved, minimally improved, no change, minimally worse, moderately worse, much worse, very much worse

11.15. Motor Evoked Potential (MEP)

This is an optional assessment that may be used to optimize settings for ARC Therapy for sites that have the appropriate training and equipment to capture MEPs.

A motor evoked potential (MEP) is an electrical potential recorded from a specific muscle or group of muscles that results from applying an electrical stimulus to the spinal cord. Using the LIFT device, electrical potentials at 1 Hz, 0.5-1 ms, and 30 Hz (monophasic rectified waveform) will be applied to a spinal segment, first below then above the level of injury, and possibly in combination. The output current is slowly increased until noticeable changes are observed on the surface EMG recording electrodes placed over select muscles and/or groups of muscles. Visible muscle twitching, contracture, and joint movement will also be noted.

MEP during baseline may be used to assess the threshold response to ARC Therapy for that particular subject. Comparing baseline recordings to future recordings captured during periodic assessment visits will also document changes in response to treatment over time.

12. SUBJECT BENEFITS AND RISKS

12.1. Potential Benefits to Study Subjects

Receiving benefit from participation in the study is not guaranteed. Anticipated benefits to subjects may include, but are not limited to, the following:

- Improved hand and/or arm muscle strength and prehension (ability to pinch, grasp)
- Improved light touch and/or pinprick sensation in the dermatomes at, below or above the level of lesion
- Improved quality of life
- Improved bladder, bowel, or sexual function
- Reduced frequency of spasticity (if present at baseline)

12.2. Potential Risks to Study Subjects

Risks to study subjects enrolled in this study include all those risks commonly associated with all TENS and NMES devices including skin rash at the site of application of the surface electrode, electric shock from the stimulator unit, unpleasant tingling or buzzing sensation at skin surface and cramping of the muscles. Other risks are identified in Sections 13.4 and 13.5.

12.3. Data Safety Monitoring Board

An independent data safety monitoring board (DSMB) consisting of members not otherwise associated with the trial will meet periodically throughout the study to review study safety and advise the Sponsor on safe conduct. The DSMB will review reported SAEs and may be asked to confirm investigator-reported causality relative to the study device and procedures. In the event of an unexpectedly high incidence of safety events, the DSMB will advise the Sponsor on the continuation of the study. The mode of operation of the DSMB will be defined in a DSMB Charter. A description of the DSMB mode of operation is provided in Appendix A.

12.4. Clinical Risk Analysis

12.4.1. Increased Risks to Subjects Posed by the Investigation

All subjects who are enrolled in the study will be carefully screened to ensure that they meet enrollment criteria which is intended to minimize risk. Subjects will undergo pre-procedure testing mandated by the protocol which is considered standard of care at participating institutions. Additionally, during the course of the study, the occupational therapy administered during the first phase of the study (frequency and total number of training sessions), stimulation regimen that follows, and most of the assessments performed at study visits,

constitute standard of care at these institutions. Therefore, there are no added risks posed to study subjects by the investigation.

12.4.2. Manner in Which Risks Will be Minimized

The investigators and the research coordinators are trained per protocol to perform the assessments and stimulation consistent with standard clinical practice. A risk management plan (Minnetronix Document #DP-0004-282-102, Rev A) of the LIFT System was developed per ISO 14971 Application of Risk Management to Medical Devices by Minnetronix Medical Inc. (St. Paul, MN). A risk-benefit analysis identified hazards and estimated risks associated with the manufacture (including factors relating to device design, choice of materials, software) and the use of the device (ISO 14971) (Table 8). lists the description of actions that have been taken to mitigate the identified risks. Precautions have been taken to minimize or eliminate risks through appropriate design controls which appear to have been confirmed through extensive clinical use in a feasibility study setting. Refer to Section 3.6.

Identified Risk	Reasons for Risk
Adverse reactions to skin-contacting materials	Inappropriate product materials used for skin-contacting interfaces<br>Allergic response to skin-contacting materials
Electrical, Mechanical, or Thermal Hazards that may result in user discomfort or injury	Inappropriate system design and technical parameters<br>Insufficient Electrical, Mechanical, and Thermal Safety Testing<br>Insufficient Electromagnetic Compatibility Testing<br>Insufficient Electrode Performance Testing<br>Insufficient Software Verification, Validation, and Hazard Analysis<br>Insufficient Device Labeling<br>Insufficient Instructions for Use
Misuse that may result in user discomfort or injury	Insufficient Device Labeling<br>Insufficient Instructions for Use
Unintentional harm due to secondary systemic effects	Insufficient Clinical Performance Data<br>Insufficient Device Labeling<br>Insufficient Instructions for Use

Table 8. A summary of anticipated risks related to the use of the device.

Identified Risk	Mitigation Measures
Adverse reactions to skin-contacting materials	System design<br>Biocompatibility Testing per ISO 10993-10<br>Product Labeling
Electrical, Mechanical, or Thermal Hazards that may result in user discomfort or injury	System Design, including:<br>- Compliance w/ all applicable safety standards, e.g., IEC 60601-1, IEC 60601-2-10, etc.<br>- Option for user to stop stimulation at any time during treatment<br>- Only clinical staff can program stimulator unit<br>- Clinical staff required to program stimulator with stimulation parameters specific to each patient
	- Option for user to increase or decrease the stimulation current output within a defined range<br>- Default safety shut down if system impedance is out of acceptable range<br>Electromagnetic Compatibility Testing per IEC 60601-1-2<br>Electrical, Mechanical, and Thermal Safety Testing per applicable safety standards<br>Electrode Performance Testing<br>Software Verification, Validation, and Hazard Analysis per IEC 62304<br>Usability Engineering per IEC 60601-1-6 and IEC 62366<br>Product Labeling<br>Instructions for Use
Misuse that may result in user discomfort or injury	System design<br>Product Labeling<br>Instructions for Use
Unintentional harm due to secondary systemic effects	Clinical Performance Data<br>Product Labeling<br>Instructions for Use

Table 9. A summary of the proposed mitigation(s) for each risk identified in Table 8.

Subjects treated with the study device will undergo established stimulation protocols whose location of application, dose, frequency, and outcomes have been documented in the literature (Gad, et al., 2018) (Inanici, et al., 2018). Investigators will be required to undergo extensive training on the Instructions for Use and best practices when using the LIFT System. The procedural and follow-up requirements and recommendations within this clinical investigational plan were developed in accordance with these references and input from investigators with the intention that these subjects will be managed consistently. Moreover, care was taken to exclude any subject that may present with an added risk in response to the study procedures.

In addition, participating sites have been chosen for their demonstrated proficiency using either the LIFT System or a similar external electrical stimulator. Additionally, investigators and research coordinators will be trained extensively on the requirements of the protocol including use of the LIFT System. Field clinical specialists trained in the use of the study device and the requirements of the protocol may monitor stimulation sessions in the study. Sites will be monitored soon after enrollment begins to identify and address protocol deviations and unsafe practices, if any exist, in a timely manner.

12.4.3. Justification for the Investigation

The body of clinical evidence to support the potential benefit of ARC Therapy for people with SCI is growing. However, these studies have been limited to a handful of prospective and retrospective case series and/or reports. These studies used different stimulation regimen, outcome measures and heterogenous inclusion/exclusion criteria resulting in modest to good improvements that are clinically relevant. Perhaps most significantly, none of the studies defined an a priori success criterion that was based on MCID, MDD or MID in the chronic SCI population. Thus, the strength of the evidence supporting the utility of ARC Therapy to restore arm/hand function is moderate.

By conducting a large scale, prospective, multicenter pivotal study, the Sponsor aims to improve the quality of evidence in support of ARC Therapy administered by the LIFT System for the restoration of hand/arm function in subjects with cervical SCI.

The absence of well-defined MCIDs or MDDs for the chronic SCI population has inhibited the ability to objectively assess treatment outcomes. For example, the pinch and grasp forces reported in previous studies do not have well-defined clinically meaningful improvements. Lack of prospective studies, small sample size, lack of a well-defined controlled group and lack of feedback from the trial participants as to what they perceive as clinically meaningful change were a few factors that has hampered progress in defining what constitutes a meaningful change. This study addresses several of the aforementioned limitations. A pragmatic approach was used to define a responder to primary study measures using MID. Additionally, predefined sensitivity analyses will help define a validated MID for this subject population. Finally, by incorporating GIC and tracking the progress of the subjects every 4 weeks, it is likely that this study may ultimately be able to objectively define MCID and MDD for some of the outcome measures.

12.4.4. Description of Patient Population

A pre-defined sample of individuals with chronic cervical SCI will be enrolled such that a statistically valid result may be obtained. In general, the distribution of age and gender is expected to reflect the incidence of SCI in the US.

Per protocol, all subjects will have sustained a SCI injury over 12 months ago and will be on stable spasticity and pain medications and exercise to treat their

symptoms. Thus, the patient population being studied are ideal candidates for further treatment with ARC Therapy to improve hand/arm function.

12.4.5. Adverse Events

The definitions for adverse events, adverse device effects and unanticipated adverse device effects are provided in Section 13.1 of the protocol. A tabulation of procedure and device related expected AEs are listed in Section 13.5. Guidance for rating the AEs on severity and relationship is provided in Sections 13.11and 13.12, respectively.

12.5. Conclusion

Prior investigations using the LIFT System have demonstrated safety with a relatively low incidence of AEs. The majority of these AEs were unrelated to the stimulation or the procedure. Those that were stimulation or procedure related were transient and resolved when surface electrodes were removed, and stimulation was turned off. Additionally, the site research staff will be trained on the use of the device and made aware of possible AEs during the study. In summary, the established safety profile and minimal risk posed by the stimulation session warrants the study of the LIFT System to benefit the SCI community.

13. SAFETY DEFINITIONS AND REPORTING REQUIREMENTS

13.1. Adverse Events

An AE is defined as any untoward medical occurrence in a study subject. This definition does not imply that there is a relationship between the adverse event and the device under investigation.

Investigators in this study are required to report all AEs beginning at the time of enrollment and continuing until the subject exits the study. Subjects who fail screening (i.e., those who have signed consent but subsequently fail to meet enrollment criteria) will be followed for 72 hours or until discharge, whichever occurs first.

Investigators must obtain all information available to determine the causality and severity of the AE and to assess whether it meets the criteria for classification as an SAE requiring immediate notification to the Sponsor or its designated representative. All reported AEs will be documented on the appropriate eCRF and will include the event description (sign, symptom, or diagnosis), onset, causality, resolution, seriousness, severity, and action taken.

All AEs will be followed by the Investigator until resolution or until the end of study participation.

An AE will not be reported if it existed at the time of enrollment and continued unchanged thereafter unless the event worsened considerably and required additional medical treatment. Investigators in this study are required to report all AEs except for the following:

- Standard follow-up or exacerbation of pre-existing conditions unrelated to the treatment including diabetes, cancer/tumors, allergies, osteoporosis, arthritis etc.

- Physical trauma determined to be unrelated to the FTP or ARC Therapy including pain due to musculoskeletal injuries, muscle aches due to over-exertion, joint degeneration, tendonitis, and bursitis.
- Newly developed disease or illness unrelated to FTP or ARC Therapy.
- Common ailments unrelated to SCI, the FTP or ARC Therapy or the drugs or interventions used to treat SCI including common headache, muscle pain, nausea, constipation, upper respiratory infections, or influenza.

13.2. Serious Adverse Events

An adverse event is defined as serious if the adverse event:

a) led to death, injury or permanent impairment to a body structure or a body function
b) led to serious deterioration in the health of the subject, that either resulted in
    1) a life-threatening illness or injury, or
    2) a permanent impairment of a body structure or a body function, or
    3) in-patient or prolonged hospitalization, or
    4) medical or surgical intervention to prevent life-threatening illness,
c) led to fetal distress, fetal death or a congenital abnormality or birth defect

13.3. Hospitalizations

Hospitalization is defined as an overnight stay in a hospital unit and/or emergency room. Planned hospitalization for a pre-existing condition, or a procedure specified by the Clinical Investigational Plan, without serious deterioration in health, is not considered an SAE. Treatment on an emergency or outpatient basis for an event not fulfilling the definition of seriousness given above and not resulting in hospitalization should be reported as an AE only.

The following reasons for hospitalizations are not considered AEs, and therefore not reportable as SAEs:

- Hospitalizations for cosmetic elective surgery, social and/or convenience reasons.
- Standard monitoring of a pre-existing disease or medical condition that did not worsen, e.g., hospitalization for coronary angiography in a subject with stable angina pectoris.
- Elective treatment of a pre-existing disease or medical condition that did not worsen, e.g., hospitalization for chemotherapy for cancer.

13.4. Primary Serious Adverse Events

Serious adverse events may contribute to the primary safety endpoint if related to the use of ARC Therapy administered by the LIFT System or the study procedures. SAEs that meet the severity criteria described in the table below will contribute to the primary safety endpoint in subjects who have been exposed to the study device (Table 10. List of potential primary serious adverse events.). Primary SAEs include but are not limited to -

Primary Serious Adverse Event	Severity Criteria
**Autonomic dysreflexia (AD)**	A sudden increase in blood pressure, altered heart rate (reflex bradycardia), anxiety, blurred vision, headache, flushing and sweating (above the level of injury). This condition is the likely result of a cervical spinal cord injury. An episode of AD that requires extensive medical treatment and/or hospitalization is considered an SAE.
**Post-thrombic syndrome**	Complication resulting from deep vein thrombosis (DVT) resulting in damage to the veins resulting in reduced blood flow to the legs, leg pain, discoloration and skin sores requiring endovascular or surgical intervention is considered an SAE.
**Pulmonary embolism (PE)**	Complication from a severe case DVT requiring medical attention. Symptoms include shortness of breath, dizziness, chest pain during inhaling or coughing, and rapid heart rate. PE resulting in clot removal surgery or placement of a vein filter is considered an SAE.
**Skin burn**	Prolonged stimulation or excessive leakage current resulting in a second-degree burn underneath the electrode surface. A skin burn requiring major medical intervention is considered an SAE.
**Syringomyelia**	A fluid filled cavity within the spinal cord (finding confirmed through imaging) resulting in weakness in hands and arms, impaired pain and temperature sensation in the back and neck as demonstrated by >2 level ascent in sensory level present on at least 2 sequential neurological exams.
**Pain**	Describes moderate to severe neuropathic or nociceptive pain that requires interventional treatments like local anesthetic blocks, epidural steroid injections etc.
**Urinary tract infection**	An infection of any part of the urinary system (kidneys, ureter, bladder, urethra) that is treated with intravenous antibiotics or results in hospitalization.

Table 10. List of potential primary serious adverse events.

13.5. Anticipated Adverse Events Associated with ARC Therapy

Procedure and device related adverse events related to the use of the LIFT System are defined as having occurred within 7 days of a stimulation session and diagnosed at any time during the follow-up period by the study investigator. A list of expected procedure and device (stimulation) related AEs and their likelihood of occurrence listed in the LIFT User Manual is presented in Table 11 (B.V., 2020).

Anticipated Adverse Event	Definition
**Autonomic dysreflexia (AD)**	A sudden increase in blood pressure, altered heart rate (reflex bradycardia), anxiety, blurred vision, headache, flushing and sweating (above the level of injury). This condition is the likely result of a cervical spinal cord injury. A transient episode of AD that does not require extensive medical treatment and/or hospitalization is considered an AE.
**Bruising from being positioned for therapy sessions**	Medically, bruising is defined as a minor injury in the superficial tissue due to tiny blood vessel rupture and the resulting discoloration requiring minimal medical intervention. In this study, it may occur as subjects rest their hands/arms on a table for a prolonged period of time while doing repetitive movements. The edge of the tables will be padded with towels or foam to minimize this possibility, but some participants with fragile vasculature may occasionally bruise nonetheless.
**Changes in blood pressure**	An increase or decrease in blood pressure (e.g., orthostatic hypotension). This could result from postural changes following prolonged sitting. A transient episode that requires minimal medical attention is considered an AE.
**Deep vein thrombosis (DVT)**	A blood clot in the vein located deep inside the body (e.g., legs) resulting in swelling and/or pain requiring minor medical attention like prescription of blood thinners, compression socks etc.
**Dizziness**	Dizziness or vertigo is the sensation of experiencing unsteadiness, and possibly weakness and fainting. It may be briefly induced by certain stimulation settings but is not expected to persist.
**Minor or brief electric shock**	Tingling or minor jolt experienced in the body. Occurs when subject touches electronic equipment that is not or improperly isolated, incorrect programming, incorrect placement of stimulation electrodes, device malfunction, excessive stimulation or change in skin impedance. A transient episode that is resolved when electrical stimulation is stopped is considered an AE.
**Fall**	A fall resulting in bruise(s) requiring medications and minor medical attention.
**Fracture during training or transportation**	Broken bone as a result of mishaps during subject transportation to/from study site or during the conduct of the therapy/assessment.

**Increase in heart rate**	Increased heart rate above levels expected for ongoing physical activity, as measured by automatic blood pressure cuff or pulse oximeter. Possibly a result of anxiety related to therapy sessions. A transient episode that resolves with conservative medical treatment is considered an AE.
**Muscle or joint strain/discomfort/pain**	Discomfort or mild pain experienced in the muscles or joints. Likely result of multiple FTP sessions in a given week, repetitive motion of the upper extremity and prolonged sitting during therapy sessions with little or no trunk support. If symptoms resolve with exercise or pain medications, it is considered an AE.
**Muscle spasms**	Muscle spasms may result or worsen, if already present, from FTP, ARC Therapy or prolonged sitting during therapy sessions. The condition usually resolves within few hours to 1-2 days after a therapy session.
**Pain**	Describes moderate to severe neuropathic or nociceptive pain that is treated with medications or ceases immediately once a troublesome stimulation parameter is discontinued.
**Patient discomfort**	Mild pain or uneasiness. This condition can result from electric shock (see previous AE), training and/or prolonged sitting during therapy sessions and can subside in a few hours to 1-2 days.
**Post-thrombic syndrome**	Complication resulting from deep vein thrombosis (DVT) resulting in damage to the veins resulting in reduced blood flow to the legs, leg pain, discoloration and skin sores requiring conservative treatment like exercise, blood thinners, lifestyle modifications or compression stockings.
**Pulmonary embolism (PE)**	Complication from a severe case DVT requiring medical attention. Symptoms include shortness of breath, dizziness, chest pain during inhaling or coughing, and rapid heart rate. PE resulting in the use of blood thinners (anticoagulants) or clot dissolvers (thrombolytics) is considered an AE.
**Shortness of breath**	Dyspnea or shortness of breath is a feeling of breathlessness that could result from difficulty in breathing. It also may occur during high aerobic demand during an intense therapy session. A transient episode that resolves with rest is considered an AE.
**Skin rash, irritation, or allergic reaction**	Mild erythema or redness on the skin where electrodes were placed. This condition is caused by repeated application of electrodes over the same skin location. The

	condition will resolve within few hours to 1-2 days with topical applications.
**Swelling of the leg/ankles**	Enlargement of a body part due to fluid accumulation. Results from travel to study site and/or prolonged sitting during the therapy sessions. Resolution of symptoms with stretching and/or other exercises and minimal medical attention is considered an AE.
**Urinary tract infection (UTI)**	An infection of any part of the urinary system (kidneys, ureter, bladder, urethra) that is treated orally with medications including antibiotics.

Table 11. List of some known AEs and their definitions

The above risks will be minimized by follow-up and ongoing observation by members of the research team and the principal investigator. If necessary, subjects maybe referred to appropriate specialists.

13.6. Adverse Device Effects

An adverse device effect (ADE) is defined as any untoward and unintended response to a medical device. This definition includes any event resulting from insufficiencies or inadequacies in instructions for use or deployment of the device. This definition also includes any event that is a result of user error or intentional abnormal use of the device.

13.7. Serious Adverse Device Effects

A serious adverse device effect (SADE) is defined as an ADE that results in any of the consequences characteristic of an SAE or that may lead to any of these consequences if suitable action is not taken or intervention is not made or if circumstances are less opportune.

13.8. Anticipated Serious Adverse Device Effects

An anticipated serious adverse device effect (ASADE) is a serious adverse device effect which by its nature, incidence, severity, or outcome has been identified in the risk analysis report.

13.9. Unanticipated Serious Adverse Device Effects

An unanticipated serious adverse device effect (USADE) is a serious adverse device effect which by its nature, incidence, severity, or outcome has not been identified in the current version of the risk analysis report.

13.10. Handling and Reporting of Adverse Events and Adverse Device Effects

Subjects will be carefully monitored during the study for possible AEs. Any AE will be followed to resolution, until it becomes stable, or until it can be explained by another known cause(s) (i.e., concurrent condition or medication) and clinical judgment indicates that further evaluation is not warranted. All findings relevant to the final outcome of an AE must be reported in the subject’s medical record.

The study investigator will attempt to assess the involvement of the investigational device in the AE. All observations and clinical findings, including the nature, severity, and relationship, will be documented on the appropriate eCRFs.

In case of death, the clinical investigator must make every effort to obtain a copy of the autopsy report and/or death certificate and transmit this to the Sponsor or its designee. The subject’s private information shall be concealed prior to transmittal to the Sponsor. Any other source documents related to the death should also be provided to the Sponsor. In the event that no source documents are available, the PI is required to describe the circumstances of the subject’s death in a letter, e-mail, or other written communication.

The study investigator will report all SAEs to the Sponsor or their designee via email, telephone, or fax immediately but no later than 7 calendar days after first learning of the event, and UADEs/USADEs within 24 hours at the contact information or number provided by the Sponsor. The study investigator will provide a detailed written report within 14 days completing and submitting the applicable eCRF forms with as much information as available at that time. Additionally, the investigator will provide all available supporting documentation (blinded/de-identified as to subject’s identity) to the Sponsor or designee.

As additional information becomes available, the Investigator will record all adverse events (serious or non-serious), adverse device effects (anticipated and unanticipated) and device deficiencies on the appropriate eCRFs. The Sponsor and its designated representatives (e.g., CRO) will be responsible for required regulatory reports of safety in each relevant jurisdiction, as well for timely notification to other study investigators and to the appropriate regulatory agencies as applicable. Furthermore, the Investigator must follow their local IRB policy for AE/SAE/USADE reporting.

Upon receipt of a UADE/USADE from a study site, the Sponsor designee will notify the Sponsor and safety monitor immediately (no later than 24 hours) after receiving notification of an UADE/USADE from the site. The report will initially be reviewed for completeness and legibility before being emailed to the Sponsor and medical monitor for their review of the report package. The medical monitor will make an initial assessment of the safety reporting criteria and determine the requirements for additional follow-up information. The sponsor will review the report package and communicate with the project manager/Medical Monitor and or Principal Investigator if clarifications or additional information about the event is required. All adverse events that meet the criteria for a serious adverse event will be referred to the DSMB for review as described in Section 12.3. All UADE/USADE events will be tracked from initial report and followed through resolution/closure by the sponsor designee. UADE/USADE events will be reconciled against the clinical database and reviewed by the safety monitor/medical monitor as needed.

For any event where there is a suspicion that the device is involved, the Sponsor may request that the study investigator return the device (when possible) for further investigation. The Sponsor will provide a procedure for cleaning and preparation for the return of the devices.

All SAEs must be reported to the IRB who approved the study within 10 days (or sooner if so specified by local IRB policy) after the site becomes aware of the event. All SADEs and UADEs must be reported to the IRB within 10 days after the site is first made aware of the event. All of these reporting requirements are consistent with 21 CFR Part 812.150.

13.11. Severity

The investigator will use the following definitions to determine the severity of an adverse event:

- ***Mild:*** Awareness of a sign or symptom that does not interfere with the subject's usual activity or is transient, resolved without treatment and with no sequelae.
- ***Moderate***: Interferes with the subject's usual activity and/or requires symptomatic treatment.
- ***Severe***: Symptom(s) causing severe discomfort and significant impact of the subject's usual activity and requires treatment.

13.12. Relationship

The investigator will use the following definitions to assess the relationship to the investigational device:

- **Not Related:** Relationship to the device or procedure can be excluded when -

- the event is not a known side effect of the product category the device belongs to or of similar devices and procedures
- the event has no temporal relationship with the use of the investigational device or the procedures
- the serious event does not follow a known response pattern to the medical device (if the response pattern is previously known) and is biologically implausible
- the discontinuation of medical device application or the reduction of the level of activation/exposure, when clinically feasible, and reintroduction of its use (or increase of the level of activation/exposure), do not impact on the serious event
- the event involves a body-site, or an organ not expected to be affected by the device or procedure
- the serious event can be attributed to another cause (e.g., an underlying or concurrent illness/ clinical condition, an effect of another device, drug, treatment, or other risk factors)
- the event does not depend on a false result given by the investigational device used for diagnosis, when applicable
- harms to the subject are not clearly due to use error

In order to establish the non-relatedness, not all the criteria listed above might be met at the same time.

- **Unlikely:** The relationship with the use of the device or procedure seems not relevant and/or the event can be reasonably explained by another cause, but additional information may be obtained.

- **Possible:** The relationship with the use of the investigational device or procedure is weak but cannot be ruled out completely. Alternative causes are also possible (e.g., an underlying or concurrent illness/ clinical condition or/and an effect of another device, drug, or treatment). Cases where relatedness cannot be assessed, or no information has been obtained should also be classified as possible.
- **Probable:** The relationship with the use of the investigational device or procedure seems relevant and/or the event cannot be reasonably explained by another cause, but additional information may be obtained.
- **Causal relationship:** The serious event is associated with the investigational device or with procedures beyond reasonable doubt when-

- the event is a known side effect of the product category the device belongs to or of similar devices and procedures
- the event has a temporal relationship with investigational device use/application or procedures
- the event involves a body-site or organ that
  - the investigational device or procedures are applied to
  - the investigational device or procedures have an effect on
- the serious event follows a known response pattern to the medical device (if the response pattern is previously known)
- the discontinuation of medical device application (or reduction of the level of activation/exposure) and reintroduction of its use (or increase of the level of activation/exposure), impact on the serious event (when clinically feasible)
- other possible causes (e.g., an underlying or concurrent illness/ clinical condition or/and an effect of another device, drug, or treatment) have been adequately ruled out
- harm to the subject is due to error in use
- the event depends on a false result given by the investigational device used for diagnosis, when applicable

In order to establish the relatedness, not all the criteria listed above might be met at the same time.

13.13. Device Malfunction/Failure/Deficiency

Device deficiency is defined as inadequacy of a medical device with respect to its identity, quality, durability, reliability, safety, or performance. Device deficiencies include malfunctions, use errors, and inadequate labeling.

Device malfunction means the failure of the investigational medical device to perform in accordance with its intended purpose when used in accordance with the IFU, IB or Clinical Investigational Plan.

All device deficiencies must be reported to the Sponsor. If a device deficiency results in an adverse event for the subject, this adverse event (AE) must be reported on an AE form of eCRF. All AEs/SAEs associated with a device deficiency/failure are by definition device related.

Any device deficiency that might have led to a SAE if:

- suitable action had not been taken or
- intervention had not been made or
- if circumstances had been less fortunate

will be considered a reportable event and must be handled according to the procedures described in Section 13.10. Device deficiencies that do not result in an adverse event for the subject do not need to be recorded as an AE.

13.14. Early Termination or Suspension of the Clinical Investigation

Based on the advice of an independent DSMB and other factors, the Sponsor may decide to suspend or terminate the study at any time.

Possible reasons for considering study-wide suspension or termination include but are not limited to:

- Adverse events associated with the system or product under investigation which might endanger the safety or welfare of the subjects
- Observed/suspected performance different from the product's intended design
- Decision by the Sponsor or regulatory body, including early completion of study objectives
- Technical issues encountered during the manufacturing process or use of the investigational device.

Possible reasons for clinical investigator or site termination or suspension include but are not limited to:

- Failure to obtain initial IRB approval or annual renewal of the study
- Repeated non-compliance to the CIP (e.g., failure to adhere to inclusion/exclusion criteria, failure to follow subjects per scheduled follow-ups)
- Lack of or insufficient enrollment
- Noncompliance to regulations and/or the terms of the Clinical Trial Agreement (e.g., failure to submit data in a timely manner, failure to follow up on data queries and monitoring findings in a timely manner, etc.)
- IRB suspension of the site
- Fraud or fraudulent misconduct is discovered
- Investigator request (e.g., no longer able to support the study)

13.15. Procedures for Termination or Suspension

Sponsor-initiated and regulatory authority-initiated

- Sponsor will promptly inform the clinical investigators of the termination or suspension and the reasons and inform the Regulatory Authority(ies) where required
- In the case of study termination or suspension for reasons other than a temporary Ethics Committee approval lapse, the investigator will promptly inform the Ethics Committee
- In the case of study termination, the investigator must inform the Subjects and may inform the personal physician of the subjects to ensure appropriate care and follow-up is provided

- In the case of a study suspension, subject enrollment must stop until the suspension is lifted by the Sponsor and approved by the applicable committees/authorities
- In the case of a study suspension, enrolled subjects should continue to be followed out of consideration of their safety, rights, and welfare

Investigator-initiated

- The investigator will inform the Sponsor and provide a detailed written explanation of the termination or suspension
- The investigator will promptly inform the institution (where required per regulatory requirements)
- The investigator will promptly inform the Ethics Committee
- The investigator will promptly inform the subjects and/or the personal physician of the subjects to ensure appropriate care and follow-up is provided
- In the case of a study suspension, subjects enrolled should continue to be followed out of consideration of their safety, rights, and welfare

Institutional Review Board-initiated

- The investigator will inform the Sponsor and provide a detailed written explanation of the termination or suspension within 5 business days
- Subject enrollment must stop until the suspension is lifted
- Subjects already enrolled should continue to be followed in accordance with IRB policy or its determination that an overriding safety concern or ethical issue is involved

14. STATISTICAL ANALYSIS

14.1. Sample Size

The primary effectiveness endpoint is a responder analysis with a responder defined as a subject who demonstrates improvement in at least 1 outcome in each of the strength and function domains. Assuming a minimum power of 80%, a two-sided type I error of 10% (one-sided 5%), a responder rate of 67%, a 50% performance goal and 25% drop out rate, a sample size of 65 subjects is required to be enrolled in the study across a maximum of fifteen sites. Fifty-two of these subjects are intended to complete their four-month visit to ensure that the primary endpoint is statistically powered. Each site can enroll up to a maximum of 25% of the total sample size.

14.2. Statistical Analyses

14.2.1. General Considerations

Except where otherwise specified, the following general principles apply to the planned statistical analyses. All statistical analysis will be conducted using SAS version 9.3 or later (SAS Institute Inc., Cary, NC) or other widely accepted statistical or graphical software as required.

14.2.2. Descriptive Statistics

Continuous data will be summarized with mean, standard deviation, median, minimum, maximum, and number of evaluable observations. Categorical variables will be summarized with frequency counts and percentages. Confidence intervals may be presented, where appropriate, using the t-distribution for continuous data and Clopper-Pearson Exact method for categorical variables.

14.2.3. Study Day

Study Day 0 is the date the subject begins conventional functional task practice (FTP) or FTP and ARC Therapy. Day in study (for each phase) will be calculated relative to Day 0 as follows:

Study Day = Assessment Date – Day 0

For each subject, duration in study will be based on last study contact date which is the latest date of all follow-up visits, assessments, adverse event onset or resolution, and study exit including date of death.

Duration variables will be calculated as follows:

Duration Days = Start Date – End Date

14.2.4. Visit Windows

Unless otherwise specified, visit based assessments will be analyzed for each analysis time point according to the nominal visit entered in the Electronic Case Report Form (eCRF) regardless of if it is out of window.

14.2.5. Statistical Significance

Unless otherwise specified, hypothesis testing will be performed at the one-sided 0.05 significance level. P-values will be rounded to three decimal places. If a p-value is less than 0.001 it will be reported as "<0.001". If a p-value is greater than 0.999, it will be reported as ">0.999".

14.3. Analysis Population

The study will be conducted in 65 enrolled subjects who are least one-year post-cervical spinal cord injury and medically stable. Subjects who sign an informed consent (a designated witness can sign informed consent if the subject is unable to do so) and meet all of the inclusion criteria but none of the exclusion criteria will be considered for treatment. Consented subjects who do not satisfy entry criteria are screen failures and will not receive treatment under this protocol. Those screen failures will conclude their participation in the study without further follow-up or data collection activities.

The following definitions will be applied to the study population.

Intent-to-Treat (ITT) Population: Any subject that is consented and enrolled in the study.

Safety Population: Any subject that is exposed to the study treatment. This is a subset of the ITT population.

Modified Intent-to-Treat (mITT) Population: Subjects who meet ALL of the following criteria are included in the mITT analysis:

- undergo at least 24 of sessions of FTP during the wash-in period
- undergo at least 24 sessions of FTP + ARC Therapy with LIFT during the treatment period

Per-Protocol Population (PPP): Subjects included in the study that met all inclusion criteria and none of the exclusion criteria, were free from major deviations and in which treatment was attempted with the LIFT System.

Safety endpoints are reported in the Safety Population while the study effectiveness endpoints are assessed in the mITT cohort. Additional sensitivity analyses of efficacy measures will be reported in the Safety and PP populations.

Justification for the use of mITT to report effectiveness: The study requires that a subject visit the clinic over 50 times during the course of a 4-month period. During most of these visits, subjects will undergo an intensive FTP regimen. It is reasonable to assume that mobility-impaired tetraplegic subjects may find the number of study visits burdensome due to issues associated with transportation and caregiver availability, amongst many others. This is likely to result in dropouts during the course of the study. Moreover, a minimum number of exposures to both interventions is required to make a fair comparison of the treatment types. Hence, the primary effectiveness endpoint of the study is reported in a population adequately exposed to both therapies. Additional assessments will report dropout rate.

14.4. Subject Disposition

The number of subjects in each analysis population will be presented along with reason for any exclusions. Subject accountability will be summarized by visit. The number of subjects who are enrolled, eligible for follow-up, and number completing FTP and FTP+ARC Therapy will be summarized. In addition, the number of subjects who complete the study or exit early will be summarized by reason.

14.5. Demographics and Baseline Characteristics

Descriptive statistics will be presented for all clinically relevant baseline demographic, medical history, and clinical characteristic variables.

14.6. Primary Endpoints

14.6.1. Primary Safety Endpoint

Observational data regarding the incidence of serious adverse events (SAEs) related to the use of the study device and treatment procedures will be reported. Rationale for the choice of the endpoint is presented in Section 6.5.

No formal statistical hypothesis will be tested. The endpoint will be summarized in the safety analysis set. The number of related SAEs as well as

the number and percent of subjects with one or more related SAE will be reported. The 95% exact confidence interval will be reported.

14.6.2. Primary Effectiveness Endpoint

The primary effectiveness outcome measure will test the hypothesis that a majority of the subjects will experience clinically significant improvement in selected strength and functional performance metrics after treatment with ARC Therapy administered by the LIFT System and FTP. A subject will be considered a treatment responder if she/he reports clinically relevant improvements in at least one outcome each of the Strength and Function domains as follows:

Strength		Function
ISNCSCI-UEMS		GRASSP-Prehension
GRASSP-Strength	and	CUE-T
Pinch force		
Grasp force		

The primary efficacy endpoint will be assessed with the following hypothesis:

H0: $TI \leq 0.50$

Ha: $TI > 0.50$

where TI is the proportion of subjects meeting the responder criteria. The hypothesis will be evaluated using a one-sided statistical test for exact binomial test. The objective will be met if the p-value is < 0.05.

The endpoint will be evaluated using the mITT population which will serve as the Effectiveness Analysis set.

The numerator will include the number of subjects who meet the responder criteria and the denominator will include all subjects evaluable for the primary effectiveness endpoint.

14.7. Secondary Endpoints

14.7.1. Secondary Safety Endpoint

All adverse events (AEs) and SAEs in the study will be reported.

14.7.2. Secondary Effectiveness Endpoint

The following secondary effectiveness endpoints will be tested in descending order of importance through hierarchical testing. In order to maintain the one-sided overall Type I error of 5% the secondary efficacy endpoints will be tested only if the primary objective is met and in the order listed below. Testing will stop if an endpoint is not

met with no subsequent hypotheses tested. Each endpoint will be tested using a one-sided test with Type I error of 5%.

- Superiority of FTP and ARC Therapy with LIFT vs. FTP alone as described by statistically significant difference in responder rates (comparison of change from enrollment baseline to end of FTP with the change from enrollment baseline to end of combined FTP and ARC Therapy with LIFT)
- Quantitative comparison of individual performance metrics to establish superiority of FTP and ARC Therapy with LIFT compared to FTP alone:
    - Pinch force
    - GRASSP-Prehension
    - GRASSP-Strength
    - ISNCSCI-UEMS
    - ISNCSCI-Total sensory score
    - EQ-5D-5L
    - SCIM
    - WHOQOL-BREF

14.8. Sensitivity Analyses

14.8.1. Primary Endpoint

Sensitivity analyses for the primary effectiveness endpoint will be performed on the following alternate populations:

- Analysis including all subjects enrolled in the study (ITT population)
- Analysis including all subjects exposed to the ARC Therapy (Safety population)
- Analysis restricted to subjects who meet all of the inclusion criteria and none of the exclusion criteria and were free from major deviations, who completed all required therapy sessions and in which treatment was attempted with the LIFT System (PP population)
- The analysis to determine MIDs in the primary effectiveness composite will be repeated with final data. The purpose will be to validate the levels chosen for the primary effectiveness endpoint and to provide a supportive, post-hoc, sensitivity re-analysis of the primary endpoint using the study data derived MIDs.
- An additional analysis of MIDs in the primary effectiveness composite outcomes will use the Patient Global Impression of Change (PGIC) for a post-hoc determination of MID change. This will involve a re-analysis of the primary effectiveness endpoints using the MID criteria established through this analysis. Correlation between the PGIC and each outcome measure will be calculated. With a response of minimally improved or better on the PGIC defined as clinically meaningful improvement, the change in each outcome measure will be summarized among subjects meeting the PGIC clinically

meaningful improvement with the mean response defined as the MID. This will provide an additional supportive, post-hoc, sensitivity re-analysis of the primary endpoint using study data derived MIDs.

14.8.2. Validation of Minimal Important Difference (MID) Criterion

The Global Impression of Change (GIC) survey is designed to assess changes in symptoms associated with study defined treatments. The MIDs used to define a responder in the study are based on pilot study data from 3 sites with up to 13 subjects. However, the variation in the data set was large in part due to the small sample size and dissimilar treatment protocol followed by the sites. It is expected that reduction of symptoms will be correlated with improved performance in one or more primary outcome measures. To validate the initial selection of MID criteria, a sensitivity analysis of the primary endpoint will be performed using GIC results (minimally improved or better) to assess responder rate as compared to that determined using Cohen's small effect size.

14.9. Poolability Analyses

All investigational sites will follow the requirements of a common protocol and standardized data collection procedures and forms. The primary endpoints will be presented separately for each site using descriptive statistics. Poolability of the primary endpoints across investigational sites will be evaluated using a chi-square test. If the p-value for the interaction effect is <0.15, additional exploratory analyses will be performed to understand any variations in outcomes by site.

14.10. Safety Analyses

Adverse events (AE) will be reported for the Safety Analysis set population. AEs will be tabulated with the number of events and subjects with event for each event type and overall. Rates will be reported as the number of subjects who experience at least one event during the analysis interval out of the total number of subjects with follow-up to the beginning of the analysis interval. Serious adverse events will also be tabulated.

All AEs and SAEs will also be summarized by relatedness as described above. Adverse events leading to death or study discontinuation will be provided in listing format.

All device deficiencies will be reported in listing format.

14.11. Interim Analyses

No interim analyses are planned.

14.12. Protocol Deviations

Deviations from the procedures outlined in the CIP will be reported by investigational sites on the eCRF. Protocol deviations will be summarized for all deviations and by type with event counts and number of subjects with at least one deviation.

14.13. Other Analyses

Descriptive statistics on additional domains in the primary endpoint outcomes will be reported. The following additional assessments impacting subject quality of life and long-term consequences of SCI such as outcomes related to pain, spasticity, quality of life, blood pressure and bladder/bowel/sexual function will be reported as descriptive statistics and clinically relevant changes, where possible.

- Numerical Rating Scale (NRS) for pain
- International Spinal Cord Injury Pain Data Set (ISCIPDS)
- Medical Outcomes Study (MOS) Sleep Scale
- Spinal Cord Independent Measure (SCIM III)
- Penn Spasm Frequency Scale (PSFS)
- EuroQol 5 Dimension 5 Level Questionnaire (EQ-5D-5L)
- World Health Organization Quality of Life (WHOQOL-BREF)
- International Standards to document remaining Autonomic Function after Spinal Cord Injury (ISAFSCI)
- Patient Health Questionnaire (PHQ-9)
- Global Impression of Change (Clinician and Patient)

14.14. Follow-up Analyses

Whereas the primary and secondary effectiveness endpoints reflect on the role of ARC Therapy to promote neuroplasticity and sustained performance gains without active stimulation, it is expected that some subjects may only show treatment benefit when the stimulation is active. Subjects who respond to active ARC Therapy but do not sustain these gains with the stimulator off may be indicated for extended therapy or other modes of delivering ARC Therapy, e.g. implantable systems.

The follow-up assessment is uniquely performed with the stimulator first off, then on, to allow for evaluation of the immediate neuroprosthetic effects of active ARC Therapy.

14.15. Changes from Planned Analyses

Any changes to planned statistical analyses determined necessary prior to performing the analyses will be documented in an amended Statistical Analysis Plan and approved prior to the analysis when possible. Any other deviations or changes from the planned analyses deemed necessary due to violation of critical underlying statistical assumptions, data characteristics, or missing data will be clearly described in the clinical study report with justification and rationale.

14.16. Subject Listings

Subject listings will be provided for all adverse events and primary and secondary effectiveness outcomes, adverse events, and protocol deviations.

14.17. Missing Data

All attempts will be made to limit the amount of missing data. Unless otherwise specified, no attempt will be made to impute missing data. If a data point is missing,

that data point will not contribute to that portion of the analysis. The number of evaluable observations will be reported in analysis so that extent of missing data can be assessed.

14.17. Early Termination Evaluation

No formal early termination procedures will be conducted. Subjects who withdraw before completing the study will be followed only through the date of their withdrawal or through resolution or stabilization of the AE, in case of an ongoing event.

14.18. Subgroup Analysis

It is possible that subgroup analyses will be performed for a subset of the endpoints or subject cohorts (e.g., AIS C) as exploratory analyses.

15. DATA QUALITY ASSURANCE

The study described in this CIP will be implemented according to the FDA's regulatory requirements and GCP. All procedures not described in this protocol will be performed according to approved written Standard Operating Procedures (SOPs) unless otherwise stated.

Steps to assure the accuracy and reliability of data include the selection of qualified clinical investigators and appropriate study sites, review of CIP procedures with the clinical investigator and associated personnel prior to the study, and periodic monitoring visits by the Sponsor or its designee. The Sponsor or its designee will review data for accuracy and completeness during periodic remote and/or on-site monitoring visits, and any discrepancies will be resolved with the clinical investigator or designees as appropriate.

15.1. Handling and Storage of Data and Documents

The investigators must maintain adequate and accurate records to document the conduct of the study and substantiate the study data. These documents include those required by applicable regulations, and the subjects' source documents, as described below.

Regulatory documents are those documents that individually and collectively permit evaluation of the study compliance with applicable regulations and the quality of the data produced. These documents will be filed in a Regulatory Binder provided by the Sponsor or designee. This file shall be used to facilitate and ensure filing of all relevant regulatory documents during and after the study. The investigator will be responsible for keeping the Investigator's Study File updated and ensuring that all required documents are filed. The file will be inspected during monitoring visits.

Source documents are original hospital records, clinical charts, screening log, subject identification lists, enrollment logs, original laboratory reports, memoranda, pharmacy dispensing records, recorded data from automated instruments, transcriptions certified after verification as being accurate, magnetic, or electronic media, x-rays, subject's files, and records kept at pharmacy, laboratories and medicotechnical departments involved in the study. The investigator must maintain source documents for each subject in the study.

All information recorded on the eCRFs must be traceable to these source documents. The investigator shall arrange for the retention of all study documents and records, including subject records, eCRFs, drug inventory/accountability logs, signed informed consent forms and the subject identification list, after completion or discontinuation of the study for the minimum period as required per local regulation.

15.2. Case Report Forms

Clinical data are collected at designated time points throughout the study. Electronic Case Report Forms (eCRFs) will be used to collect all study subject data during the course of the study. It is the study investigator's responsibility to ensure accurate and timely completion of the eCRFs and to approve the eCRFs. The clinical investigator recognized by the IRB has the authority to sign eCRFs. These electronic signatures serve to attest that the information contained in the eCRF is accurate and true.

Optionally, data may be stored in a secure, password-protected database which will be backed up periodically. Data will be reviewed using programmed and manual data checks.

The eCRFs contain confidential material. Specific training on the completion of the forms will be provided to the clinical investigator and other site personnel as appropriate. The clinical investigator is responsible for the accuracy and completeness of data reported on eCRFs.

Data queries will be made available to study sites for resolution. The data monitor will periodically verify the data entered onto the eCRFs against source documents to ensure data integrity, accuracy, and completeness of the data prior to locking the eCRFs for tabulation of study endpoint data. Study management reports may be generated by the Sponsor (or delegate) to monitor data quality and study progress. At the end of the study, the data will be frozen and retained indefinitely by the Sponsor for a period of five (5) years, or as required by local regulation.

Good Clinical Practice Guidelines require that Investigators maintain information in the study subject's medical records that corroborate data collected in the eCRFs.

The Investigator or designated individual shall be responsible for recording all study data on the eCRFs provided by the Sponsor. The Investigator is required to sign the eCRF on the appropriate page(s) to verify that he/she has reviewed and agrees with the recorded data.

Elements of eCRFs will be adopted from NINDS' CDE for SCI research (NINDS: CDE for SCI Research, 2020).

15.3. Deviations

The investigator is not allowed to deviate from the Clinical Investigational Plan, except to maintain the subject's rights, safety and well-being or the scientific integrity of the investigation. All protocol deviations shall be documented and a justification for any missed or tardy assessments shall be provided on the Protocol Deviation Case Report Form.

The Sponsor (or delegate) is responsible for analyzing deviations, assessing their significance, and identifying any additional corrective and/or preventive actions (e.g.,

amend the Clinical Investigational Plan, conduct additional training, or terminate the investigation). Repetitive or serious investigator compliance issues may result in initiation of a corrective action plan with the investigator and site, and in some cases, necessitate suspending enrollment until the problem is resolved or ultimately terminating the investigator's participation in the study. Sponsor (or delegate) will provide site-specific reports to investigators summarizing information on deviations that occurred at the investigational site on a periodic basis.

15.4. Monitoring Procedures and Visits

The Sponsor or its designee will conduct site visits to the study facilities to monitor the study and ensure compliance with the CIP, GCP, and applicable regulations and guidelines. A detailed monitoring plan will be developed outlining the details.

The clinical investigator agrees to allow these monitors and other authorized Sponsor representatives access to the site, and to study documentation for the above-mentioned purpose and agrees to assist the monitors in their activities, if requested. Completed eCRFs will be verified by the appointed monitor at the investigational site at regular intervals throughout the study. Missing or unclear data will be investigated by the monitor and will be retrieved and clarified by study personnel as necessary throughout the study. The Sponsor or their authorized representative, may request additional documentation from the investigator such as physician procedure notes or physician written summaries when adverse events are observed and reported.

Requests by regulatory agencies to inspect the study sites may be made. The clinical investigator agrees to allow inspectors from regulatory agencies to review records and to assist the inspectors in their duties, if requested.

Frequency of monitoring visits may be based upon subject enrollment, duration of the study, study compliance, number of adverse events, number of deviations, findings from previous monitoring visits and any suspected inconsistency in data that requires investigation. Regulatory documents may be reviewed at each study site. Monitoring for the study will be done in accordance with the study monitoring plan.

Monitoring visits may be conducted periodically to assess site study progress, the investigator's adherence to the Clinical Investigational Plan, regulatory compliance, maintenance of records and review of source documents against subject eCRFs. Monitors review site regulatory and study compliance by identifying findings of non-compliance and communicating those findings along with recommendations for preventative/corrective actions to site personnel. Monitors may work with study personnel to determine appropriate corrective action recommendations and to identify trends within the study or at a particular site.

15.5. Clinical Investigational Plan Amendments

During the course of the study, a revision or an amendment to the Clinical Investigational Plan may be necessary. Any amendment, including justification for the modification, must be submitted to, and approved by the central and/or study site's Institutional Review Board and relevant Regulatory Authority prior to implementation of the amendment, unless the modifications increase Subject safety.

Any revisions or amendment(s) that affect the informed consent form require a revised Sponsor and IRB approved informed consent form before changes in study procedures are implemented. These requirements should in no way prevent any immediate action from being taken by the Investigator or by the Sponsor to preserve the safety of any Subjects included in the study, as necessary. If an immediate change to the Clinical Investigational Plan is felt to be necessary by the Investigator and is implemented by him/her for safety reasons, the Sponsor should be immediately notified.

15.6. Data Management

The standard procedures for handling and processing records will be followed per GCP and the Sponsor or its designee's SOPs. A comprehensive Data Management Plan will be developed including a Data Management Overview, Database Contents, annotated eCRF, Pre-Entry Review List, Self-evident Correction Conventions, Query Contacts, and Consistency Checks.

15.7. Device Accountability

All investigational study devices will be labeled as "Investigational Device" and will only be used for subjects enrolled in this study. The investigational study devices must be stored in a locked/secured storage facility to which only the Investigator and/or designated study staff will have access. The Investigator must ensure that the device is used only in accordance with the Clinical Investigational Plan, IFU and IB.

The Sponsor will maintain records of all investigational devices shipped to the investigational site. Investigators will be responsible for maintaining records of devices received, verification of shipping information (e.g., lot numbers and quantities), the date of the procedure on which each device was used, and final device disposition (e.g., destroyed, returned to sponsor, etc.).

All complaints of a non-medical nature must be documented and will be handled under the Sponsor's quality management system. In the case where a device has failed, the Investigator must make every possible effort to return the device to the Sponsor according to the provided instructions.

15.8. Study Reports

The Sponsor/investigator may submit a summary of the progress of the study to the involved IRBs once a year or according to the local requirements. Information will be provided on the date of inclusion of the first subject, numbers of subjects included and numbers of subjects that have completed the study, serious adverse events/ serious adverse reactions, UADEs, device deficiencies, protocol deviations, revisions, and amendments.

The Sponsor will notify the involved IRBs and the Regulatory Authority of the end of the study within a period of 90 days. The end of the study is defined as the last subject's last visit.

In case the study is ended prematurely, the Sponsor will notify the involved IRB and the Regulatory Authority within 15 days, including the reasons for the premature termination.

If required by the regulations, the investigator/Sponsor will submit a final study report with the results of the study, including any publications/abstracts of the study, to the involved IRB and the Regulatory Authority within one year from the end of the study.

16. ETHICS

16.1. Declaration of Helsinki

The study will be conducted according to the guidelines established in the Declaration of Helsinki. Subject can withdraw from the study at any time without prejudice to their subsequent treatment.

16.2. Institutional Review Board or Independent Ethics Committee Approval

This study will be conducted in compliance with the Declaration of Helsinki and its amendments and the applicable regulations of the country in which the study is conducted.

An Institutional Review Board (IRB) or local Ethics Committee (EC) must review and approve the CIP, the investigator's informed consent document, and related subject information and recruitment materials before the start of the study.

16.3. Informed Consent

Informed consent shall be obtained in writing and documented before a patient is enrolled in the clinical investigation in accordance with the principles of Informed Consent, according to the Declaration of Helsinki, Good Clinical Practice (GCP), 21 Code of Federal Regulations (CFR) Part 50, the Medical Devices Directive 93/42/EEC, and International Organization for Standardization (ISO) 14155-1, Chapter 6.7.1.

It is the responsibility of the study investigator to ensure that written informed consent is obtained from the patient (or legally acceptable representative) before any activity or procedure is undertaken that is not part of routine care.

16.4. Subject Identification and Confidentiality

Subject identification and confidentiality will be ensured according to the terms and definitions in ISO 14155-1, Chapter 6.5. This includes but is not limited to the following:

a. Subjects will be identified on all eCRFs by a unique reference code including their initials.
b. eCRFs are confidential documents and will only be available to the Sponsor or its delegates (e.g., CRO), the study site personnel, the biostatistician, and if requested, the DSMB and regulatory authorities. The principal investigator for each site will maintain, as part of the investigation file, a list identifying all subjects entered into the trial.

17. REGULATORY REQUIREMENTS

17.1. Compliance with Regulations Applicable to Clinical Trials

The clinical trial will be conducted, and data generated, documented, and reported in compliance with the Clinical Investigational Protocol, Good Clinical Practice (GCP) and the applicable regulatory requirements. The investigation will be performed in accordance with the ethical principles from the most recent version of the Declaration of Helsinki.

17.1.1. Standards

The most recent version of ISO 14155 will be followed in addition to national regulations, 21 CFR 50 Protection of Human Subjects, 21 CFR 56 Institutional Review Boards and 21 CFR 54 Financial Disclosure.

17.1.2. Regulatory Documents

The following regulatory documents will be collected before the initiation of the study:

- A signed and dated Investigator Agreement
- A copy of the IRB (Ethics Committee) approval for the Protocol
- A current curriculum vitae and if applicable, a medical license of the Investigator(s)
- A signed financial disclosure statement for Investigators participating in the Study
- Verification that the IRB (Ethics Committee) is properly composed
- A copy of the IRB (Ethics Committee) approval of the Informed Consent Form
- A signed and dated Financial Contract
- Delegation of authority, if applicable

When amendments are issued to any of the above documents, they will be filed accordingly.

17.1.3. Subject Data Protection

The clinical study will ensure that the rights and wellbeing of trial subjects are protected and that the reported trial data are accurate, complete, and verifiable.

The Principal Investigator will ensure that the subject is given full and adequate oral and written information about the nature, purpose, possible risks, and benefits of the investigation. Subjects will also be notified that they are free to discontinue participation in the investigation at any time. The subject will be given the opportunity to ask questions and time for consideration.

The subject's signed informed consent will be obtained before conducting any investigation related procedures. The original must be filed by the Principal Investigator. A copy of the Patient Information including the signed Consent Form will be given to the subject.

17.1.4. Adverse Event Monitoring and Reporting

Investigator will notify of serious adverse events and related reports. These unanticipated problems need to be reported promptly to the IRB, the Sponsor and federal agency because they may involve greater risks to human subjects or others than previously expected.

18. CHANGES TO THE CIP OR RELATED PROCEDURES

This CIP cannot be altered or changed except through a formal CIP amendment, which requires the written approval of the Sponsor and approved by the IRB before implementation.

19. DEVIATIONS FROM THE CIP

Any deviation from the CIP must be recorded along with an explanation for the deviation. The study investigator will record each deviation using the appropriate CRF within 14 working days. As soon as the site becomes aware of it, significant deviations will be immediately reported to the Sponsor and the IRB within the appropriate deadlines stipulated by the appropriate regulatory agency(s) and according to local SOPs.

Significant deviations are defined as those impacting or potentially impacting subject safety, such as enrollment of non-eligible patients, and any deviation that significantly compromises the outcome of the study.

20. GENERAL CONSIDERATIONS

20.1. Discontinuation of the Study

The Sponsor reserves the right to discontinue this study for safety or administrative reasons at any time. In such events, subjects will be followed to assess safety through the entire planned follow-up period.

20.2. Use of Information and Publication

All information concerning the LIFT device, patent applications, manufacturing processes, and basic scientific data supplied by the Sponsor or its designee to the research site, study investigator and research staff and not previously published, is considered confidential and remains the sole property of the Sponsor. The eCRFs will also remain the property of the Sponsor. The study investigator agrees to use this information for purposes of study execution through finalization.

The information developed in this study will be used by the Sponsor in connection with the continued development of the LIFT device and thus may be disclosed as required to other study investigators or government regulatory agencies.

All parties agree that the Sponsor in collaboration with the investigators will produce publications or presentations. The number of authors will be determined according to the rules of the addressed scientific journal and by decision of the Sponsor.

Investigators can publish their site-specific data only after the study data in its entirety is published. Abstracts, manuscripts, and conference presentations shall be submitted to the Sponsor in advance of their publication. An agreement on the final form of

abstracts, manuscripts and presentations shall be obtained within an appropriate time frame of 30 days.

The Sponsor and Investigators will agree that disclosure of study data may be constrained at any time by regulatory concerns or when premature disclosure of partial study information would bias the interpretation of study findings. Any and all information supplied or obtained during this study by or on behalf of any party involved in the study (in whatever form) shall be treated as confidential and shall not be disclosed to any third party unless with the prior written consent of the Sponsor in each case. Any documents, papers, drawings, or other materials which are released or created by any party involved in this study are and shall remain at all times the property of the Sponsor excluding publications which are approved in writing by the Sponsor. Such materials shall not be reproduced in any form without the prior written consent of the Sponsor and must be returned to the Sponsor immediately upon request, or upon completion of the evaluation of such materials, whichever is the earlier.

All clinical data and any other information collected during or after the study, as well as information associated with the people or materials involved, will be considered confidential. The study will be registered on www.clinicaltrials.gov and enrollment/publication status will be updated as required by regulations.

21. REFERENCES

Adams, M., Ginis, K., & Hicks, A. (2007). The spinal cord injury spasticity evaluation tool: development and evaluation. *Arch Phys Med Rehabil*, 88(9):1185-1192.

Administration, U. F. (2006.). *Guidance for Clinical Trial Sponsors. Establishment and Operation of Clinical Trial Data Monitoring Committees.*

Anderson, K. (2004). Targeting recovery: priorities of the spinal cord-injured population. *J Neurotrauma*, 21(10):1371–83. doi:10.1089/neu.2004.21.1371.

B.V., G. m. (2020, May). Noninvasive Electrical Spinal Stimulation User Manual for Clinical Research Staff (Beta Prototype).

Beekhuizen, K., & Field-Fote, E. (2005). Massed Practice versus Massed Practice with Stimulation: Effects on Upper Extremity Function and Cortical Plasticity in Individuals with Incomplete Cervical Spinal Cord Injury. *Neurorehabil Neural Repair*, 19:33-45.

Beekhuizen, K., & Field-Fote, E. (2008). Sensory Stimulation Augments the Effects of Massed Practice Training in Persons With Tetraplegia. *Arch Phys Med Rehabili*, 602-08.

Benevento, B., & Sipski, M. (2002). Neurogenic bladder, neurogenic bowel, and sexual dysfunction in people with spinal cord injury. *Phys Ther*, 82: 601-612 [PMID: 12036401].

Bonniaud, V., Bryant, D., Parratte, B., & Guyatt, G. (2008). Development and validation of the short form of a urinary quality of life questionnaire: SF-Qualiveen. *J Urol*, 180(6):2592–259.

Brazier, J., Harper, N., Jones, A., O'Cathain, K., Thomas, T., Usherwood, T., & Westlake, L. (1992). Validating the SF-36 health questionnaire: A new outcome measure for primary care. *Br Med J*, 305: 160-.

Buysse, D., Reynold, C., Monk, T., Berman, S., & Kupfer, D. (1989). The Pittsburgh Sleep Quality Index: A New Instrument for Psychiatric Practice and Research. *Psychiatry Res*, 28(2): 193-213.

Catz, A., Itzkovich, M., Agranov, E., Ring, H., & Tamir, A. (1997). SCIM-spinal cord independence measure: a new disability scale for patients with spinal cord lesions. *Spinal Cord*, 35: 850–856.

Charlifue, S., Post, M., Biering-Sørensen, F., Catz, A., Dijkers, M., Geyh, S., . . . Sinnott, K. (2012). A multi-purpose survey designed to capture adult patients' perceptions of their own health and well-being. . *Spinal Cord*, 50(9): 672-5.

Clark, B., Mahato, N., Nakazawa, M., Law, T., & Thomas, J. (2014). The power of mind: the cortex as a critical determinant of muscle strength/weakness. *J Neurophysiol*, 112(12): 3219-26.

Cohen, J. (1988). *Statistical Power Analysis for the Behavioral Sciences.* New York: Lawrence Erlbaum Associates.

Darian-Smith, I., Galea, M., & Draian-Smith, C. (1996). Manual dexterity: how does the cerebral cortex contribute? *Clin Exp Pharmacol Physiol*, 23(10-11): 948-56.

Davari, M., Amani, B., Amani, B., Khanijahani, A., & Akbarzadeh, A. S. (2020). Pregabalin and gabapentin in neuropathic pain management after spinal cord injury: a systematic review and meta-analysis. *Korean J Pain*, 33(1): 3-12.

Dietz, V., & Fouad, K. (2014). Restoration of sensorimotor functions after spinal cord injury. *Brain*, 137(1): 654-667.

Dimitrijevic, M., Dimitrijevic, M., Illis, L., Nakajima, K., Sharkey, P., & Sherwood, A. (1986). Spinal cord stimulation for the control of spasticity in patients with chronic spinal cord injury: I. Clinical observations. *Cent Nerv Sys Trauma*, 3: 129-44.

Dimitrijevic, M., Illis, L., Nakajima, K., Sharkey, P., & Sherwood, A. (1986). Spinal cord stimulation for the control of spasticity in patients with chronic spinal cord injury: II. Neurophysiologic observations. *Cent Nerv Syst Trauma.* , 3:145-152.

Edgerton, V., & Roy, R. (2012). A new age for rehabilitation. *Eur J Phys Rehabil Med*, 48(1): 99–109.

FDA. (2006, January). *Significant Risk and Nonsignificant Risk Medical Device Studies: Guidance For IRBs, Clinical Investigators, and Sponsors*. Retrieved from https://www.fda.gov/regulatory-information/search-fda-guidance-documents/significant-risk-and-nonsignificant-risk-medical-device-studies

Friden, J., & Gohritz, A. (2015). Tetraplegia Management Update. *J Hand Surg Am*, 40(12): 2489-2500.

Gad, P., Lee, S., Terrafranca, N., Zhing, H., Turner, A., Gerasimenko, Y., & Edgerton, V. (2018). Non-Invasive Activation of Cervical Spinal Networks after Severe Paralysis. *J Neurotrauma*, 35: 2145-58.

Gomes-Osman, J., Tibbett, J., Poe, B., & Field-Fote, E. (2017). Priming for Improved Hand Strength in Persons with Chronic Tetraplegia: A Comparison of Priming-Augmented Functional Task Practice, Priming Alone, and Conventional Exercise Training. *Frontiers in Neurology*, 7:242.

Gorgey, A., Chiodo, A., Zemper, E., Hornyak, J., Rodriguez, G., & Gater, D. (2010). Relationship of spasticity to soft tissue body composition and the metabolic profile in persons with chronic motor complete spinal cord injury. *J Spinal Cord Med*, 33: 6-15.

Graves, D., Frankiewicz, R., & Donovan, W. (2006). Construct Validity and Dimensional Structure of the ASIA Motor Scale. *J Spinal Cord Med.* , 29:39–45.

Hays, R. &. (1992). Sleep measures. In A. &. Stewart, *Measuring functioning and well-being: The Medical Outcomes Study approach* (pp. 235-259). Durham, NC: Duke University Press.

Hays, R., & Stewart, A. (1992). Sleep measures. In A. Stewart, & J. Ware, *Measuring Functioning and Well-being: The Medical Outcomes Study Approach.* (pp. 235-259). Durham, NC: Duke University Press.

Hays, R., Martin, S., Sesti, A., & Spritzer, K. (2005). Psychometric properties of the Medical Outcomes Study Sleep measure. *Sleep Med*, 6(1):41-4.

Herdman, M., Gudex, C., Lloyd, A., Janssen, M., Kind, P., Parkin, D., . . . Badia, X. (2011). Development and preliminary testing of the new five-level version of EQ-5D (EQ-5D-5L). . *Quality of Life Research*, 20(10):1727-36.

Hoffman, L., & Field-Fote, E. (2007). Cortical reorganization following bimanual training and somatosensory stimulation in cervical spinal cord injury: a case report. *Phys Ther*, 87:208-23.

Hoffman, L., & Field-Fote, E. (2013). Effects of Practice Combined with Somatosensory or Motor Stimulation on Hand Function in Persons with Spinal Cord Injury. *Top Spinal Cord Inj Rehabil*, 19(4): 288-99.

Inanici, F., Samejima, S., Gad, P., Edgerto, V., Hofstetter, C., & Moritz, C. (2018). Transcutaneous Electrical Spinal Stimulation Promotes Long-Term Recovery of Upper Extremity Function in Chronic Tetraplegia. *IEEE Trans Neural Sys and Rehabili Engg*, 26(6): 1272-78.

Jang, Y., Hsieh, C.-L., Wang, Y.-H., & Wu, Y.-H. (2004). A Validity Study of the WHOQOL-BREF Assessment in Persons With Traumatic Spinal Cord Injury. *Arch Phys Med Rehabil*, 85(11):1890-5.

Kalsi-Ryan, S., Beaton, D., Curt, A., Duff, S., & Popovic, M. (2012). The graded redefined assessment of strength sensibility and prehension: reliability and validity. *J Neurotrauma*, 905-14.

Krassioukov, A., Biering-Sorensen, F., Donovan, W., Kennelly, M., Kirshblum, S., Krogh, K., . . . Wecht, J. (2012). International Standards to document remaining Autonomic Function after Spinal Cord Injury (ISAFSCI). *Top Spinal Cord Inj Rehabil*, 282-296.

Krassioukov, A., Eng, J., Claxton, G., Sakakibara, B., & Shum, S. (2010). S. Neurogenic bowel management after spinal cord injury: a systematic review of the evidence. *Spinal Cord*, 48: 718-33.

Krassioukov, A., Eng, J., Warburton, D., & Teasell, R. (2009). A systematic review of the management of orthostatic hyoptension after spinal cord injury. *Arch Phys Med Rehabil*, 90: 876-885.

Kroenke, K., Spitzer, R., & Williams, J. (2001). The PHQ-9: Validity of a Brief Depression Severity Measure. *J Gen Intern Med*, 16(9): 606-13.

Krogh, K., Christensen, P., Sabroe, S., & Laurberg, S. (2006). Neurogenic bowel dysfunction score. *Spinal Cord*, (44): 625-631.

Liu, C., Huang, C., Yang, Y., Chen, S., Weng, M., & Huang, M. (2009). Relationship between neurogenic bowel dysfunction and health-related quality of life in persons with spinal cord injury. *J Rehabil Med*, 41: 35-40.

Lo, C., Tran, Y., Anderson, K., Craig, A., & Middleton, J. (2016). Functional priorities in persons with spinal cord injury: using discrete choice experiments to determine preferences. *J Neurotrauma*, 33(21):1958–68. doi:10.1089/ neu.2016.4423.

Lu, D., Edgerton, V., Modaber, M., AuYong, N., Morikawa, E., Zdunowski, S., . . . Gerasimenko, Y. (2016). Engaging cervical spinal cord networks to re-enable volitional control of hand function in tetraplegic patients. *Neurorehabil Neural Repair*, 30(10): 951-62.

Marino, R., Kern, S., Leiby, B., Mary Schmidt-Read, M., & Mulcahey, M. (2015). Reliability and validity of the capabilities of upper extremity test (CUE-T) in subjects with chronic spinal cord injury. *J Spinal Cord Med*, 38(4): 498-504.

Mehta, S., McIntrye, A., Dijkers, M., Loh, E., & Teasell, R. (2014). Gabapentinoids are effective in decreasing neuropathic pain and other secondary outcomes after spinal cord injury: a meta-analysis. *Arch Phys Med Rehabil*, 95(11): 2180-6.

(2016). *National Spinal Cord Injury Statistical Center (NSCISC). Spinal Cord Injury Facts and Figures at a Glance. Available from: https://www. nscisc.uab.edu/Public/Facts%202016.pdf*.

NINDS: CDE for SCI Research. (2020). Retrieved from https://www.commondataelements.ninds.nih.gov/Spinal%20Cord%20Injury

Rekand, T., Hagen, E., & Grønning, M. (2012). Spasticity following spinal cord injury. *Tidsskr Nor Laegeforen*, 132: 970-973.

Rosen, R., Brown, C., Heiman, J., Leiblum, S., Meston, C., Shabsigh, R., . . . D'Agostino Jr., R. (2000). The Female Sexual Function Index (FSFI): A Multidimensional Self-Report Instrument for the Assessment of Female Sexual Function. *J Sex & Marital Therapy*, 26: 191-208.

Rosen, R., Riley, A., Wagner, G., Osterloh, I., Kirkpatrick, J., & Mishra, A. (1997). The International Index of Erectile Function (IIEF): A multidimensional scale for assessment of erectile dysfunction. . *Urology*, 49: 822-830.

Sezer, N., Akkuş, S., & Uğurlu, F. G. (2015). Chronic complications of spinal cord injury. *World J Orthop*, January 18; 6(1): 24-33.

Siddall, P., & Middleton, J. (2006). A proposed algorithm for the management of pain following spinal cord injury. *Spinal Cord*, 44: 67-77.

Smaby, N., Johanson, M., Baker, B., Kenney, D., Murray, W., & Hentz, V. (2004). Identification of key pinch forces required to complete functional tasks. . *J Rehabil Res Dev* , 41(2):215–24. doi:10.1682/JRRD.2004.02.0215.

Snoek, G., IJzerman, M., Hermens, H., Maxwell, D., & Biering-Sorensen, F. (2004). Survey of the needs of patients with spinal cord injury: impact and priority for improvement in hand function in tetraplegics. . *Spinal Cord*, 42:526–32. doi:10.1038/sj.sc.3101638 .

Stiens, S., Bergman, S., & Goetz, L. (1997). Neurogenic bowel dysfunction after spinal cord injury: clinical evaluation and rehabilitative management. *Arch Phys Med Rehabil*, 78: S86-102.

Waltz, J., Andreesen, W., & Hunt, D. (1987). Spinal cord stimulation and motor disorders. *Pacing Clin Electrophysiol*, 10(1): 180-204.

Weaver, T., Laizner, A., Evans, L., Maislin, G., Chugh, D., Lyon, K., . . . Dinges, D. (1997). An instrument to measure functional status outcomes for disorders of excessive sleepiness. *Sleep*, 20: 835-43.

Widerström-Noga, E. B.-S., Bryce, T., Cardenas, D., Finnerup, N., Jensen, M., Richards, S., & PJ., S. (2008). The International Spinal Cord Injury Pain Basic Data Set. *Spinal Cord*, 46: 818-23.

Wu, X., Liu, J., Tanadini, L., Lammertse, D., Blight, A., Kramer, J., . . . Steeves, J. (2015). Challenges for defining minimal clinically important difference (MCID) after spinal cord injury. *Spinal Cord*, 84-91.

22. APPENDICES

22.1. Appendix A. Data Safety Monitoring Board (DSMB)

The Sponsor will establish an independent data safety monitoring board (DSMB) comprised of clinicians and/or researchers recognized as subject matter experts in the field of spinal cord injury. The DSMB will meet periodically throughout the study to review study safety and advise the Sponsor on safe conduct of the study. Committee members must possess prior experience working in a DSMB or reviewing AEs as recommended by FDA (Administration, 2006.). The following elements are described fully in committee's charter:

- The DSMB will be responsible for reviewing all SAEs including SAEs, ADEs, UADEs reported during the study, as identified, and submitted by the Sponsor or its designee during the course of the Up-LIFT Study.
- This committee will be comprised of 3 physicians and/or researchers who are subject matter experts in SCI and/or have experience in clinical trial safety management.
- Members of DSMB are not participants in the study and meet periodically throughout the study to review events in an ongoing fashion. If the member's site is a trial participant, he/she will not be the site PI.
- The Sponsor or its designee will collate the clinical events along with a detailed narrative from the site including relevant copies of source documents and send to the members for review ahead of the meeting.
- After review of the identified events by the DSMB, reporting may be deferred pending additional supporting documentation as requested from the site.
- The results of the review (including device or procedure relatedness, if applicable) are entered into the DSMB minutes after the meeting. In case of an UADE that is deemed unacceptable or introduces untenable risk to subjects' health, the DSMB can recommend that the Sponsor suspend or terminate the study. However, the final decision regarding study suspension or termination rests with the Sponsor.
- It is expected that most discrepant findings amongst the DSMB members will be resolved via discussion. In case of continuing discrepant findings amongst the DSMB members, majority will rule.
- DSMB activities and findings will be recorded by the DSMB in the meeting minutes and logged in the trial master file.
- The DSMB will meet periodically during the course of the study to review events, no less frequently than 2 times per year during the study.

22.2. Appendix B. Results of Safety Literature Review on ARC or Equivalent Therapies

First Author	Title	Journal/Yr.	Type of Article/Study	Condition/Patient Population	N	Location of Stimulation	Duration of Stimulation	Stimulation Parameters	Similarity to ARC	In Clinic/Home Use?	AEs
Gross, Tobias	Transcutaneous Electrical Nerve Stimulation for Treating Neurogenic Lower Urinary Tract Dysfunction: A Systematic Review	Eur Urol (2016)	Review	Neurogenic lower urinary tract dysfunction	450	Several (sacral, clitoral, penile, vaginal, rectal)	15 min/d-180 mins/d for 1-712 days	f: 5-75 Hz PW: 200-1500 ms Amp: Varied; 15-20 mA or up to max tolerable level	Location: Dissimilar Amplitude: ~16.7-25% of ARC	Both	No SAEs
Sivaramakrishnan, Anjali	Comparison of transcutaneous electrical nerve stimulation (TENS) and functional electrical stimulation (FES) for spasticity in spinal cord injury - A pilot randomized cross-over trial	J Spinal Cord Med (2018)	Double blind RCT	SCI (above L1) w spasticity	10	Muscles (quadriceps, adductor, plantar flexors)	30 mins	f: 100 Hz PW: 300 ms Amp: No greater than 20 mA	Location: Dissimilar Amplitude: ~16.7-25% of ARC	Both	Not reported
Mangold, S	Transcutaneous functional electrical stimulation for grasping in subjects with cervical spinal cord injury	Spinal Cord (2005)	Case series	Grasp function in C4/5-C7 SCI	11	Extrinsic finger extensors, extrinsic finger flexors, the thumb flexor	Several weeks to 2 years	f: 25-40 Hz PW: 0-250 ms Amp: Increased until muscle contraction was observed	Location: Dissimilar Amplitude: Cannot be determined	Both	Not reported
Lin, Mu-Lien	Two Transcutaneous Stimulation Techniques in Shoulder Pain: Transcutaneous Pulsed Radiofrequency (TPRF) Versus Transcutaneous Electrical Nerve Stimulation (TENS): A Comparative Pilot Study	Pain Res Mgmt (2019)	Double blind RCT	Shoulder Pain	50	Shoulder	15 mins/day for 3 mos.	f: 150 Hz PW: 700 ms Amp: 5.4 +/- 2.9 mA00 V (assuming a 300 ohms impedance, max current ~33.3 mA)	Location: Dissimilar Amplitude: ~27.8% of ARC	In Clinic	No AEs
Pahwa, Rajesh	An Acute Randomized Controlled Trial of Noninvasive Peripheral Nerve Stimulation in Essential Tremor	Neuromod (2019)	RCT	Tremor severity in Essential Tremor	77	Median nerve	40 mins	f: 150 Hz PW: 300 ms Amp: 100 V (assuming a 300 ohms impedance, max current ~33.3 mA)	Location: Dissimilar Amplitude: ~27.8% of ARC	In Clinic	No SAEs; 3% reported mild AEs

Kreuzer, Peter M	Feasibility, Safety and Efficacy of Transcutaneous Vagus Nerve Stimulation in Chronic Tinnitus: An Open Pilot Study	Brain Stim (2014)	Single arm, open label	Tinnitus	50	Auricular vagus nerve	24 weeks	f: Hz<br>PW: ms<br>Amp:	Location: Dissimilar<br>Amplitude:		Twitching and pressure at electrode placement site; one hospitalization-palpations and development of a left bundle branch block; both unrelated to the intervention
Rimmer, Craig J	Short-term Outcomes of a Randomized Pilot Trial of 2 Treatment Regimens of Transcutaneous Tibial Nerve Stimulation for Fecal Incontinence	Diseases of the Colon and Rectum (2015)	RCT	Fecal incontinence	22	Tibial nerve	1 or 4 hours/day, 2X week, 6 weeks	f: Hz<br>PW: ms<br>Amp:	Location: Dissimilar<br>Amplitude:		No AEs
Straube, Andreas	Treatment of Chronic Migraine with Transcutaneous Stimulation of the Auricular Branch of the Vagal Nerve (Auricular t-VNS): A Randomized, Monocentric Clinical Trial	J Headache Pain (2015)	Double blind RCT	Chronic migraine	46	Auricular vagus nerve	4 h/day, 3 months	f: 1 or 25 Hz<br>PW: 250 ms<br>Amp: Not specified (increased until tingling sensation felt)	Location: Dissimilar<br>Amplitude: Cannot be determined	Home	No study related SAEs; 115 tx related AEs. Most frequent treatment-related AE: local problems at the stimulation site, such as mild or moderate pain, paresthesia, or pruritus during or after stimulation, and erythema, ulcer, or scab (31 events in 10 patients in the 1 Hz group, 70 events in 17 patients in the 25 Hz group, p = 0.14).
Norrbrink, Cecilia	Transcutaneous electrical nerve stimulation for treatment of spinal cord injury neuropathic pain	J Rehab Res & Dev (2009)	Randomized, crossover	Pain in SCI	24	Paraspinal stimulation	30-40 mins/d, 3X/day for 2 weeks	f: 80 Hz or 2 Hz burst (8 pulses at 80 Hz/burst)<br>PW: 180 ms<br>Amp: Highest intensity that did not cause pain	Location: Similar<br>Amplitude: Cannot be determined	Home	Discomfort or increased pain during treatment (n=3), local muscle spasms (n=1)

Inanici, Fatma	Transcutaneous Electrical Spinal Stimulation Promotes Long-Term Recovery of Upper Extremity Function in Chronic Tetraplegia	IEEE Trans Neural Sys and Rehab Engg (2018)	Case report	Function restoration in cervical SCI	1	Paraspinal stimulation	25-120 mins/session, 5 weeks	f: 30 Hz and a carrier freq of 10 kHz PW: 1000 ms Amp: 80-120 mA	Location: Similar Amplitude: Similar	In Clinic	No AEs or SAEs
Inanici, Fatma	Non-invasive spinal cord stimulation restores hand function after paralysis	In Review (NA)	Case series	Function restoration in cervical SCI	6	Paraspinal stimulation	up to 120 mins/session, up to 3X/week, 8 weeks	f: 30 Hz and a carrier freq of 10 kHz PW: 1000 ms Amp: Highest amplitude w/o discomfort	Location: Similar Amplitude: Similar	In Clinic	No AEs
Sayenko, Dimitry	Self-Assisted Standing Enabled by Non-Invasive Spinal Stimulation after Spinal Cord Injury	J Neurotrauma (2019)	Double-blind, within-subject crossover, sham-controlled	Standing function in SCI	15	Paraspinal stimulation	120 mins/session, 3X/week, 4 weeks	f: 0.2 - 30 Hz and a carrier freq of 10 kHz PW: 1000 ms Amp: Up to 150 mA	Location: Similar Amplitude: Similar	In Clinic	1 non-study related AE
Gad, Parag	Non-Invasive Activation of Cervical Spinal Networks after Severe Paralysis	J Neurotrauma (2018)	Case series	Voluntary motor control in cervical SCI	8	Paraspinal stimulation	60-120 mins/session, 4 weeks	f: 30 Hz and a carrier freq of 10 kHz PW: 1000 ms Amp: 10 - 250 mA	Location: Similar Amplitude: Similar	In Clinic	No AEs

22.3 Appendix C: Publication Policy

Introduction

This document outlines the publication policy and authorship criteria for ONWARD Medical, Inc.'s Up-LIFT pivotal study. Details regarding publication agreed upon by the research site and/or the principal investigator (PI) in the clinical trial agreement (CTA), if differing from this policy, will be reconciled as needed to preserve principles of authorship outlined herein.

Authorship

Authors must meet the following criteria laid out by the International Committee of Medical Journal Editors (ICMJE) (available at http://www.icmje.org).

- Substantial contributions to the conception or design of the work; or the acquisition, analysis, or interpretation of data for the work; AND
- Drafting the work or revising it critically for important intellectual content; AND
- Final approval of the version to be published; AND
- Agreement to be accountable for all aspects of the work in ensuring that questions related to the accuracy or integrity of any part of the work are appropriately investigated and resolved.

Non-contributing authors that do not meet all of the above-mentioned criteria can be acknowledged individually or as a group as determined by the study PIs or co-PIs along with the sponsor.

Order of authors

- Authorship order shall be determined using the six criteria listed in the sample scoring matrix shown below.

Author	Conception and Design	Data Acquisition	Analysis	Interpretation	Drafting	Revision	Enrolled	Sum
Author1	1	5	1	1	2	1	31	11
Author2	1	5	1	1	1	1	0	10
Author3	3	0	2	2	2	1	30	10
	Significant=3<br>Minimal=1<br>None=0	>15%=5<br>≥10%=3<br><10%=2	Primary=2<br>Substantial=1	Primary=2<br>Substantial=1	Primary=2<br>Minimal=1	1	Tie Break	

- Authorship will be assigned in a decreasing order of the total sum of these 6 criteria.
- Exception to the rule: The sponsor-designated study PI or the co-PIs of the study shall serve as the first and/or the senior author as agreed upon by the PIs and/or the sponsor.
- In case of a tiebreak, the site PI that enrolled the most number of subjects will be given priority.

- Either the study PI or an author from ONWARD Medical, Inc. may serve as the corresponding author.

Conflict of interest

All authors must disclose all of their active conflict(s) of interest at the time of manuscript submission as accurately as possible.

Publication Review and Timeline

- Allowable publication cadence
    - During the study – Manuscripts and abstracts on methodology on behalf of all investigators, without endpoint data.
    - After the study – Principal manuscript on endpoint data for all sites. Once the former is accepted for publication, secondary manuscripts on subgroups, technology, best practices, etc. can be submitted for review.
    - After publication of the main manuscript – Other topics including any site-specific data and other areas of related interest.
- Publishing site-specific data
    - After the publication of the main study manuscript, Institution and PI shall have the right to publish their site-specific data as negotiated in their respective clinical trial agreement (CTA).
    - PI, other Institution investigators, employees and agents must submit a manuscript or abstract to ONWARD at least sixty (60) days prior to any submission for publication or presentation to provide ONWARD an opportunity for review.

23. TABLES

Table 1. List of feasibility studies using the LIFT device 26
Table 2. Demographics of upper extremity SCI subjects enrolled in the LIFT feasibility studies 28
Table 3. Listing of all AEs and SAEs reported in the feasibility studies using LIFT device 31
Table 4. Parameters of the LIFT stimulator 36
Table 5. MID definition based on pilot study data for primary endpoint outcomes 39
Table 6. List of outcomes to assess improvements in upper extremity strength and function at primary endpoint 39
Table 7. Schedule of events for the study 47
Table 8. A summary of anticipated risks related to the use of the device 59
Table 9. A summary of the proposed mitigation(s) for each risk identified in Table 8. 60
Table 10. List of potential primary serious adverse events 64
Table 11. List of some known AEs and their definitions 67

24. FIGURES

Figure 1. Adapted from Kalsi-Ryan and Verrier, Physiotherapy Canada, 2011 21
Figure 2. The LIFT System components (top left) when used for non-invasive electrical spinal stimulation at the cervical level (top right). A close-up of the tablet display of the programmer (bottom). 35
Figure 3. Study flow diagram listing all clinical assessment visits 43
Figure 4. Potential electrode locations for stimulation and a sample stimulation waveform (bottom inset) using round hydrogel electrodes (top inset). Figure adapted from Inanici et al. (Inanici, et al., 2018) 50
