# Supplementary material for: Non-invasive spinal cord electrical stimulation for arm and hand function in chronic tetraplegia: a safety and efficacy trial
Source: Nat Med. 2024 May 20;30(5):1276–83. doi: 10.1038/s41591-024-02940-9 (PMC11108781; doi:10.1038/s41591-024-02940-9)
Supplement: Supplementary file 6 — Pre-registered statistical analysis plan. [file 41591_2024_2940_MOESM6_ESM.pdf]

Statistical Analysis Plan

ONWARD Medical, Inc.

Up-LIFT STUDY

Table of Contents

1 Introduction 4
2 Study Objectives 4
  2.1 Safety Objective 4
  2.2 Effectiveness Objective 4
  2.3 Study Endpoints 4
3 Study Design 6
4 Sample Size Determination 6
5 Statistical Analyses 6
  5.1 General Considerations 6
    5.1.1 Descriptive Statistics 7
    5.1.2 Study Day 7
    5.1.3 Visit Windows 7
    5.1.4 Statistical Significance 7
  5.2 Analysis Populations 7
  5.3 Handling of Missing Data 8
  5.4 Subject Disposition 8
  5.5 Demographics and Baseline Characteristics 8
  5.6 Analysis of Study Endpoints 8
    5.6.1 Primary Safety Endpoint 8
    5.6.2 Primary Effectiveness Endpoint 8
  5.7 Secondary Endpoints 11
    5.7.1 Secondary Safety Endpoint 11
    5.7.2 Secondary Effectiveness Endpoints 11
  5.8 Sensitivity Analyses 12
  5.9 Poolability Analyses 12
  5.10 Safety Analyses 12
  5.11 Subgroup Analyses 13
  5.12 Interim Analyses 13
  5.13 Protocol Deviations 13
  5.14 Other Analyses 13
6 Exploratory Analyses 14
7 Changes from Planned Analyses 15
8 Subject Listings 15
9 Appendix 16
  9.1 Background 16
  9.2 Post-Injury Timeline: Recovery of Neurological Status, Function and Plateau 16
  9.3 Treatment Options for Individuals with SCI: Occupational Therapy and Functional Task Practice 17

9.4 Neurostimulation and ARC Therapy – Feasibility Data 18

9.5 Rationale for the Choice of Primary Endpoint 18

9.6 Rationale for the Choice of Secondary and Observational Endpoints 19

9.7 Study Design Considerations 20

10 References 21

1 Introduction

This statistical analysis plan (SAP) describes the planned statistical methods to be used during the reporting and analysis of data collected under the Clinical Investigation Protocol the Up-LIFT Study. This SAP should be read in conjunction with the study clinical investigation plan (CIP). A review of the background of traumatic spinal cord injury (SCI), post-injury recovery and treatment options is provided in Appendix sections 8.1-8.4. This version of the SAP was initially developed with respect to the Clinical Investigation Protocol Version C and subsequently amended to correspond with Version D. Any revisions to the protocol or CRFs that impact the planned analyses may require updates to the SAP.

Applicable Documents:

Document Number, Version	***Document Title***
UpLIFT Protocol (CIP)	Clinical assessment of upper extremity performance in individuals with spinal cord injury using the LIFT System to deliver non-invasive electrical spinal stimulation (ARC Therapy)

2 Study Objectives

2.1 Safety Objective

To provide confirmatory evidence that use of the LIFT System, inclusive of all components and accessories, is safe.

2.2 Effectiveness Objective

To provide confirmatory evidence that use of the LIFT System provides an effective treatment for the restoration or improvement in upper extremity strength and function.

2.3 Study Endpoints

The choice of study endpoints was dictated by several factors discussed in the Appendix sections 8.5 and 8.6.

Primary:

Safety: Observational data regarding the incidence of serious adverse events (SAEs) related to the use of the study device and treatment procedures will be reported.

Effectiveness: The primary effectiveness outcome measure will test the hypothesis that a majority of the subjects will experience clinically significant improvement in selected strength and functional performance metrics after treatment with ARC therapy administered by the LIFT System and functional task practice (FTP). A subject will be considered a treatment responder if she/he reports clinically relevant improvements in at least one outcome each of the Strength and Function domains as follows:

Strength		Function
ISNCSCI-UEMS		GRASSP-Prehension Performance
GRASSP-Strength	and	CUE-T Total
Pinch force (sum of lateral and tip)		
Grasp force		

Secondary:

Safety: All adverse events (AEs) and SAEs in the study will be reported.

Effectiveness: To capture meaningful improvements in established outcomes assessing upper extremity function, the following hierarchical testing will be carried out. These endpoints will be tested in descending order of importance through hierarchical testing as described below.

- Superiority of combined FTP and ARC Therapy with LIFT vs. FTP alone as described by statistically significant difference in responder rates (comparison of change from enrollment baseline to end of FTP with the change from enrollment baseline to end of combined FTP and ARC Therapy with LIFT)
- Quantitative comparison of individual performance metrics to establish superiority of FTP and ARC Therapy with LIFT compared to FTP alone:
  - Pinch force
  - GRASSP-Prehension
  - GRASSP-Strength
  - ISNCSCI-UEMS
  - ISNSCI-Total sensory score
  - EQ-5D-5L
  - SCIM
  - WHOQOL-BREF

Observational:

Descriptive statistics on additional domains in the primary and secondary endpoint outcomes will be reported. The following additional assessments impacting patient quality of life and long-term consequences of SCI such as outcomes related to pain, spasticity, quality of life, blood pressure and bladder/bowel/sexual function will be reported as descriptive statistics and clinically relevant changes, where possible.

- Numerical Rating Scale (NRS) for pain
- International Spinal Cord Injury Pain Data Set (ISCIPDS)
- Medical Outcomes Study (MOS) Sleep Scale
- Spinal Cord Independent Measure (SCIM III)
- Penn Spasm Frequency Scale (PSFS)
- EuroQol 5 Dimension 5 Level Questionnaire (EQ-5D-5L)

- World Health Organization Quality of Life (WHOQOL-BREF)
- International Standards to document remaining Autonomic Function after Spinal Cord Injury (ISAFSCI)
- Patient Health Questionnaire (PHQ-9)
- Global Impression of Change (Clinician and Patient)

Version D of the clinical investigational plan was amended to include an optional follow-up assessment at the conclusion of the study. This assessment is outside the planned study endpoints and analyses but will provide useful data to understand the therapy effect. The additional data is descriptive only and no formal statistical hypothesis test will be performed, nor any claims made, as this test is optional. The follow-up assessment is informational only and intended as hypothesis generating data.

The follow-up assessment is intended to evaluate the immediate neuroprosthetic effect of active stimulation on strength and motor function; a relevant set of assessments (CUE-T, GRASSP Strength, GRASSP Prehension, Pinch force, Grasp force, and Global Impression of Change questionnaire) will be performed with stimulation both off and then on. This optional assessment will be scheduled from 1-30 days after the completion of the 4 month evaluation and will not impact the collection, tabulation, or reporting of other endpoints in the study.

3 Study Design

Up-LIFT study is a prospective, single-arm study designed to evaluate the safety and effectiveness of non-invasive electrical spinal stimulation (ARC Therapy) administered by the LIFT System to treat upper extremity functional deficits in people with chronic tetraplegia. Alternate study designs were considered and are discussed in Appendix Section 8.7. However, given the condition has no viable treatment options and an ineffective standard of care, a single-arm, non-randomized study was chosen as a pragmatic option.

The primary endpoint will report device related safety and changes in established metrics of upper extremity function and strength after treatment with the study device.

4 Sample Size Determination

The primary effectiveness endpoint is a responder analysis with a responder defined as a subject who demonstrates improvement in at least 1 outcome in each of the strength and function domains. Assuming a minimum power of 80%, a two-sided type I error of 10% (one-sided 5%), a responder rate of 67%, a 50% performance goal and 25% drop out rate, a sample size of 65 subjects is required to be enrolled in the study across a maximum of fifteen sites. Fifty-two of these subjects are intended to complete their four-month visit to ensure the primary endpoint is statistically powered. Any single site may enroll up to a maximum of 25% of the total sample size.

5 Statistical Analyses

5.1 General Considerations

Except where otherwise specified, the following general principles apply to the planned statistical analyses. All statistical analysis will be conducted using SAS version 9.4 or later (SAS Institute Inc., Cary, NC) or other widely accepted statistical or graphical software as required.

5.1.1 Descriptive Statistics

Continuous data will be summarized with mean, standard deviation, median, minimum, maximum, and number of evaluable observations. Categorical variables will be summarized with frequency counts and percentages. Confidence intervals may be presented, where appropriate, using the t-distribution for continuous data and Clopper-Pearson Exact method for categorical variables.

5.1.2 Study Day

Study Day 0 is the date the subject begins conventional functional task practice (FTP) or FTP and ARC Therapy. Day in study (for each phase) will be calculated relative to Day 0 as follows:

Study Day = Assessment Date – Day 0

For each subject, duration in study will be based on last study contact date which is the latest date of all follow-up visits, assessments, adverse event onset or resolution, and study exit including date of death.

Duration variables will be calculated as follows:

Duration Days = Start Date – End Date

5.1.3 Visit Windows

Unless otherwise specified, visit based assessments will be analyzed for each analysis time point according to the nominal visit entered in the Case Report Form (CRF) regardless of if it is out of window.

5.1.4 Statistical Significance

Unless otherwise specified, hypothesis testing will be performed at the one-sided 0.05 significance level. P-values will be rounded to three decimal places. If a p-value is less than 0.001 it will be reported as "<0.001". If a p-value is greater than 0.999, it will be reported as ">0.999".

5.2 Analysis Populations

The following analysis populations are defined for analysis:

1. **Intent-to-Treat (ITT) Analysis Population**: Any subject consented and enrolled in the study.
2. **Safety Analysis Population**: Any ITT subject that is exposed to the study treatment. This is a subset of the ITT population.
3. **Modified Intent-to-Treat (mITT) Analysis Population**: Subjects who meet ALL of the following criteria are included in the mITT analysis population.
    - Undergo at least 24 sessions of FTP during the wash-in period
    - Undergo at least 24 sessions of FTP + ARC therapy with LIFT during the treatment period
4. **Per-Protocol Population (PPP):** Subjects included in the study that met all inclusion criteria and none of the exclusion criteria, were free from major deviations and in which treatment was attempted with the LIFT System.

Safety endpoints are reported in the Safety Analysis Population while the study effectiveness endpoints are assessed in the mITT population. Additional sensitivity analyses of effectiveness measures will be reported in the Safety and PP populations.

Justification for the use of mITT to report effectiveness: The study requires that a subject visit the clinic over 50 times during the course of a 4-month period. During most of these visits, subjects will undergo an intensive FTP regimen. It is reasonable to assume that mobility-impaired tetraplegic subjects may find the number of study visits burdensome, due to issues associated with transportation and caregiver availability, amongst many others. This is likely to result in dropouts during the course of the study. Moreover, a minimum number of exposures to both interventions is required to make a fair comparison of the treatment types. Hence, the primary effectiveness endpoint of the study is reported in a population adequately exposed to both therapies. Additional assessments will report dropout rate.

5.3 Handling of Missing Data

All attempts will be made to limit the amount of missing data. Unless otherwise specified, no attempt will be made to impute missing data. If a data point is missing, that data point will not contribute to that portion of the analysis. The number of evaluable observations will be reported in analysis so that extent of missing data can be assessed.

5.4 Subject Disposition

The number of subjects in each analysis population will be presented along with reason for any exclusions. Subject accountability will be summarized by visit. The number of subjects who are enrolled, eligible for follow-up, and number completing FTP and FTP+ARC Therapy will be summarized. Overall therapy compliance will be calculated for each subject and summarized as the number and percent of completed therapy sessions out of the number expected. In addition, the number of subjects who complete the study or exit early will be summarized by reason.

5.5 Demographics and Baseline Characteristics

Descriptive statistics will be presented for all clinically-relevant baseline demographic, medical history, and clinical characteristic variables.

5.6 Analysis of Study Endpoints

5.6.1 Primary Safety Endpoint

Observational data regarding the incidence of serious adverse events (SAEs) related to the use of the study device and treatment procedures will be reported.

No formal statistical hypothesis will be tested. The endpoint will be summarized in the safety analysis population. The number of related SAEs as well as the number and percent of subjects with one or more related SAE will be reported. The 95% exact confidence interval will be reported.

5.6.2 Primary Effectiveness Endpoint

The primary effectiveness outcome measure will test the hypothesis that a majority of the subjects will experience clinically significant improvement in selected strength and functional performance metrics after treatment with ARC Therapy administered by the LIFT System and FTP. A subject will be considered a treatment responder if she/he reports clinically relevant improvements (defined in Section 5.6.2.1) in at least one outcome each of the Strength and Function domains as follows:

Strength		Function
ISNCSCI-UEMS		GRASSP-Prehension Performance
GRASSP-Strength	and	CUE-T Total
Pinch force (sum of lateral and tip)		
Grasp force		

For primary efficacy components within the composite that are measured bilaterally, the responder assessment against the MID for change from baseline will be based on the sum of the two measurements. The primary effectiveness endpoint will be assessed with the following hypothesis:

H_0: $\Pi \leq 0.50$

H_a: $\Pi > 0.50$

where Π is the proportion of subjects meeting the responder criteria. The hypothesis will be evaluated using a one-sided statistical test for exact binomial test. The objective will be met if the p-value is < 0.05.

The endpoint will be evaluated using the mITT population which will serve as the effectiveness analysis set.

The numerator will include the number of subjects who meet the responder criteria and the denominator will include all subjects evaluable for the primary effectiveness endpoint. The number and percent of responders to each of the components of the composite will also be reported.

Each component of the primary effectiveness composite will be summarized separately. This will include summary statistics at each visit as well as summaries of change from enrollment baseline. The summaries of change for each parameter will include 90% confidence limits. To illustrate the magnitude of change in each component, waterfall plots will be reported showing each subject's response in ascending order with a reference line at the minimal important difference as defined in Section 5.6.2.1.

5.6.2.1 Determination of Clinically Meaningful Improvement Using Minimal Important Difference (MID)

In the absence of relevant prior data on minimum detectable difference (MDD) or minimal clinically important difference (MCID) in this specific, chronic SCI population, an appropriate alternative is to use the effect size as described by Cohen [1] as follows:

Cohen's effect size, $d = \frac{m1 - m2}{\sqrt{\frac{(\sigma 1^2 + \sigma 2^2)}{2}}}$

where,

m1, m2 = Means of two independent samples

σ1, σ2= Standard deviations of the respective populations

Cohen's effect size is simply a measure of difference in means between the test and the control populations. It measures the degree to which a phenomenon exists in the population or is induced by the treatment. It can also quantify the degree to which the null hypothesis is false. The larger the effect size, the greater the certainty with which the null hypothesis can be rejected and more clinically relevant the outcome. Small, medium and large effect sizes were defined as 0.2, 0.5 and 0.8, respectively.

In a comprehensive review, Wu et al. discussed the limitations of not being able to use MDD/MCID in the chronic SCI population and instead suggest that Cohen's effect size (d) is a better statistic to use [2]. Using this method, one can calculate the minimal important difference (MID) for each metric within the primary endpoint resulting from exposure to ARC Therapy:

MID = $d*\sqrt{(\sigma1^2 + \sigma2^2)/2}$

This definition was applied for a small effect size (d=0.2) in a small but representative sample of feasibility study data collected in up to 13 subjects from across 3 sites to define a responder for the various primary outcome measures listed in the table below. To be deemed a responder, a subject has to meet or exceed the values listed below for the respective outcomes.

S. No.	Outcome	MID
**1**	ISNCSCI-UEMS	2-point change
**2**	GRASSP-Strength[1]	4-point change
**3**	GRASSP-Prehension Performance[1]	2-pt change
**4**	Pinch force (sum of lateral and tip)[1]	≥2.4 N
**5**	Grasp force[1]	≥6 N
**6**	CUE-T Total	4-point change

[1]Left and right measurements are summed

It is understood that calculating MID using this approach has two major limitations:

1. Treatments offered to subjects (both FTP and ARC Therapy) differed somewhat with different outcome data collected at each site.
2. The pilot data had a small sample size with large variations. Thus, it is likely that the MID values may be artificially inflated.

The former issue will be addressed in the consistent implementation of a uniform pivotal study protocol across all sites. The latter is addressed through a pre-defined sensitivity analysis as discussed in Section 5.8.

5.7 Secondary Endpoints

5.7.1 Secondary Safety Endpoint

All adverse events (AEs) and SAEs in the study will be reported.

5.7.2 Secondary Effectiveness Endpoints

The following secondary effectiveness endpoints will be tested in descending order of importance through hierarchical testing. In order to maintain the one-sided overall Type I error of 5% the secondary effectiveness endpoints will be tested only if the primary objective is met and in the order listed below. Testing will stop if an endpoint is not met with no subsequent hypotheses tested. Each endpoint will be tested using a one-sided test with Type I error of 5%.

- Superiority of combined FTP and ARC Therapy with LIFT vs. FTP alone as described by statistically significant difference in responder rates (using the same definition as the primary effectiveness endpoint; comparison of change from enrollment baseline to end of FTP with the change from enrollment baseline to end of combined FTP and ARC Therapy with LIFT).

  The hypotheses to be tested are:

  H_0: $\Pi_{FTP+ARC} \leq \Pi_{FTP}$
  H_A: $\Pi_{FTP+ARC} > \Pi_{FTP}$

  where $\Pi_{FTP+ARC}$ is the responder rate after FTP and ARC Therapy and Π_{FTP} is the responder rate after FTP alone. The hypothesis will be tested using a McNemar's test. If the p-value is less than 0.05 and the disagreement is in favor of FTP and ARC Therapy the null hypothesis will be rejected in favor of the alternative.

- Quantitative comparison of individual performance metrics to establish superiority of FTP and ARC Therapy with LIFT compared to FTP alone:

  - Pinch force (sum of lateral and tip)
  - GRASSP-Prehension Performance
  - GRASSP-Strength
  - ISNCSCI-UEMS
  - ISNSCI-Total sensory score
  - EQ-5D-5L
  - SCIM
  - WHOQOL-BREF

  For endpoints that are measured bilaterally, the endpoint will be the sum of the two measurements. Each of these continuous outcomes will be tested under the following hypotheses:

  H_0: $\mu_{FTP+ARC} \leq \mu_{FTP}$
  H_A: $\mu_{FTP+ARC} > \mu_{FTP}$

where $\mu_{FTP+ARC}$ is the mean improvement from baseline after FTP and ARC Therapy and μ_{FTP} is the mean improvement from baseline after FTP alone. The hypotheses will be tested using one-sided paired t-tests if normality assumptions are not violated, or Wilcoxon signed rank tests if data are non-normal. The secondary endpoints will be summarized by visit and include change from enrollment baseline and change from the end of FTP alone. The summaries for change will include 90% confidence limits.

5.8 Sensitivity Analyses

Sensitivity analyses for the primary effectiveness endpoint will be performed as follows:

- Analysis including all subjects enrolled in the study (ITT population). This will include a worst-case analysis where all subjects with missing endpoint data are imputed as failures.
- Analysis including all subjects exposed to the ARC therapy (safety population)
- Analysis restricted to subjects who meet all of the inclusion criteria and none of the exclusion criteria, were free from major deviations, who completed all required therapy sessions and in which treatment was attempted with the LIFT System (PP population)
- The analysis to determine MIDs in the primary effectiveness composite will be repeated with final data. The calculations outlined in Section 5.6.2.1 will be repeated using the study data deriving new, *post-hoc* MIDs. The primary effectiveness analysis will then be repeated based on the new MIDs. The purpose will be to validate the levels chosen for the primary effectiveness endpoint and to provide a supportive, *post-hoc*, sensitivity re-analysis of the primary endpoint using the study data derived MIDs.
- An additional analysis of the primary effectiveness composite outcomes will use selected outcome measures (e.g., PGIC, SCIM) as potential anchors for a *post-hoc* determination of MCID change in the primary effectiveness outcomes. Correlations between the anchor outcomes and the primary efficacy composite components will be calculated. This will provide an additional supportive, *post-hoc*, sensitivity re-analysis of the primary endpoint using study data derived MCIDs.

5.9 Poolability Analyses

All investigational sites will follow the requirements of a common protocol and standardized data collection procedures and forms. The primary endpoint will be presented separately for each site using descriptive statistics. Poolability of the primary endpoint across investigational sites will be evaluated using a chi-square test. In the event any sites have <5 subjects enrolled, the low enrolling sites will be combined into a single pseudo-site for the purposes of this analysis. If the p-value is <0.15 additional exploratory analyses will be performed to understand any variations in outcomes by site.

5.10 Safety Analyses

Adverse events (AE) will be reported for the Safety Analysis population. AEs will be tabulated with the number of events and subjects with event for each event type and overall. Rates will be reported as the number of subjects who experience at least one event during the analysis interval out of the total number of subjects with follow-up to the beginning of the analysis interval. Serious adverse events will also be tabulated.

All AEs and SAEs will also be summarized by relatedness as described above. Adverse events leading to death or study discontinuation will be provided in listing format.

All device deficiencies will be reported in listing format.

5.11 Subgroup Analyses

Subgroup analyses of the primary and secondary effectiveness endpoints will be performed for the following subgroups:

- Sex
- Age at enrollment (stratified at the median age)
- Age at the time of injury (stratified at the median age)
- Length of time from injury (stratified at the median time)
- Neurological Level of injury (some combination of levels may be conducted to create subgroups of reasonable size for analysis purposes)
- ASIA Impairment Scale (B, C, D)
- Starting UEMS (stratified at median)
- Starting GRASSP Strength (stratified at median)
- Starting GRASSP Prehension (Ability and Performance, each stratified at median)
- Starting CUE-T scores (stratified at median)
- Starting pinch/grasp forces (stratified at median)
- Starting Penn Spasm Frequency (stratified at median)

Subgroup analyses will be conducted in the mITT analysis population. The responder rate for binary endpoints or summary statistics for change from baseline for continuous endpoints will be presented for each level of the subgroup with associated 90% confidence limits and a p-value to test for differences in outcome across subgroup levels (Chi-square or Fisher's exact as appropriate for binomial outcomes and t-test or Wilcoxon rank sum for continuous).

5.12Interim Analyses

No interim analyses are planned.

5.13Protocol Deviations

Deviations from the procedures outlined in the CIP will be reported by investigational sites on the CRF. Protocol deviations will be summarized for all deviations and by type with event counts and number of subjects with at least one deviation.

5.14 Other Analyses

Descriptive statistics on additional domains in the primary and secondary endpoint outcomes will be reported. Additional scales and separate summaries by dominant and non-dominant side will be reported for the secondary effectiveness outcome measures. The following additional assessments impacting patient quality of life and long-term consequences of SCI such as outcomes related to pain, spasticity, quality of life, blood pressure and bladder/bowel/sexual function will be reported as descriptive statistics by visit, including change from baseline at 2 and 4 months (FTP alone and FTP+ARC).

- o Pinch force (summarized for lateral and tip separately, and by dominant/non-dominant side)

- ISNCSCI (all motor and sensory total and subscores outlined in Section 5.15 summarized separately and by dominant/non-dominant side)
- GRASSP – Prehension (both ability and performance, summarized overall by dominant/non-dominant side)
- GRASSP – Strength (by dominant/non-dominant side)
- GRASSP – Sensory (by dominant/non-dominant side)
- CUE-T and all of the calculated subscores outlined in Section 5.15 (by dominant/non-dominant side)
- Numerical Rating Scale (NRS) for pain (both 24 hour and 4 week will be summarized)
- International Spinal Cord Injury Pain Data Set (ISCIPDS)
  The Pain Indicator will be summarized categorically as 0 (none reported), 1, 2, 3, 4, or 5 or more pain problems reported. Continuous summary statistics will be reported for Pain Interference for the three scales measures (day to day activities, overall mood, sleep).
- Medical Outcomes Study (MOS) Sleep Scale
- Spinal Cord Independent Measure (SCIM III)
  The total score as well as the self-care and upper extremity subscale score will be summarized.
- Penn Spasm Frequency Scale (PSFS)
- EuroQol 5 Dimension 5 Level Questionnaire (EQ-5D-5L)
- World Health Organization Quality of Life (WHOQOL-BREF)
- International Standards to document remaining Autonomic Function after Spinal Cord Injury (ISAFSCI)
- Patient Health Questionnaire (PHQ-9)
- Global Impression of Change (Clinician and Patient)

5.15 Exploratory Analyses

Additional exploratory analyses will be conducted on the study data. Statistical tests for significant change from either baseline or end of FTP alone to end of ARC+FTP will be conducted and are intended for exploratory purposes only. Tests for statistically significant change will be by paired t-test or Wilcoxon sign rank if data are found to be non-normal. No formal statistical hypothesis tests will be conducted and no labeling claims will be made based on these analyses.

For the analyses listed in Section 5.14, assessments for statistical significance of changes from baseline and change from the end of FTP alone will be conducted.

Additional metrics will be calculated from selected study instruments. The calculated outcomes will be summarized with descriptive statistics, change from baseline and change from the end of FTP alone, and statistical tests for significant change.

- From ISNCSCI:
  - UESS (Upper Extremity Sensory Score)
    - Subscores for dominant and non-dominant side as well
    - Calculated as the sum of C2-T1 LT and PP scores on either both sides (for combined UESS) or unilaterally for the dominant and non-dominant sides
  - UE-LT (Upper Extremity Light Touch Score)
    - Subscores for dominant and non-dominant side as well
    - Calculated as the sum of C2-T1 LT scores on either both sides (for combined UE-LT), or unilaterally for the dominant and non-dominant sides

  - UE-PP (Upper Extremity Pin Prick Score)
    - Subscores for dominant and non-dominant side as well
    - Calculated as the sum of C2-T1 PP scores on either both sides (for combined UE-PP), or unilaterally for the dominant and non-dominant sides
  - UEMS Subscores
    - As per above outcomes, please calculate subscores for the dominant and non-dominant sides, in addition to the already reported UEMS for combined sides
    - Calculated as the sum of C5-T1 motor scores unilaterally for the dominant and non-dominant sides
- From CUE-T
  - Total, right hand, left hand
  - Left Arm (/24): calculated as the difference between Unilateral Left – Left Hand
  - Right Arm (/24): calculated as the difference between Unilateral Right – Right Hand
- From SCIM
  - Upper Extremity items (*Self-Care*): calculated as the sum of the individual scores for Bathing A + Dressing A + Feeding + Grooming

Mixed repeated measures modeling will be used to analyze the following outcomes. The models will include covariates for visit and FTP vs ARC+FTP to assess for differences between the FTP period and the ARC+FTP period. Estimates will be provided by visit. Models will be done separately for the outcome variable by visit and change in the outcome variable from baseline by visit. Models of change from baseline will include the baseline value as a covariate.

- Blood pressure (systolic and diastolic)
- Heart rate
- Oximetry
- Box and block

6 Changes from Planned Analyses

Any changes to planned statistical analyses determined necessary prior to performing the analyses will be documented in an amended Statistical Analysis Plan and approved prior to the analysis when possible. Any other deviations or changes from the planned analyses deemed necessary due to violation of critical underlying statistical assumptions, data characteristics, or missing data will be clearly described in the clinical study report with justification and rationale.

7 Subject Listings

Subject listings will be provided for all adverse events and primary and secondary effectiveness outcomes, adverse events, and protocol deviations.

8 Appendix

8.1 Background

Of the 17,000 new cases every year, traumatic SCI results in either complete or incomplete tetraplegia in 58% of cases [3]. Etiology of the injury in nearly two-thirds of these cases involve vehicular accidents or falls. Tetraplegia results in loss of hand and arm function which may significantly limit independence with activities of daily living (ADLs) and result in a reduced quality of life. Recovery of this function is often the top priority for these individuals [4, 5, 6]. In persons with tetraplegia, hand muscle force generation is highly correlated with success or failure in the ability to perform common functional tasks [7]. Restoration of upper extremity function is cited as the highest priority for individuals with SCIs, five times greater than other functions like bladder, bowel, sexual or movement [4].

For individuals with chronic SCI, along with impaired sensorimotor issues, the secondary complications associated with immobility may present additional challenges. These include complications to respiratory, cardiovascular, urinary, and bowel systems. Additionally, individuals with SCI may experience spasticity, pain, pressure ulcers, osteoporosis and bone fractures leading to significant health complications and reduced quality of life [8].

8.2 Post-Injury Timeline: Recovery of Neurological Status, Function and Plateau

Post-injury, the most accurate predictor of neurologic recovery involves a thorough physical examination of the individual with acute SCI to determine the initial level of injury [9]. Those with complete tetraplegia, during the natural course of recovery will regain one motor level and the initial strength of the muscle is a significant predictor of achieving antigravity strength.

In incomplete SCI, there is a great deal of spontaneous recovery compared to complete SCI. More than half of the individuals with incomplete tetraplegia become ambulatory. In 1-year post-injury, 15-40% of American spinal injury Association impairment scale (AIS) B subjects improve to AIS C while another 40% are converted to AIS D. AIS C to D conversion varies from 60-80% [10].

In a multicenter study, >90% of subjects with incomplete injuries gained at least one additional motor level in the upper extremities compared with 70-85% of those with complete injuries [11]. Based on preservation of sensory function, there is good prognosis for motor strength recovery in this patient population [12].

In a majority of incomplete tetraplegics and paraplegics, there was an approximately 12- to 14-point increase in lower extremity motor scores during the first year and minimal improvement during the second year using the 50-point scale [12]. These changes taper anywhere between 12-18 months post-injury (Figure 1).

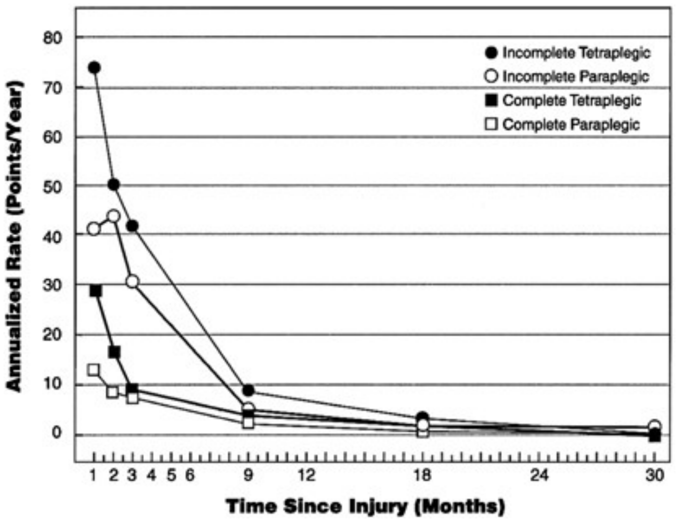

Figure 1. Annualized rate of gain in motor scores from the time of injury

In summary, functional improvements in individuals with chronic cervical SCI reaches an asymptotic value ("plateau") after approximately 18 months post-injury. Beyond that timepoint, only limited gains in motor recovery have been reported. Thus, any sizeable improvement in the functional status of a refractory SCI individual may translate into clinically meaningful change.

8.3 Treatment Options for Individuals with SCI: Occupational Therapy and Functional Task Practice

Treatment options for individuals with SCI include surgery to restore hand function, medications to reduce side effects of the injury including pain and spasticity, rehabilitation including electrical stimulation, and assistive technologies.

Individuals with tetraplegia undergo occupational therapy with the goal of improving upper extremity strength and function, trunk control, lower extremity strength and function, bladder catheterization to name a few. To improve upper extremity strength and function through movements, motor strategies that comprise common everyday activities called "functional task practice" (FTP) is offered as a primary therapeutic factor [13]. The practice involves repetitive tasks that require uni- or bi-manual operation including gross upper extremity movement, bilateral pinch, bilateral grasp, grasp, complex pinch and finger isolation. This results in a cortical reorganization in animals and individuals with neurological conditions like stroke that is use-dependent. This reorganization increases the area of the cortex involved in movement generation. The result is functional improvement that is reflected in increased scores on functional tests [14]. These findings support the use of FTP in incomplete SCI subjects as an effective control to compare any new therapies.

In appropriate candidates, surgical interventions to restore elbow extension, wrist extension, reconstruction of grasp function and stabilization of the thumb may help in restoring function with the goal to improve independence with ADLs [15]. Medications are often prescribed to manage the side effects of SCI like spasticity and pain. Antispastic agents like baclofen, benzodiazepines are prescribed to reduce hypertonicity and involuntary jerks of the muscles. Despite a heightened risk for side effects like dizziness, edema and somnolence, gabapentinoids like gabapentin and pregabalin are effective not only

in treating neuropathic pain but also secondary conditions associated with SCI like anxiety, depression, and sleep interference [16, 17].

Physical and occupational therapies focus on rehabilitation and restoration of function after SCI. Restorative therapies not involving surgery include use of exoskeletons or gait orthosis to enable or assist with mobility. Electrical stimulation is another restorative therapy gaining traction. The approach may be non-invasive or invasive and broadly, fall into 2 major groups: functional electrical stimulation (FES) or spinal cord stimulation (SCS) like ARC Therapy. The former targets motor axons of peripheral nerves while the latter targets either the dorsal column or the dorsal roots. FES is usually administered through self-adhesive electrodes placed over muscle motor points on the skin and an external stimulator (e.g., The Bionic Glove by Neuromotion, Edmonton, Canada; MyndMove Inc., Ontario, Canada). Implantable FES approaches work by electrodes placed either epimysially (Freehand system, NeuroControl, Cleveland, USA) or inside the muscle (Fesmate, NEC Medical Systems, Tokyo, Japan) and powered externally. FES and ARC Therapy studies have shown some promising results in hand and/or arm function improvement [18, 19].

In summary, although most obvious, motor deficits comprise only one part in the vast spectrum of problems faced by individuals with SCI. Complications associated with this condition are significant and extend well beyond motor deficits as described in this section. Treatment options available for these individuals are limited and none so far offers a prognosis of durable fully restored function. Electrical stimulation may not only restore function but also improve other co-morbid conditions associated with SCI such as spasticity, sensory deficits, cardiovascular abnormalities and autonomic issues, thereby improving the overall quality of life of these individuals.

8.4 Neurostimulation and ARC Therapy – Feasibility Data

While standard of care treatments like FTP result in limited functional gains in chronic cervical SCI, early evidence indicates that, when paired with electrical stimulation, FTP can result in significant improvement in both strength and function [18]. Specifically, the use of ARC Therapy, a form of transcutaneous spinal cord stimulation, has shown functional improvements that last beyond the treatment period ("carryover effect") [21, 22]. Aside from motor and sensory changes, improvements in spasticity, bladder and bowel function have also been reported [19, 21]. Additionally, improvements in AIS scale by at least 1 point (n=3) and/or neurological level of injury (n=9) by at least one dermatomal level have also been reported. More importantly, none of these studies reported any significant adverse events. Finally, since the therapy is non-invasive and any potential stimulation-induced adverse events are either transient or reversible, it presents a favorable risk-benefit ratio as a potential treatment option for individuals with chronic cervical SCI.

Typically, performance gains from FTP alone are minimal and reach a measurable plateau after just a few sessions [22]. Feasibility study data suggests that an incremental benefit is obtained from subsequently administering ARC Therapy in conjunction with FTP. Thus, it is possible to administer these therapy schemes in a sequential fashion without the results being confounded by carryover effect.

8.5 Rationale for the Choice of Primary Endpoint

The choice of study endpoints for this pivotal study was guided by multiple factors:

- Safety,
- Relevance to UE function,

- Capture improvements in both strength and function, and
- Magnitude of changes that are clinically meaningful.

To align on specific outcome variables, the recommendations for SCI common data elements (CDE) proposed by National Institute of Neurological Disorders and Stroke (NINDS) were reviewed. The SCI CDE Working Group is supported by the NINDS CDE Team. This group recommended standardized, validated instruments for SCI research. The International Spinal Cord Society (ISCoS) and the American Spinal Injury Association (ASIA) have since collaborated to incorporate the International SCI data sets into the NINDS CDEs. Next, the findings from stakeholder groups like North American Spinal Cord Injury Consortium (NASCIC), Praxis Spinal Cord Institute and Neurotech Network were reviewed to identify the priorities of those suffering from the condition. Lastly, discussions with thought leaders in SCI research were also solicited.

Based on these inputs, it was determined that a single composite primary endpoint that includes ISNCSCI, GRASSP, CUE-T, pinch and grasp strengths would be ideal to capture hand/arm improvements in both strength and function domains.

8.6 Rationale for the Choice of Secondary and Observational Endpoints

To get a complete picture of the improvements with ARC Therapy, additional measures that reflect functional recovery, quality of life, and autonomic function will be captured as secondary and observational endpoints. Secondary endpoints were chosen based on the following criteria:

- Demonstrate the incremental effectiveness of ARC Therapy over standard of care
- Capture potential additional benefits from the treatment, and
- Patients' perception of their condition before and after the treatment administration

Thus, secondary endpoints were chosen to assess the superiority of ARC Therapy + FTP over FTP alone and will perform a hierarchical superiority assessment of individual metrics of strength, function and quality of life.

Observational endpoints were primarily focused on patient reported outcomes (PROs) used as "valid scientific evidence of safety and/or effectiveness which is complementary to other evidence of clinical outcomes and/or biomarkers" [23]. The following table summarizes eight out of the 10 observational endpoints that used PROs with clearly laid out concept of interest, context of use and fit-for-purpose to report improvements (Table 2).

Up-LIFT PROs			
**Patient Reported Outcome**	**Concept of Interest (COI)**	**Context of Use (COU)**	**Fit-for-purpose**
Numerical Rating Scale (NRS)	Pain	Self reported pain intensity	Bryce et al. J Spinal Cord Med. 2007; 30:421-440 https://www.ncbi.nlm.nih.gov/pmc/articles/PMC2141724/pdf/i1079-0268-30-5-421.pdf
International Spinal Cord Injury Pain Data Set (ISCIPDS)	Pain	Captures 3 major areas of pain, its intensity and interference with activities, sleep and mood	Widerstrom-Noga et al. Spinal Cord. 2008; 46: 818-823 https://www.nature.com/articles/sc200864
Medical Outcomes Study (MOS) Sleep Scale	Sleep	Self reported sleep duration, quality and interference	Jensen et al.Rehabil Psychol. 2009; 54(3): 323-31. https://www.ncbi.nlm.nih.gov/pmc/articles/PMC2848672/pdf/nihms-187503.pdf
Penn Spasm Frequency Scale (PSFS)	Spasm	Self reported freq and severity of spasms	Penn RD et al. NEJM. 1989; 320(23): 1517-1521. Aydin G et al. Am J Phys Med Rehabil 2005;84:584-592.
5-dimension EuroQoL questionnaire (EQ-5D-5L)	Quality of life	Mobility, self-care, usual activities, pain/discomfort and anxiety/depression	**Acute study** by R. Koga et al. Spinal Cord. 2019 57(11):960-965.
The World Health Organization Quality of Life (WHOQOL)-BREF	Quality of life	Six domains: physical, psychological, level of independence, social relationships, enviroment, spirituality/religion/personal beliefs	Jang et al. Arch Phys Med Rehabil. 2004; 85: 1890-1895. https://www.archives-pmr.org/article/S0003-9993(04)00473-3/pdf
Patient Health Questionnaire (PHQ-9)	Depression	Depression severity	http://scireproject.com/wp-content/uploads/Research-Summary-_PHQ-9-v.7.0.pdf
Global Impression of Change	Quality of life	Perceived overall improvement	Bryce et al. J Spinal Cord Med. 2007; 30:421-440 (**Limited to GIC in pain**) https://www.ncbi.nlm.nih.gov/pmc/articles/PMC2141724/pdf/i1079-0268-30-5-421.pdf

Table 2. List of PROs used in the Up-LIFT study

8.7 Study Design Considerations

A variety of study designs were considered before finally selecting a sequential single arm study.

Randomized, sham-controlled study: A randomized controlled trial (RCT) is the acknowledged gold standard to assess the efficacy of a new therapy. However, it may not be the most pragmatic study design for all disease conditions and therapies.

As discussed previously in the report, most improvement in motor performance of individuals with cervical SCI receiving standard of care FTP therapy occurs in less than a year and plateaus around 18 months post-injury. With physical therapy as the only available standard of care treatment, this study design faces several challenges.

First, as demonstrated in preliminary studies, efficacious ARC Therapy is usually accompanied by mild sensations of tingling in the necks, arms and other dermatomal distributions [20]. This makes blinding impossible and hence precludes the use of LIFT System in a sham-controlled study. Second, It is highly unlikely a placebo response of the magnitude needed to rise to the level of meeting the primary effectiveness endpoint would be seen in this patient population [24]. Next, this design poses challenges with patient recruitment and retention in the study. Finally, since the individuals with SCI are aware of the limitations of SOC, it may result in significant bias against the control arm, especially if they are not offered the experimental treatment/therapy as part of the study.

Crossover study: A potential solution to an RCT is a crossover study design where subjects from both arms have the option to crossover to the treatment that they were not originally randomized to. However, this assumes that the effects of the first treatment have been "washed out" before the crossover treatment is administered.

Preliminary studies have shown that 4-8 weeks of ARC Therapy with FTP resulted in durable performance gains in measures of UE performance including pinch and grasp forces (Figure 2) [21] persisting after several months with no further treatment. None of the study subjects tested reverted to their pre-training baseline. While these reports only included 7 subjects, the effect was nonetheless sturdy and repeatable. Thus, a crossover design could potentially confound the gains reported in the control arm population that crossed over from the ARC Therapy arm due to a carry-over effect.

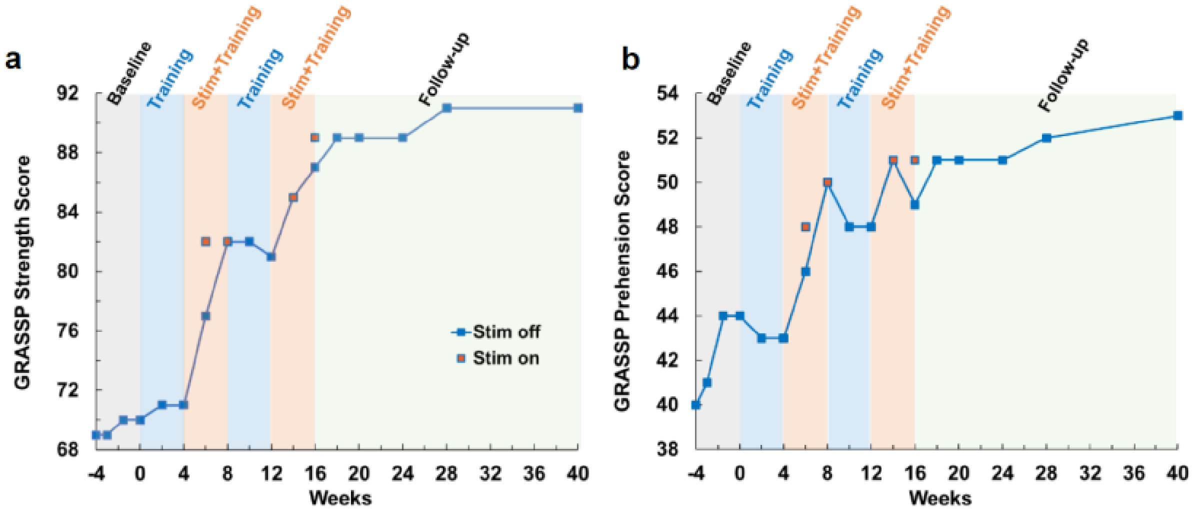

Figure 2. Strength and prehension scores from one of the study participant from Inanici et al. [19]

Single arm sequential study: Given these constraints, ONWARD Medical, Inc. chose a pragmatic, prospective, sequential study design for the Up-LIFT study. This study design is viable provided that it is possible to establish that the treatment effect during a wash-in phase may be distinguished from subsequent treatment effects from the investigational therapy. All subjects will be initially exposed to a fixed, 2-month SOC course of treatment (wash-in phase) during which daily measurement of UE performance (box and block test) will be used to assess the gross manual dexterity of subjects. As shown earlier, most people with incomplete tetraplegia show minimal improvement in function during the second year after traumatic SCI [12]. It is expected that the rate of improvement in the box and blocks test scores will diminish over the wash-in period, thus indicating that subjects have achieved "treatment plateau" before proceeding to the second phase of the study where they will be administered the ARC Therapy along with FTP. In this manner, it will be feasible to differentiate performance gains achieved with SOC therapy during the first study phase from those achieved with additive ARC Therapy in the second phase.

9 References

[1] Cohen, J. (1988). Statistical Power Analysis for the Behavioral Sciences. Second edition. Lawrence Erlbaum Associates.

[2] Wu, X., Liu, J., Tanadini, L. G. et al. (2015). Challenges for defining minimal clinically important difference (MCID) after spinal cord injury *Spinal Cord*. 53: 84-91.

[3] National Spinal Cord Injury Statistical Center (NSCISC). Spinal Cord Injury Facts and Figures at a Glance. Available from: https://www. nscisc.uab.edu/Public/Facts%202016.pdf, 2016.

[4] Anderson, K. (2004). Targeting recovery: priorities of the spinal cord-injured population. *J Neurotrauma*, 21(10):1371–83. doi:10.1089/neu.2004.21.1371.

[5] Lo, C., Tran, Y., Anderson, K., Craig, A., & Middleton, J. (2016). Functional priorities in persons with spinal cord injury: using discrete choice experiments to determine preferences. *J Neurotrauma*, 33(21):1958–68. doi:10.1089/ neu.2016.4423.

[6] Snoek, G., IJzerman, M., Hermens, H., Maxwell, D., & Biering-Sorensen, F. (2004). Survey of the needs of patients with spinal cord injury: impact and priority for improvement in hand function in tetraplegics. . *Spinal Cord*, 42:526–32. doi:10.1038/sj.sc.3101638.

[7] Smaby, N., Johanson, M., Baker, B., Kenney, D., Murray, W., & Hentz, V. (2004). Identification of key pinch forces required to complete functional tasks. *J Rehabil Res Dev*, 41(2):215–24. doi:10.1682/JRRD.2004.02.0215.

[8] Sezer, N., Akkuş, S., & Uğurlu, F. G. (2015). Chronic complications of spinal cord injury. *World J Orthop*, January 18; 6(1): 24-33.

[9] Kirshblum, S.C., O'Connor, K. C. (1998). Predicting Neurologic Recovery in Traumatic Cervical Spinal Cord Injury. *Arch Phys Med Rehabil*.79: 1456-66.

[10] Fawcett, J.W., Curt, A., Steeves, J.D., Coleman, W. P., Tuszynski, M. H. et al. (2007). Guidelines for the conduct of clinical trials for spinal cord injury as developed by the ICCP panel: spontaneous recovery after spinal cord injury and statistical power needed for therapeutic clinical trials. *Spinal Cord*. 45: 190-205.

[11] Ditunno JF Jr, Cohen ME, Hauck WW, et al (2000). Recovery of upper-extremity strength in complete and incomplete tetraplegia: a multicenter study. *Arch Phys Med Rehabil*, 81: 389–93.

[12] Burns, A.S., Ditunno, J.F. (2001). Establishing Prognosis and Maximizing Functional Outcomes After Spinal Cord Injury: A Review of Current and Future Directions in Rehabilitation Management. *Spine*. 26(24S): S137-S145.

[13] Gomes-Osman, Y., Tibbett, J.A., Poe, B. P., Field-Fote, E.C. (2017). Priming for Improved Hand Strength in Persons with Chronic Tetraplegia: A Comparison of Priming-Augmented Functional Task Practice, Priming Alone, and Conventional Exercise Training. *Front Neurol.* 7: 242.

[14] Beekhuizen, K.S. (2005). New Perspectives on Improving Upper Extremity Function after Spinal Cord Injury. *J Neurological Physical Therapy*. 29(3):157-62.

[15] Friden, J., Gohritz, A. (2015). Tetraplegia Management Update. J Hand Surg Am. 40(12): 2489-500.

[16] Mehta, S., McIntyre, A, Dijkers, M. et al. (2014). Gabapentinoids are effective in decreasing neuropathic pain and other secondary outcomes after spinal cord injury: a meta-analysis. *Arch Phys Med Rehabil*. 95(11): 2180-6

[17] Davari, M., Amani, B., Amani, B., Khanijahani, A., Akbarzadeh, A., Shabestan, R. (2020). Pregabalin and gabapentin in neuropathic pain management after spinal cord injury: a systematic review and meta-analysis. *The Korean J Pain.* 33(1): 3-12.

[18] Hoffman, L.R., Field-Fote, E. C. (2013). Effects of practice combined with somatosensory or motor stimulation on hand function in persons with spinal cord injury. *Top Spinal Cord Inj Rehabil.* (4):288-99. doi: 10.1310/sci1904-288.

[19] Gad, P., Lee, S., Terrafranca, N. et al. (2018). Non-Invasive Activation of Cervical Spinal Networks after Severe Paralysis. *J Neurotrauma.* 35: 2145-58.

[20] Inanici, F., Samejima, S., Gad, P. et al. (2018). Transcutaneous Electrical Spinal Stimulation Promotes Long-Term Recovery of Upper Extremity Function in Chronic Tetraplegia. *IEEE Trans Neural Syst Rehabil Eng.*, 26(6):1272-1278.

[21] Inanici, F., Brighton, L., Samejima, S. et al. (2021) Non-invasive spinal cord stimulation restores hand function after paralysis. *IEEE Trans Neural Syst Rehabil Eng.*, 26(6):1272-1278. DOI 10.1109/TNSRE.2021.3049133.

[22] Tefertiller, C., et al. (2021). Advances in Non-Invasive Electrical Spinal Cord Stimulation to Promote Recovery in Chronic Spinal Cord Injury. Annual Meeting of the North American Neuromodulation Society.

[23] US Food and Drug Administration (2020). Principles for Selecting, Developing, Modifying, and Adapting Patient-Reported Outcome Instruments for Use in Medical Device Evaluation. Draft Guidance for Industry and Food and Drug Administration Staff, And Other Stakeholders.

[24] Sayenko, D., Rath, M., et al. (2018). Self-Assisted Standing Enabled by Non-Invasive Spinal Stimulation after Spinal Cord Injury. *J Neurotrauma*. 2019 May 1;36(9):1435-1450.
